# Seasonal and annual changes in the microbial communities of Ofunato Bay, Japan, based on metagenomics

**DOI:** 10.1038/s41598-021-96641-9

**Published:** 2021-08-26

**Authors:** Atsushi Kobiyama, Jonaira Rashid, Md. Shaheed Reza, Yuri Ikeda, Yuichiro Yamada, Toshiaki Kudo, Nanami Mizusawa, Saki Yanagisawa, Daisuke Ikeda, Shigeru Sato, Takehiko Ogata, Kazuho Ikeo, Shinnosuke Kaga, Shiho Watanabe, Kimiaki Naiki, Yoshimasa Kaga, Satoshi Segawa, Yumiko Tada, Tatsuya Musashi, Katsuhiko Mineta, Takashi Gojobori, Shugo Watabe

**Affiliations:** 1 Kitasato University School of Marine Biosciences, Minami-ku, Sagamihara, Kanagawa 252-0373 Japan; 2 Bangladesh Fisheries Research Institute, Freshwater Station, Mymensingh, 2201 Bangladesh; 3 Department of Fisheries Technology, Bangladesh Agricultural University, Mymensingh, 2202 Bangladesh; 4 National Institute of Genetics, Yata, Mishima, Shizuoka 411-8540 Japan; 5 Iwate Fisheries Technology Center, Kamaishi, Iwate 026-0001 Japan; 6 Iwate Inland Fisheries Technology Center, Hachimantai, Iwate 028-7302 Japan; 7 Computational Bioscience Research Center, King Abdullah University of Science and Technology, Thuwal, 23955-6900 Saudi Arabia

**Keywords:** Microbiology, Environmental sciences

## Abstract

Five years of datasets from 2015 to 2019 of whole genome shotgun sequencing for cells trapped on 0.2-µm filters of seawater collected monthly from Ofunato Bay, an enclosed bay in Japan, were analysed, which included the 2015 data that we had reported previously. Nucleotide sequences were determined for extracted DNA from three locations for both the upper (1 m) and deeper (8 or 10 m) depths. The biotic communities analysed at the domain level comprised bacteria, eukaryotes, archaea and viruses. The relative abundance of bacteria was over 60% in most months for the five years. The relative abundance of the SAR86 cluster was highest in the bacterial group, followed by *Candidatus* Pelagibacter and *Planktomarina*. The relative abundance of *Ca.* Pelagibacter showed no relationship with environmental factors, and those of SAR86 and *Planktomarina* showed positive correlations with salinity and dissolved oxygen, respectively. The bacterial community diversity showed seasonal changes, with high diversity around September and low diversity around January for all five years. Nonmetric multidimensional scaling analysis also revealed that the bacterial communities in the bay were grouped in a season-dependent manner and linked with environmental variables such as seawater temperature, salinity and dissolved oxygen.

## Introduction

Marine microbes such as prokaryotes and small eukaryotes perform a number of essential roles in the environment, affecting major biogeochemical processes^1–3^. Bacteria play especially important roles in the recycling of organic matter and transfer of nutrients and energy in the marine food web^4,5^. To date, various findings have been reported about the functions of bacteria in the abovementioned biogeochemistry of seawaters^6,7^. Microbial metagenomes are defined as the collective genomes of microbes found in natural environments^8^. Continuous progress in next-generation sequencing (NGS) has generated large metagenomic sequences from multiple species over time or space^9^. Whole genome shotgun (WGS) sequencing is the core technology for metagenomics research and provides new insights into the structure of microbial communities in various ecosystems^10–14^. Venter et al.^14^ first employed a WGS study in the Sargasso Sea near Bermuda and found a large number of previously unknown genes from at least 1,800 genomic species based on sequence relatedness. Later, the *Tara* Oceans project revealed vertical stratification with epipelagic community composition mostly driven by temperature, using 243 samples from 68 locations in epipelagic and mesopelagic waters across the globe^15^. The GEOTRACES cruise also provided information on the identity, diversity and functional potential of the marine microbial community in a particular place and time using 610 samples spanning diverse regions of the Atlantic and Pacific Oceans^16^. In addition to such studies revealing spatial differences in marine microbial communities, one of the other topics in studying marine microbes is seasonality in their communities, which sheds light on the mechanisms involved in processes regulating complex network interactions to understand the long-term responses of microbes to climate change^17,18^. Long-term series to observe the dynamics of marine microbial communities have been carried out in several locations worldwide irrespective of approaches with WGS or others, such as 16S rDNA terminal restriction fragment length polymorphism (T-RFLP) fragments and denaturing gradient gel electrophoresis (DGGE) patterns using DNAs of microbial cells collected from the environment^18–27^. These studies revealed that communities in surface water show clear seasonal variations and are more similar to each other during the season of the year, whereas the community composition is most different when comparing opposite seasons^18^. By contrast, the monthly plot for deeper water shows no obvious similarity. Based on long-term metagenomic monitoring for years, Galand et al.^27^ claimed that marine microbial diversity with seasonal changes in the communities reflects a tremendous diversity of microbial metabolism and highlights the genetic potential yet to be discovered in an ocean of microbes.

In contrast to the long-term monitoring stations described above, which face the open sea, Ofunato Bay is an enclosed bay in Iwate Prefecture located at the centre of the Sanriku *Rias* coast facing the northwestern Pacific Ocean of Japan at a latitude of 39°N. It is well known for aquaculture production of plankton feeding shellfish. Thus, Ofunato Bay is likely to be easily affected by anthropogenic activities and freshwater inflow from rivers. The bay also receives seasonal water currents from the Pacific Ocean, such as the cold Oyashio Current and Tsugaru Warm Current, together with the warm Kuroshio Current off the coast^28,29^. Such environmental conditions allow exchanges of water masses and discharge of nutrients, which have possible influences on microbial communities in the bay.

Because of the importance of Ofunato Bay, we previously conducted a WGS sequencing study on the microbial communities at different locations throughout the entire length of the bay using 0.2 µm-filter fractions of seawater samples collected monthly over one year in 2015: the main target of that study was free-living bacterioplankton^30–34^. As a result, the most frequently recovered bacterial sequences belonged to Proteobacteria and were predominantly comprised of the genera *Planktomarina* and *Candidatus* (*Ca*.) Pelagibacter. However, it has remained unknown whether the bacterial communities in the bay may reproduce similar seasonal fluctuations annually.

Here, we carried out WGS sequencing on seawater samples collected monthly from the bay for five years from 2015 to 2019, including the previously reported 2015 dataset. The present study employed Blastx instead of Blastn, the latter of which was used in our previous study, for annotation of obtained reads and revealed that the SAR86 cluster was dominant, although the other two major bacterial genera, *Planktomarina* and *Ca*. Pelagibacter, were also dominant from 2015 to 2019.

## Materials and methods

### Seawater sampling and processing

**Figure 1:**
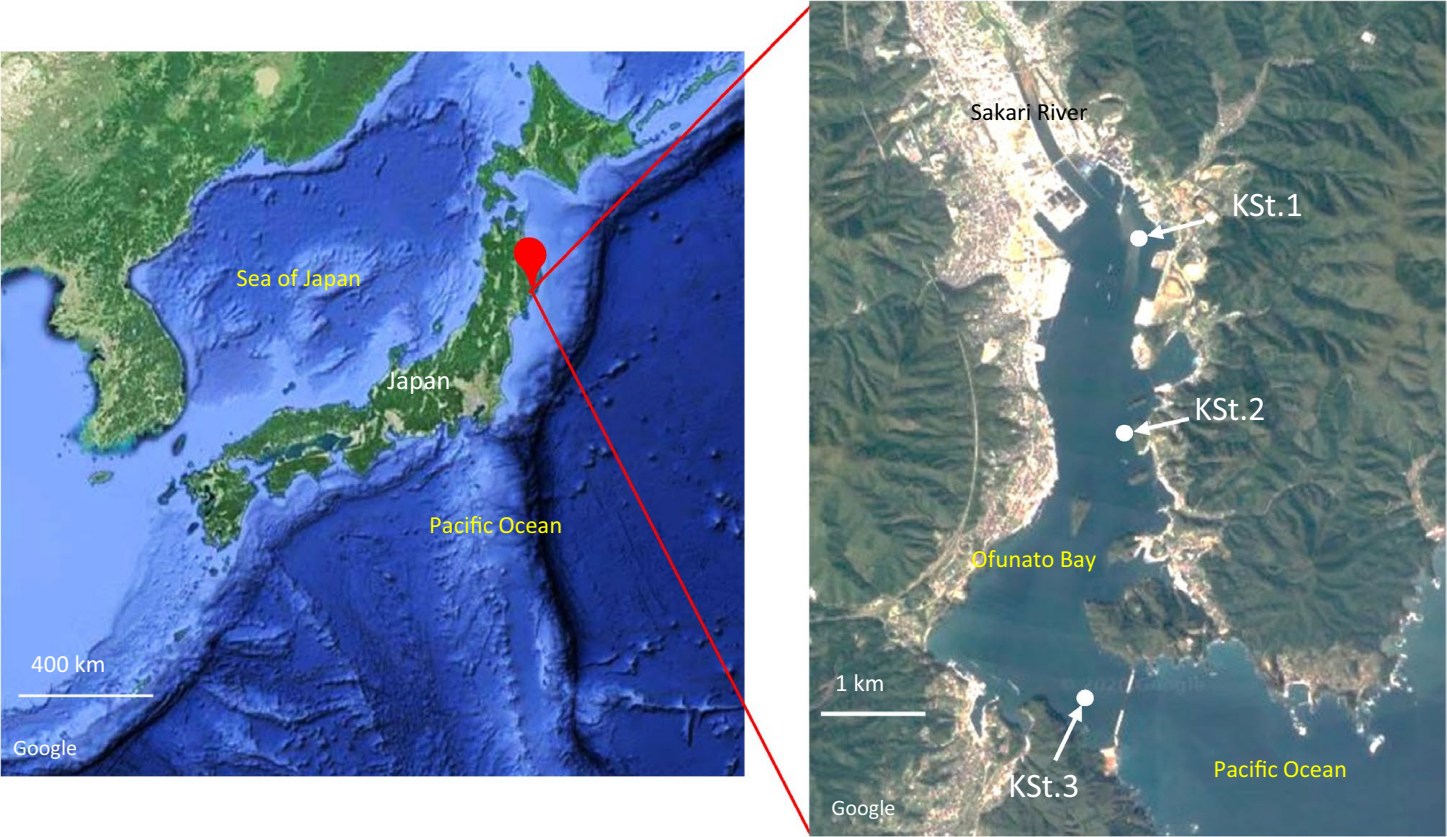
Maps showing stations for seawater sample collection in Ofunato Bay, Iwate Prefecture, Japan. KSt. 1 (innermost area; 141.73245 E, 39.063370 N; average depth, 10.3 m), KSt. 2 (middle area; 141.73254 E, 39.044612 N; average depth, 25.3 m) and KSt. 3 (bay entrance area; 141.72820E, 39.019030 N; average depth, 38.5 m) are marked with arrows. The bay is 6 km in length and 1.5 km in width, with a maximum depth of 38.5 m. Maps were created with Google (https//goo.gl/maps).

Sample collection was performed according to Reza et al.^32^. Briefly, approximately 8 L of seawater in Ofunato Bay, Iwate Prefecture, Japan, was collected monthly from 2015 to 2019 with a Van Dorn water sampler. Sampling was conducted at the three locations including KSt. 1 (the most inner area), KSt. 2 (the centre area) and KSt. 3 (the mouth of the bay), at two sampling depths, 1 m (KSt. 1, KSt. 2 and KSt. 3) and 8 (KSt. 1) and 10 m (KSt. 2 and KSt. 3) (Fig. 1). The area around KSt. 1 was predicted to be strongly influenced by the Sakari River. KSt. 3 was located near a wave-braking barrier protecting against tsunamis from the Pacific Ocean. KSt. 2 was located in the oyster aquaculture field^32^.

Environmental factors such as seawater temperature, salinity and dissolved oxygen (DO) were measured on site at each sampling point using a RINKO AAQ176 water quality profiler (JFE Advantech Co., Ltd., Nishinomiya, Japan). The seawater samples were pretreated through a 100-µm filter to remove debris and transported to Sanriku Coastal Education and Research Center, Kitasato University School of Marine Biosciences, Iwate Prefecture, under refrigerated conditions. The seawater samples collected were passed in sequence through 20-, 5-, 0.8- and 0.2-µm pore size filters (Merck Millipore Ltd., Tullagreen, Ireland). Approximately 250 mL of seawater was also collected from each sampling point and used for measurement of the concentrations of inorganic nutrients such as dissolved inorganic nitrogen (NO_2+3_-N = NO_2_-N + NO_3_-N; NH_4_-N), phosphate (PO_4_-P) and silicate (SiO_2_-Si), along with the concentrations of chlorophyll *a* (chl-*a*), according to Yamada et al.^29^.

The numerical abundance of the heterotrophic bacteria and cyanobacteria (autotrophic bacteria) was determined in seawater samples preserved with glutaraldehyde (final concentration; 1%) by epifluorescence microscopy according to Hamasaki et al.^35^. It should be noted that the second most abundant cyanobacterial genus after *Synechococcus*, *Prochlorococcus* cannot be correctly quantified by this method^36^. The heterotrophic bacteria were stained with 4,6-diamidino-2-phenylindole (DAPI, final concentration at 0.5 µg/mL) and filtered onto 0.2-µm black Nuclepore filters (GE Healthcare, Chicago, IL, USA). Cyanobacteria were filtered with another 0.2-µm Nuclepore filter. The samples were inspected using UV excitation for heterotrophic bacterial counting and green excitation for cyanobacterial counting.

### DNA extraction and whole genome shotgun sequencing

WGS sequencing was employed to determine nucleotide sequences of cells trapped on 0.2-μm filters according to Reza et al.^32^. Briefly, the cells trapped on the filters were frozen, stored and subjected to DNA extraction after thawing using the Power Water isolation kit (MO BIO Laboratories Inc., Carlsbad, CA, USA), according to the manufacturer’s instructions at the Sanriku Coastal Education and Research Center. DNA concentrations were quantified with a Qubit dsDNA HS assay kit (Invitrogen, Carlsbad, CA, USA) and read with a Qubit Fluorometer (Invitrogen). The DNA libraries for shotgun sequencing were prepared in the laboratory at the Sagamihara campus in Kitasato University School of Marine Biosciences, Kanagawa Prefecture, using the Nextera XT DNA sample preparation kit (Illumina Inc., San Diego, CA, USA). The quality and sizes of the products were assessed with a Bioanlyzer 2100 (Agilent Technologies, Santa Clara, CA, USA). Finally, 0.2 µg of each of the DNA libraries prepared for various sampling points (6 points per month and 360 points for 5 years) was sequenced with an Illumina MiSeq sequencing system using MiSeq reagent kit V3 for 600 cycles (Illumina Inc.).

### Sequencing analysis

The WGS reads were first joined by overlapping forward and reverse reads using the FLASH software^37^ (version 1.2.10, https://ccb.jhu.edu/software/FLASH/) (minimum overlap = 10; maximum overlap = 65; maximum mismatch density = 0.25; allow outie pairs = false; cap mismatch quals = false; combiner threads = 20; input format = FASTQ, phred_offset = 33; output format = FASTQ, phred_offset = 33) and further processed using CLC Genomic Workbench (version 8) for trimming low-quality reads of < 50 bp (*P error* limit = 0.05; Q score = 30). Individual reads were paired together to sequence both ends of a fragment and generate high-quality sequence data of paired-end reads to facilitate downstream bioinformatics. After the quality-control pass, all datasets were compared to the NCBI nr database using a diamond BLASTx search with default settings (fast mode; e-value = 0.001). Taxonomic analysis was then performed using MEGAN software (MEGAN Ultimate Edition, version 6.21.5, https://computomics.com/megan.html) with the lowest common ancestor (LCA) parameters (minimum score = 50.0; maximum expected = 0.010; minimum percent identity = 0.0; top percent = 10.0; minimum support percent = 0.05; minimum support = 0; LCA algorithm = naive; percent cover = 100.0) (megan-map-Jan2021-ue.db). Comparative analysis in MEGAN was also performed after normalizing the counts and exporting these data to csv format. The relative abundances of bacterial genera in the bacterial communities were determined based on the taxonomy data where unclassified group, Oligotrophic Marine Gammaproteobacteria (OMG) group and environmental samples were included in “others”. The WGS reads were deposited into the DNA Data Bank of Japan (DDBJ) Sequence Read Archive under the accession numbers DRA005744, 005787, 010321, 010348 and 010349 for 2015, 2016, 2017, 2018 and 2019, respectively (Supplementary Table S1). The data in the csv format used for comparative analysis in MEGAN are available in the Kitasato University Repository (https://kitasato.repo.nii.ac.jp/?action=pages_view_main&active_action=repository_view_main_item_detail&item_id=632&item_no=1&page_id=13&block_id=21).

### Statistical analyses

Spearman’s rank correlation and clustering dendrogram (Bray–Curtis) were determined to demonstrate the relationships of the bacterial communities with environmental parameters using the vegan package^38^ in R software^39^ (RStudio version 1.3.1073, https://www.rstudio.com/products/rstudio/). Comparisons of dissimilarity among bacterial communities were carried out using nonmetric multidimensional scaling (NMDS) analysis. The NMDS plot was constructed from Morisita–Horn distances calculated by the vegan package in R using the data in the csv format exported from the MEGAN datasets. The correlations between the bacterial community and environmental parameters were calculated using “envfit” (vegan) and fit to the NMDS plot. The dendrograms were constructed by calculating the Bray–Curtis dissimilarity of bacterial communities using the vegan package in R. Seasonal changes in the alpha-bacterial community diversity at the genus level were determined by Shannon–Weaver^40^ and Simpson’s indices^41^ using the same vegan package mentioned above.

Significant differences between two variables (mean ± standard deviation) at the level of *P* < 0.01 were determined by Wilcoxon–Mann–Whitney test using R. Significant differences in Shannon–Weaver index between depths were examined by t-test using Excel.

### Seawater system analysis

The temperature–salinity (T–S) scatter diagram established by Hanawa and Mitsudera^42^ for variation in the water system distribution in the Sanriku coastal area was adopted to classify the seawater samples collected from each sampling point in Ofunato Bay into six water systems composed of surface, cold Oyashio, warm Kuroshio, Tsugaru Warm Current and bottom waters.

## Results

### Environmental parameters

The environmental parameters from 2015 to 2019 together with DNA concentrations recovered from KSt. 1, KSt. 2 and KSt. 3 at 1 m depth and 8 (KSt. 1) or 10 m depth (KSt. 2 and KSt. 3) are available in the Kitasato University Repository (https://kitasato.repo.nii.ac.jp/?action=pages_view_main&active_action=repository_view_main_item_detail&item_id=632&item_no=1&page_id=13&block_id=21) and their seasonal changes are depicted in Supplementary Figs. S1–S5. The relationships among the parameters analysed by Spearman’s rank correlation are shown in Supplementary Table S2.

While seawater temperature at the 1 m depth (14.58 ± 5.95 °C) was generally higher than that at the 8 or 10 m depth (11.70 ± 4.47 °C) during months when the temperature was gradually increasing from May or March (2015) to August (*P* < 0.01), the opposite tendency of higher temperature at the 8 or 10 m depth than at the 1 m depth was observed from September to February. The salinity also showed seasonal changes at the 1 m depth over five years, decreasing from March to October or November depending on the year. Low salinity was likely to be attributable to rainfall according to local weather records of Ofunato city^43^. The seasonal change in pH was not clear, although it appeared to be higher at the 1 m depth than that at the 8 or 10 m depth. DO increased from February to April every year and such changes were almost always positively correlated with those of pH and negatively correlated with those of seawater temperature (Supplementary Table S2). The concentration of NO_2+3_-N was generally higher at the 1 m depth than at the 8 or 10 m depth. Another nitrogen compound, NH_4_-N, showed concentrations generally lower than 4 µM. The concentration of phosphate (PO_4_-P) also showed seasonal changes and was generally low in June and July. The SiO_2_-Si concentration also showed seasonal changes and was roughly proportional to that of NO_2+3_-N (Supplementary Table S2). The concentrations of chl-*a* showed two peaks, one in spring and the other in autumn of each year.

When temperature and salinity data were plotted into the T–S scatter diagram, the seawater at the 8 or 10 m depth in March and April of 2015 and 2019 was clearly classified into the Oyashio water system (Supplementary Fig. S6).

### Abundances of heterotrophic bacteria and cyanobacteria

The abundances of heterotrophic bacteria were higher (*P* < 0.01) at the 1 m depths (9.45 × 10^5^ ± 4.67 × 10^5^ cells/L) than at the 8 or 10 m depths (7.07 × 10^5^ ± 3.15 × 10^5^ cells/L) irrespective of the station (Supplementary Figs. S7 and S8). Cyanobacterial abundance excluding that of *Prochlorococcus* showed peaks between August and November and decreased markedly in September, probably due to heavy rainfall in 2015 and slight rainfall in 2016. Cyanobacterial abundance tended to be generally higher at the 8 or 10 m depth than at the 1 m depth, although the difference was not statistically significant. The cyanobacteria abundance was negatively correlated with DO concentration (Supplementary Table S2).

### Metagenomic sequencing data

From the WGS reads of 324.645 G base pairs obtained over 5 years from the three stations in Ofunato Bay, 123.61 G base pairs of post QC sequences were recovered by paired-end read assembly (Supplementary Table S3). The ratio of assigned reads to total reads was 89–98% (Supplementary Table S4). Using these sequence data, the following analyses including those of biotic and bacterial communities were performed.

### Biotic communities at the domain level

As expected**,** the biotic communities determined for the cells trapped on 0.2-µm filters were comprised of bacteria, eukaryotes, archaea and viruses for seawater collected from Ofunato Bay at the 1 m and 8 or 10 m depths at KSt. 1, KSt. 2 and KSt. 3 where bacteria accounted for the largest proportion (Supplementary Figs. S9 and S10). The relative abundances of eukaryotes and archaea were quite low and no apparent difference was observed among locations, depths, seasons and years. Seasons defined here were set up according to the seawater temperature data in Ofunato Bay (Supplementary Figs. S1 and S4): winter from January to March, spring from April to June, summer from July to September and autumn from October to December.

### Bacterial communities at the genus level

**Figure 2:**
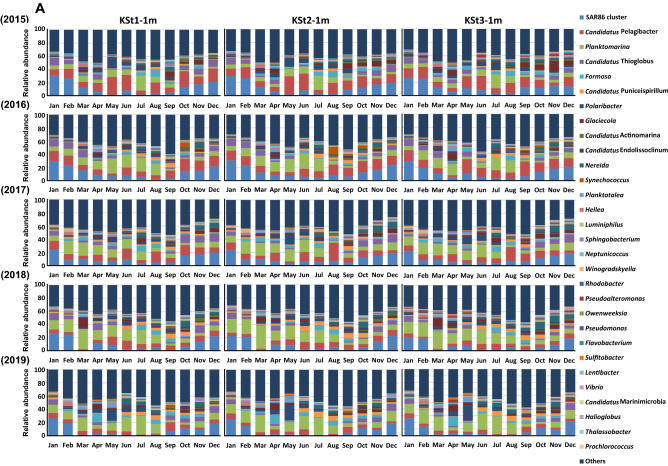

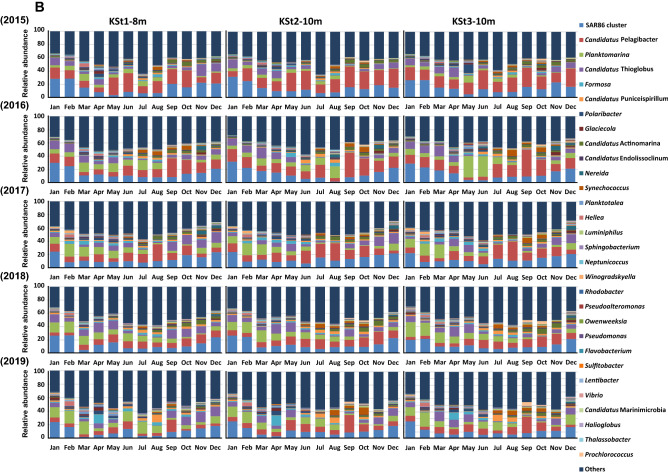
Seasonal and annual changes in the bacterial community at the genus level for seawater samples collected from the 1 m and 8 or 10 m depths at KSt. 1, KSt. 2 and KSt. 3 in Ofunato Bay from 2015 to 2019. While the seawater samples collected were serially passed through 20-, 5-, 0.8- and 0.2-µm pore size filters, only the cells trapped on the 0.2-µm filters were subjected to WGS sequencing. Panels (**A**) and (**B**) show data for the 1 m and 8 or 10 m depths, respectively. Refer to the legend of Fig. 1 for sampling stations.

At the genus level, the bacterial communities were mostly dominated by the SAR86 cluster, followed by *Ca*. Pelagibacter, *Planktomarina*, *Ca*. Thioglobus, *Formasa*, *Ca*. Puniceispirillum, *Polaribacter* and *Glaciecola* (Fig. 2). While SAR86, *Ca*. Thioglobus, and *Glaciecola* belong to Gammaproteobacteria, *Ca*. Pelagibacter, *Planktomarina* and *Ca*. Puniceispirillum belong to Alphaproteobacteria, and *Formosa* and *Polaribacter* belong to Bacteroidetes.

Among the dominant genera and their related clusters, the relative abundance of SAR86 was generally high in autumn and winter and low in spring and summer (*P* < 0.01), especially in 2019. *Ca*. Thioglobus showed seasonal changes similar to those of SAR86 (*P* < 0.01). The relative abundance of *Glaciecola* was high at the 1 m depth in autumn (*P* < 0.01) and in March or April (*P* = 0.01747) in a year-specific manner.

While the relative abundances of *Ca*. Pelagibacter and *Planktomarina* belonging to Alphaproteobacteria typically accounted for approximately 20% irrespective of location, depth, month and year, and the ratio of the relative abundance of the former to that of the latter genus decreased from 2015 to 2019 irrespective of the water depth. *Ca*. Puniceispirillum tended to be dominant in the spring and summer seasons (*P* < 0.01).

For dominant genera in Bacteroidetes, the relative abundance of *Formasa* was especially high in April 2019, irrespective of location and water depth. *Polaribacter* showed a high relative abundance in May 2015 at 10 m depth of KSt. 3 and May 2017–2019 at the 1 m depth at the three locations.

The relative abundance of cyanobacterium *Synechococcus* at the 1 m depth was high at KSt. 1 in October and at KSt. 2 in August 2016, whereas it was dominant at the 8 or 10 m depth between July and October from 2016 to 2019, mostly at KSt. 2 and KSt. 3. It was noted that another cyanobacterium, *Prochlorococcus*, was only marginal irrespective of location, depth, month and year. Interestingly, such seasonal changes in the abundance of cyanobacteria were roughly close to those obtained by epifluorescence microscopy (Supplementary Figs. S7 and S8), although the abundance of *Prochlorococcus* cannot be determined by this method^36^.

### Clustering and biodiversity analyses on seasonal and location-specific differences in the bacterial community

**Figure 3:**
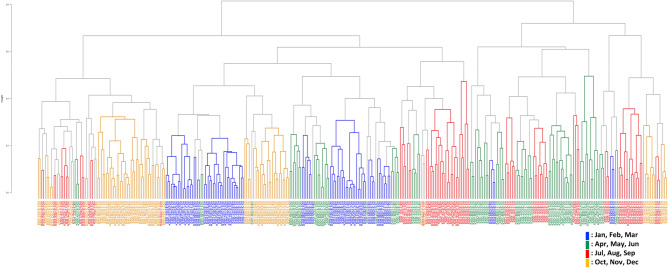
Clustering analysis of the bacterial community using datasets obtained from seawater samples collected from the 1 m depth (KSt. 1, KSt. 2 and KSt. 3) and 8 (KSt. 1) or 10 m (KSt. 2 and KSt. 3) depths in Ofunato Bay from 2015 to 2019. Refer to the legends of Figs. 1 and 2 for the sampling stations.

Seasonal and annual variations in the bacterial communities were further analysed by clustering dendrogram (Bray–Curtis) as shown in Fig. 3 and Supplementary Fig. S11, where four seasons are indicated with different colours. Clustering analysis datasets were generally separated into four seasons and overlapped with neighbouring seasons. Clustering with the same location was frequently observed, whereas datasets were clustered differently between 1 and 8 or 10 m depth, although the datasets from the same depth were mostly monophyletic (Supplementary Fig. S11).

**Figure 4:**
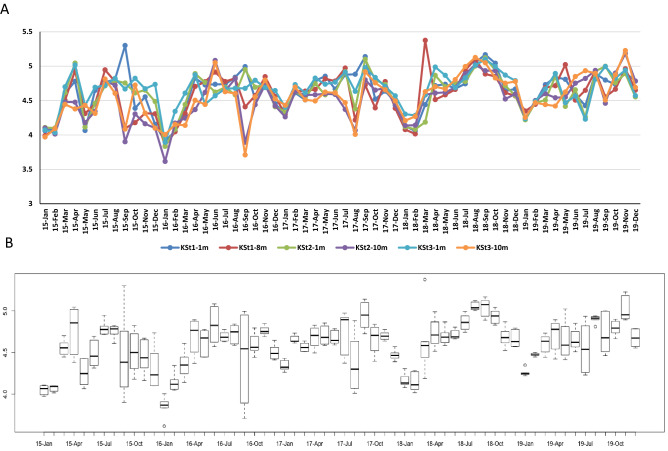
Seasonal changes in bacterial community biodiversity in Ofunato Bay plotted with the Shannon–Weaver diversity index. Seawater samples were collected monthly from the 1 m (KSt. 1, KSt. 2 and KSt. 3) and 8 (KSt. 1) or 10 m (KSt. 2 and KSt. 3) depths in Ofunato Bay from 2015 to 2019. Panels (**A**) and (**B**) show Shannon’s diversity index at three stations and two depths and the index from combined data for each month, respectively. Refer to the legend of Fig. 1 for the sampling stations.

Two different approaches to evaluate bacterial community biodiversity were applied in the present study: Shannon–Weaver and Simpson’s indices. Figure 4 shows the results with the Shannon–Weaver index, whereas Supplementary Fig. S12 shows those with Simpson’s index. Figures 4A and B show the respective results from the three different stations and the two different depths, together with those of combined data from the three stations and the two water depths, respectively. As shown in Fig. 4A, the three stations in Ofunato Bay exhibited location- and depth-dependent seasonal fluctuation patterns for five years. Although annual changes were observed in the datasets, the diversity index was roughly high around September and low in January. However, it was noted that the diversity index was lower at the 8 or 10 m depth than at the 1 m depth in September 2015 (*P* = 0.0515), September 2016 (*P* = 0.0329), August 2017 (*P* = 0.0431), September 2018 (*P* = 0.0462) and September 2019 (*P* = 0.0097).

When the data of the three stations were collectively analysed, seasonal variations in the bacterial community diversity were observed as shown in Fig. 4B: the highest variation was around September and lowest around January. Similar results to those of the Shannon–Weaver index were obtained with Simpson’s index as shown in Supplementary Fig. S12.

### Relationship of bacterial communities with environmental parameters

The relationships of the relative abundances of bacterial genera with environmental parameters for five sampling years were collectively analysed by Spearman’s rank correlation, and the obtained results are shown in Supplementary Table S5. In the bacterial communities, the relative abundance of the SAR86 cluster, the dominant bacterial group found in Ofunato Bay, showed a positive correlation with salinity, whereas that of *Polaribecter* exhibited a negative correlation. *Polaribacter* showed a positive correlation with DO concentration, as in the case of *Planktomarina*. The relative abundance of *Ca*. Puniceispirillum was positively correlated with water temperature. There were many genera that showed positive or negative correlations with water temperature, salinity and DO concentration. However, the relative abundances of *Ca*. Pelagibacter, *Ca*. Thioglobus, and *Formosa* showed no correlation with any environmental variables. In addition, no bacterial genera were correlated with nutrient or chl-*a* concentrations. The relative abundance of the autotrophic bacterial genus *Synechococcus* was positively correlated with seawater temperature and negatively correlated with DO concentration.

When seasonal changes in the bacterial community diversity were compared with those of chl-*a* concentration, the peaks of the latter were observed in nearly the same months or seasons as those of the former, although the peaks of chl-*a* concentrations somewhat preceded those of the bacterial community diversity (Supplementary Fig. S13).

### Relationship of the bacterial community with environmental parameters as revealed by NMDS analysis

**Figure 5:**
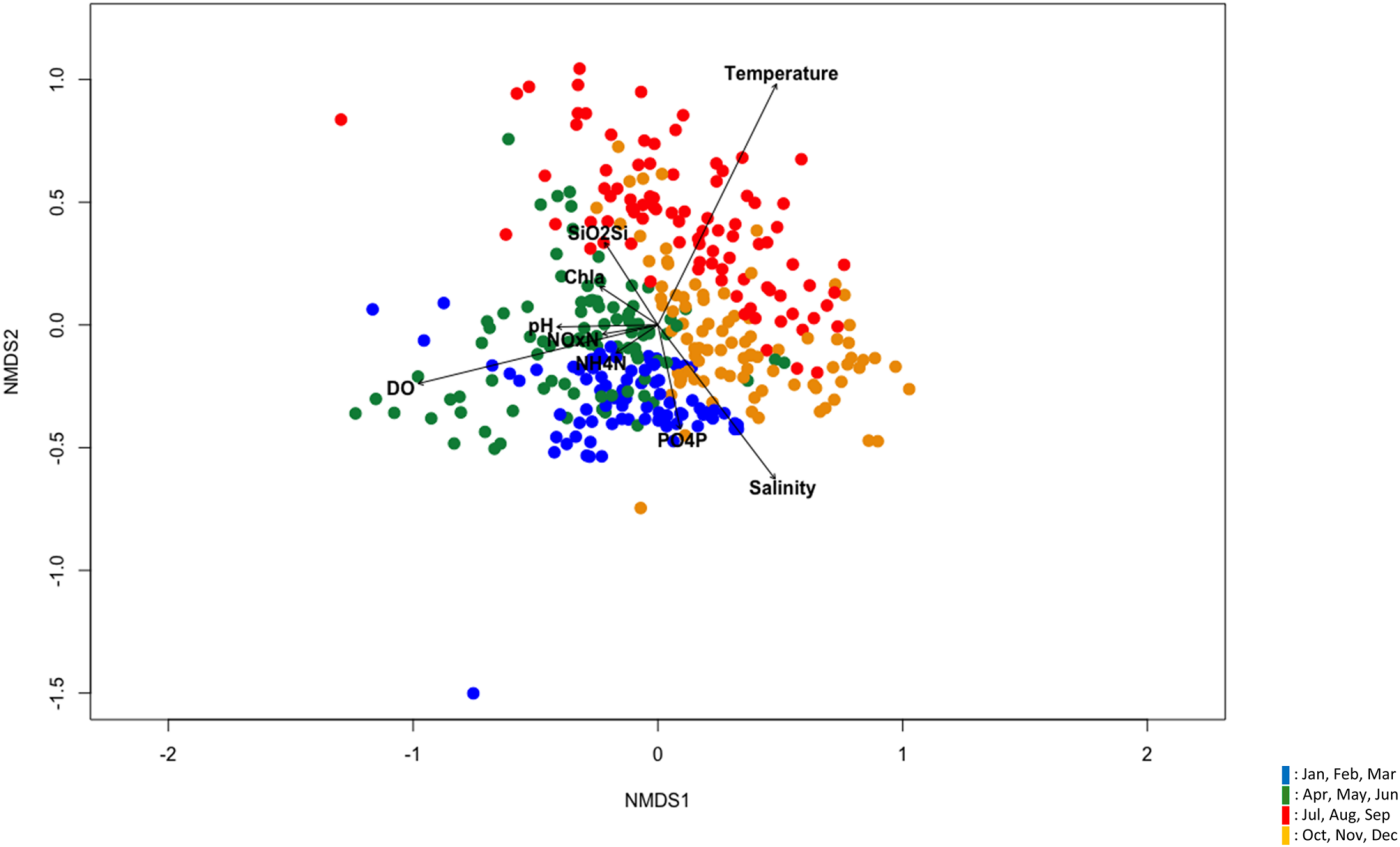
Nonmetric multidimensional scaling (NMDS) analysis of the relationship between bacterial communities and environmental parameters using datasets obtained from seawater samples collected from the 1 m (KSt. 1, KSt. 2 and KSt. 3) and 8 (KSt. 1) or 10 m (KSt. 2 and KSt. 3) depths in Ofunato Bay from 2015 to 2019. Refer to the legend of Fig. 1 for the sampling stations.

The results of NMDS analysis using all data obtained from the bacterial community at the three stations and two seawater depths in Ofunato Bay for five years are shown in Fig. 5. When the 12 months were grouped into four seasons as described in the clustering analysis, the datasets of winter and spring were clearly separated from those of summer and autumn. While seawater temperature showed a strong correlation with the bacterial communities (*r*^2^ = 0.70, *P* < 0.001) (Supplementary Table S6) in summer and autumn, the concentration of DO was correlated with the communities mostly in winter and spring (*r*^2^ = 0.60, *P* < 0.001). A high correlation with salinity was also shown at the border of autumn and winter (*r*^2^ = 0.37, *P* < 0.001). pH (*r*^2^ = 0.10, *P* < 0.001) and the concentrations of PO_4_-P (*r*^2^ = 0.11, *P* < 0.001) and SiO_2_-Si (*r*^2^ = 0.09, *P* < 0.001) showed weak correlations with the bacterial communities. Very weak correlations were observed for chl-*a* (*r*^2^ = 0.05, *P* < 0.002), NO_2+3_-N (0.03, *P* < 0.004) and NH_4_-N (0.02, *P* < 0.018). These results closely reflected those of Spearman’s rank correlation for the relationship of bacterial abundances at the genus level with environmental variables (Supplementary Table S5).

## Discussion

Long-term observations of the dynamics of marine microbial communities have been carried out in several locations worldwide^18–27^. In the present study, we also carried out a similar survey using WGS sequencing on seawater samples collected monthly at three stations from Ofunato Bay for five years from 2015 to 2019, including the previously reported 2015 dataset, and analysed the correlation of the obtained data with those of environmental factors. It has been repeatedly claimed that environmental samples, especially aquatic microbes, pose the greatest challenges for taxonomic assignment^14,44^. There is no gold standard for performing metagenomic experiments, especially regarding the bioinformatics used for analysis^24,45^. In the present study, all datasets were compared to the NCBI nr database using a BLASTx search. BLASTx is now frequently used, especially for functional analysis of metagenomic data^14,44,45^. The bacterial community diversity in Ofunato Bay, an enclosed bay, showed seasonal changes for all five years. NMDS analysis also revealed that the bacterial communities in the bay were grouped in a season-dependent manner and linked strongly with all environmental variables determined in the present study, especially seawater temperature, salinity and DO concentration.

The heterotrophic bacterial population was high at the 1 m depth and decreased at the 8 or 10 m depths (Supplementary Figs. S7 and S8). Their numbers increased during the spring and summer, but decreased in the winter. The cyanobacterial abundance was the highest in October and November and the lowest between March and May. These results were similar to those reported by Jiao and Ni^46^, and available reports suggest that close interactions exist between cyanobacteria and heterotrophic bacterial contents^47^.

The three stations in Ofunato Bay exhibited similar seasonal fluctuation patterns in bacterial community diversity for five years in which diversity was high around September and low around January (Fig. 4, Supplementary Fig. S12). Small peaks were found around April for the latter months as revealed by Shannon–Weaver and Simpson’s indices. Gilbert et al.^24^ reported that the alpha diversity of the observed operational taxonomic units (OTUs) was relatively constant across the time series, but showed distinct cyclical patterns with maxima in winter and minima in summer; in this study, bacterial richness was plotted by month spanning 6 years of marine water sampling in the Western English Channel. In contrast, our results demonstrated that the bacterial community diversity in Ofunato Bay was high in summer and low in winter as mentioned above. The bacterial community diversity in Ofunato Bay is likely to be influenced by phytoplankton blooms as revealed by seasonal changes in the chl-*a* concentration (Supplementary Fig. S13) and seasonal changes in the relative abundances of major bacterial genera such as SAR86 and *Ca*. Pelagibacter and *Planktomarina* (Fig. 2A). An alternate reason explaining the high diversity of bacterial community around September at the 1 m depth (Fig. 4) was considered to be due to rainfall in this month when salinity was low at the 1 m depth in Ofunato Bay (Supplementary Figs. S1 and S4). Interestingly, the diversity index at the 8 or 10 m depth was significantly lower in August or September every year. These results imply that the bacterial community is influenced by salinity changes even in deeper water layers (Supplementary Fig. S1). Alternatively, water temperature may have influenced the bacterial communities, because it was highest in September every year at the 8 or 10 m depth and reached the same level as that at the 1 m depth. Meanwhile, the numbers of heterotrophic bacteria at the 1 m depth were generally higher than those at the 8 or 10 m depth (Supplementary Figs. S7 and S8). Moreover, the peaks of chl-*a* concentration were found in nearly the same months as those of the Shannon–Weaver diversity index, although the latter peaks were slightly retarded compared to those of the former (Supplementary Fig. S13). Riemann et al*.*^48^ investigated the bacterial community composition during a diatom bloom in mesocosms. The abundance of bacteria was high during the peak period of chl-*a* concentration and then decreased again dramatically after the chl-*a* concentration peak. Teeling et al.^49^ also reported that the abundance of bacteria increased after phytoplankton blooms with the chl-*a* concentration peak, leading to a change in the bacterial composition. Therefore, it is predicted that phytoplankton blooms had a similar effect on the abundance and composition of bacteria in Ofunato Bay. It has been reported that Ofunato Bay formed a noticeable thermocline from May to August at a depth of 10 m from the surface in 2013 and 2014^29^. Such conditions may also affect bacterial community diversity.

Among dominant bacterial genera, SAR86, *Ca*. Thioglobus and *Glaciecola* belong to Gammaproteobacteria. Bacterial clones from the Sargasso Sea (hydrostation S) were assigned the prefix “SAR” and numbers ranging from 1 to 176^50^. Several genes from the Sargasso Sea were nearly identical to those from the Pacific Ocean, suggesting that these unorganized bacterial groups including SAR86 are distributed widely in the surface waters of subtropical oceans. This cluster was also found dominantly in Ofunato Bay. However, the present study did not demonstrate its correlation with water temperature, although it has been reported that the individual genomes of SAR86 display a temperature-dependent distribution^51^. Our survey resulted in a positive correlation with salinity, suggesting that Ofunato Bay contains some other individuals never reported before for SAR86.

*Ca*. Thioglobus strains, open ocean isolates from the SUP05 clade of marine Gammaproteobacteria, have been recently reported for their complete genome sequences^52,53^. Mixotrophic members of this clade have the potential to oxidize sulphur and fix carbon in marine biogeochemical cycles^52^. *Graciecola* forms a novel 16S rRNA lineage adjacent to the genus *Alteromonas*. It was first isolated from sea-ice cores from the coastal area of eastern Antarctica in a group of pigmented, psychrophilic, strictly aerobic chemoheterotrophs^54^.

*Formasa* and *Polaribacter* belong to Bacteroidetes. While isolates of *Formosa* were obtained from enrichment culture during degradation of the thallus of brown algae^55^, those of *Polaribacter* were obtained from sea ice and water from the Arctic and Antarctic as psychrophilic, gas vacuolate strains of the *Cytophage-Flavobacterium-Bacteroides* (CFB) phylogenetic group^56^. All these dominant genera in Ofunato Bay belonging to Gammaproteobacteria and Bacteroidetes have been recently collected, and many of their phylogenetic and biogeochemical properties have remained to be solved.

Compared with the dominant genera mentioned above, the following two genera belonging to Alphaproteobacteria have been extensively investigated for their properties. *Ca*. Pelagibacter^57,58^ is an abundant member of the SAR clade (SAR11), belonging to order Rickettsiales, class Alphaproteobacteria, whereas *Planktomarina*^59,60^ belongs to the RCA (*Roseobacter* clade-affiliated) cluster of family Rhodobacteraceae, class Alphaproteobacteria. They are both distributed worldwide with high divergence and were found to be dominant in Ofunato Bay in this study (Fig. 2). Giebel et al.^61^ reported that the success of *Planktomarina* has resulted from its ability to obtain supplementary metabolic energy during nutrient-deficient periods by employing oxygen-free photosynthesis and CO dehydrogenase when the bacterium is at the phase of decline or starvation. CO is abundant on the surface of seawater^62^, which could be one of the reasons why *Planktomarina temperata*, which has multiple sets of CO dehydrogenase, is abundant there.

The growth of *Ca*. Pelagibacter depends on reduced sulphur compounds such as dimethylsulfoniopropionate (DMSP), which originate from phytoplankton^63,64^. Kudo et al.^31^ reported that a high abundance of the DMSP catabolic gene dmdA in *Ca*. Pelagibacter ubique HTCC1062 in Ofunto Bay were correlated with spring phytoplankton blooms. *Ca.* Pelagibacter has been reported to have benefits with proteorhodopsins that can function as light-dependent proton pumps during carbon starvation^65^. Another dominant genus of Alphaproteobacteria in Ofunato Bay, *Ca*. Puniceispirillum, also contains a proteorhodopsin-containing SAR116-clade strain^66,67^.

A recent study demonstrated that the Oyashio Current significantly changed the composition and functional genes of the microbial communities in the western subarctic Pacific area^68^. In the present study, the relative abundance of *Ca*. Pelagibacter decreased gradually from 2015 to 2019 and that of *Planktomarina* gradually increased correspondingly (Fig. 2). This was an interesting finding because the cold Oyashio Current, which supplies nutrients and transports nutrient-rich waters as reflected by the high PO_4_-P concentrations in the present study (Supplementary Figs. S3 and S5), entered Ofunato Bay in late winter or early spring in both 2015 and 2019, as revealed by the T–S scatter diagram (Supplementary Figs. S6). Thus, several other environmental variables were thought to lead to different bacterial communities between the two years, especially the difference in the proportion of *Ca*. Pelagibacter and *Planktomarina*. One of the possibilities to explain such a difference was the alteration in chl-*a* concentration, which reflected diatom blooms. The chl-*a* concentration was much lower in 2015 than in 2019, including during the months of February to May (Supplementary Fig. S3). It has been reported that the dissolved organic carbon (DOC) released from phytoplankton during photosynthesis is directly utilized by planktonic bacteria as extracellular materials for their growth^69,70^.

However, the mechanisms by which the diatom bloom in 2015 was less marked than that in 2019 remain unknown. Moreover, the abundance of heterotrophic bacteria was markedly higher in 2015 than in 2019 (Supplementary Fig. S7), even though the chl-*a* concentration in 2015 was lower than that in 2019 (Supplementary Fig. S3). Several possibilities were considered to partly explain such discrepancies. One was the reconstruction of a large-scale breakwater at the mouth of Ofunato Bay between KSt. 3 and the Pacific Ocean to protect against tsunami disaster^29^. The previous breakwater was almost completely destroyed by a massive tsunami following the Great East Japan Earthquake off the Pacific coast in 2011. The reconstruction started in 2012 and was completed in 2017. This reconstruction would have facilitated nutrient flow from the rivers and sewage near Ofunato Bay, which is an enclosed bay and would have retained the nutrients. The other possibility is the presence of nutrient uptake competitors in the bay, such as small eukaryotes, including photosynthetic picoeukaryotes^71^, which would have been trapped on the 5.0 or 0.8-µm filters.

*Synechococcus* was also abundant especially at deeper water depths in Ofunato Bay from July to October in a year-dependent manner (Fig. 2). *Synechococcus* is the dominant primary producer along with *Prochlorococcus* in marine biogeochemistry and plays a significant role in energy-related ocean carbon fixation^72,73^. *Synechococcus* is generally more abundant in coastal waters than in oligotrophic open water, whereas *Prochlorococcus* is generally restricted to oligotrophic open ocean waters^72,74^.

While *Synechococcus* has been reported to show seasonal changes in its abundance, it has subclades or subclusters with different seasonal expression patterns^75^.

In conclusion, the bacterial community diversity in Ofunato Bay showed clear seasonal changes, high around September and low around January, when bacterial richness was plotted by month across 5 years. NMDS analysis revealed that the bacterial communities were linked strongly to seawater temperature, moderately to DO and salinity, and slightly to pH and concentrations of PO_4_-P, NO_2+3_-N, NH_4_-N, SiO_2_-Si and chl-*a*. The major bacterial genera in the bay were SAR86, *C*a. Pelagibacter and *Planktomarina*.

## Supplementary Information

Supplementary Legends.

Supplementary Figures.

Supplementary Tables.

## Data Availability

The data generated and/or analysed during the current study are available in the Kitasato University Repository. https://kitasato.repo.nii.ac.jp/?action=pages_view_main&active_action=repository_view_main_item_detail&item_id=632&item_no=1&page_id=13&block_id=21.
