# Supplementary material for: Seasonal and annual changes in the microbial communities of Ofunato Bay, Japan, based on metagenomics
Source: Sci Rep. 2021 Aug 26;11:17277. doi: 10.1038/s41598-021-96641-9 (PMC8390468; doi:10.1038/s41598-021-96641-9)
Supplement: Supplementary file 1 — Supplementary Legends. [file 41598_2021_96641_MOESM1_ESM.docx]

**Seasonal and annual changes in the microbial communities of Ofunato Bay, Japan, based on metagenomics**

**Atsushi Kobiyama^1^, Jonaira Rashid^1,2^, Md. Shaheed Reza^1,3^, Yuri Ikeda^1^, Yuichiro Yamada^1^, Toshiaki Kudo^1^, Nanami Mizusawa^1^, Saki Yanagisawa^1^, Daisuke Ikeda^1^, Shigeru Sato^1^, Takehiko Ogata^1^, Kazuho Ikeo^1,4^, Shinnosuke Kaga^5^, Shiho Watanabe^5^, Kimiaki Naiki^6^, Yoshimasa Kaga^6^, Satoshi Segawa^5^, Yumiko Tada^5^, Tatsuya Musashi^5^, Katsuhiko Mineta^7^, Takashi Gojobori^7,🖂^ & Shugo Watabe^1,🖂 🖂^**

^1^Kitasato University School of Marine Biosciences, Minami-ku, Sagamihara, Kanagawa 252-0373, Japan. ^2^Bangladesh Fisheries Research Institute, Freshwater Station, Mymensingh-2201, Bangladesh. ^3^Department of Fisheries Technology, Bangladesh Agricultural University, Mymensingh-2202, Bangladesh. ^4^National Institute of Genetics, Yata, Mishima, Shizuoka 411-8540, Japan. ^5^Iwate Fisheries Technology Center, Kamaishi, Iwate 026-0001, Japan. ^6^Iwate Inland Fisheries Technology Center, Hachimantai, Iwate 028-7302, Japan. ^7^King Abdullah University of Science and Technology, Computational Bioscience Research Center, Thuwal 23955-6900, Saudi Arabia. ^🖂^ e-mail: [takashi.gojobori@kaust.edu.sa](mailto:takashi.gojobori@kaust.edu.sa) ^🖂🖂^email: [swatabe@kitasato-u.ac.jp](mailto:swatabe@kitasato-u.ac.jp)

**Supplementary information**

**Supplementary Table S1**. Accession numbers registered in the DNA Data Bank of Japan (DDBJ) Sequence Read Archive.

**Supplementary Table S2**. Spearman's rank correlation coefficients for the relationships among environmental variables in Ofunato Bay. Correlation coefficients of >0.5 or <-0.5 are marked yellowish green. Numerals in parentheses indicate P values.

**Supplementary Table S3**. Annual summary of the sequence analysis of 5 year metagenomic data from Ofunato Bay.

**Supplementary Table S4**. Assigned reads in sequence analysis on the datasets collected monthly from 2015 to 2019 at three stations in Ofunato Bay, including KSt. 1 (innermost area), KSt. 2 (centre area) and KSt. 3 (bay entrance area) at two sampling depths, 1 m (KSt. 1, KSt. 2 and KSt. 3) and 8 (KSt. 1) or 10 m (KSt. 2 and KSt. 3).

**Supplementary Table S5**. Spearman’s rank correlation was used to determine the relationship of bacterial abundances at the genus level with environmental variables. Correlation coefficients of >0.5 or <-0.5 are marked yellowish green.

**Supplementary Table S6**. Correlations of bacterial communities with environmental parameters in NMDS analysis.

**Supplementary Fig. S1.** Seasonal and annual variations in temperature, salinity and pH in Ofunato Bay. Seawater samples were collected monthly from 2015 to 2019 from three locations including KSt. 1 (innermost area), KSt. 2 (centre area) and KSt. 3 (bay entrance area) at two sampling depths, 1 m (KSt. 1, KSt. 2 and KSt. 3) and 8 (KSt. 1) or 10 m (KSt. 2 and KSt. 3).

**Supplementary Fig. S2.** Seasonal and annual variations in DO, NO_2+3_-N and NH_4_-N in Ofunato Bay. Seawater samples were collected monthly from 2015 to 2019 from three locations including KSt. 1 (innermost area), KSt. 2 (centre area) and KSt. 3 (bay entrance area) at two sampling depths, 1 m (KSt. 1, KSt. 2 and KSt. 3) and 8 (KSt. 1) or 10 m (KSt. 2 and KSt. 3).

**Supplementary Fig. S3.** Seasonal and annual variations in PO_4_-P, SiO_2_-Si and chlorophyll *a* (chl-*a*) in Ofunato Bay. Seawater samples were collected monthly from 2015 to 2019 from three locations including KSt. 1 (innermost area), KSt. 2 (centre area) and KSt. 3 (bay entrance area) at two sampling depths, 1 m (KSt. 1, KSt. 2 and KSt. 3) and 8 (KSt. 1) or 10 m (KSt. 2 and KSt. 3).

**Supplementary Fig. S4.** Seasonal variations in temperature, salinity, pH, DO, NO_2+3_-N and NH_4_-N in Ofunato Bay. Monthly averaged values for five years from 2015 to 2019 are shown for seawater samples from three locations including KSt. 1 (innermost area), KSt. 2 (centre area) and KSt. 3 (bay entrance area) at two sampling depths, 1 m (KSt. 1, KSt. 2 and KSt. 3) and 8 (KSt. 1) or 10 m (KSt. 2 and KSt. 3).

**Supplementary Fig. S5.** Seasonal variations in PO_4_-P, SiO_2_-Si and chlorophyll *a* (chl-*a*) in Ofunato Bay. Monthly averaged values for five years from 2015 to 2019 are shown for seawater samples from three locations including KSt. 1 (innermost area), KSt. 2 (centre area) and KSt. 3 (bay entrance area) at two sampling depths, 1 m (KSt. 1, KSt. 2 and KSt. 3) and 8 (KSt. 1) or 10 m (KSt. 2 and KSt. 3).

**Supplementary Fig. S6.** T-S scatter diagrams for monthly temperature and salinity of seawater at 1 m and 8 or 10 m depths of KSt. 1 (innermost area), KSt. 2 (centre area) and KS. 3 (bay entrance area) in Ofunato Bay from 2015 to 2019. Seawaters were collected monthly and classified into the surface-layer water system (1), coastal Oyashio water system (2), Oyashio water system (3), Tsugaru Warm Current water system (4), Kuroshio water system (5) and cold lower-layer water system (6) according to Hanawa and Mitsudera (1987). Contour lines of sigma-t are also shown.

**Supplementary Fig. S7.** Seasonal and annual variations in the abundance of heterotrophic bacteria and cyanobacteria in Ofunato Bay. Seawater samples were collected monthly from 2015 to 2019 from three stations, including KSt. 1 (innermost area), KSt. 2 (centre area) and KSt. 3 (bay entrance area) at two sampling depths, 1 m (KSt. 1, KSt. 2 and KSt. 3) and 8 (KSt. 1) or 10 m (KSt. 2 and KSt. 3).

**Supplementary Fig. S8.** Seasonal variations in the abundance of heterotrophic bacteria and cyanobacteria in Ofunato Bay. Monthly averaged values for five years from 2015 to 2019 are shown for seawater samples from three locations, including KSt. 1 (innermost area), KSt. 2 (centre area) and KSt. 3 (bay entrance area), arranging two sampling depths, 1 m (KSt. 1, KSt. 2 and KSt. 3) and 8 (KSt. 1) or 10 m (KSt. 2 and KSt. 3).

**Supplementary Fig. S9.** Seasonal and annual changes in the biotic community at the domain level for seawater collected at the 1 m depth at KSt. 1, KSt. 2 and KSt. 3 in Ofunato Bay from 2015 to 2019. While seawater samples collected monthly were serially passed through 20-, 5-, 0.8- and 0.2-µm pore size filters, only cells trapped on the 0.2-µm filters were subjected to WGS sequencing.

**Supplementary Fig. S10.** Seasonal and annual changes in the biotic community at the domain level for seawater collected at the 8 m depth at KSt. 1 or the 10 m depth of KSt. 2 and KSt. 3 in Ofunato Bay from 2015 to 2019. While seawater samples collected monthly were serially passed through 20-, 5-, 0.8- and 0.2-µm pore size filters, only cells trapped on the 0.2-µm filters were subjected to WGS sequencing.

**Supplementary Fig. S11.** Annual clustering analyses on the bacterial community using datasets obtained from seawater samples collected from the 1 m depth (KSt. 1, KSt. 2 and KSt. 3) and the 8 (KSt. 1) or 10 m depth (KSt. 2 and KSt. 3) in Ofunato Bay from 2015 to 2019. While seawater samples collected monthly were serially passed through 20-, 5-, 0.8- and 0.2-µm pore size filters, only cells trapped on the 0.2-µm filters were subjected to WGS sequencing.

**Supplementary Fig. S12.** Seasonal changes in bacterial community biodiversity in Ofunato Bay plotted with Simpson’s diversity index. Seawater samples were collected monthly from 2015 to 2019 from three locations including KSt. 1 (innermost area), KSt. 2 (centre area) and KSt. 3 (bay entrance area) at two sampling depths, 1 m (KSt. 1, KSt. 2 and KSt. 3) and 8 (KSt. 1) or 10 m (KSt. 2 and KSt. 3). Panels A and B show Simpson’s diversity index at three stations and two depths and the index from combined data therefrom for each month, respectively.

**Supplementary Fig. S13.** Seasonal changes in bacterial community biodiversity in Ofunato Bay plotted with Shannon’s diversity index together with those of chlorophyll *a* concentrations. Seawater samples were collected monthly from 2015 to 2019 from three locations including KSt. 1 (innermost area), KSt. 2 (centre area) and KSt. 3 (bay entrance area) at two sampling depths, 1 m (KSt. 1, KSt. 2 and KSt. 3) and 8 (KSt. 1) or 10 m (KSt. 2 and KSt. 3). Panels A and B show Shannon’s diversity index at three stations and two depths and chlorophyll a (chl-*a*) concentrations, respectively.
