# Supplementary material for: Seasonal and annual changes in the microbial communities of Ofunato Bay, Japan, based on metagenomics
Source: Sci Rep. 2021 Aug 26;11:17277. doi: 10.1038/s41598-021-96641-9 (PMC8390468; doi:10.1038/s41598-021-96641-9)
Supplement: Supplementary file 2 — Supplementary Figures. [file 41598_2021_96641_MOESM2_ESM.pptx]

## Slide 1
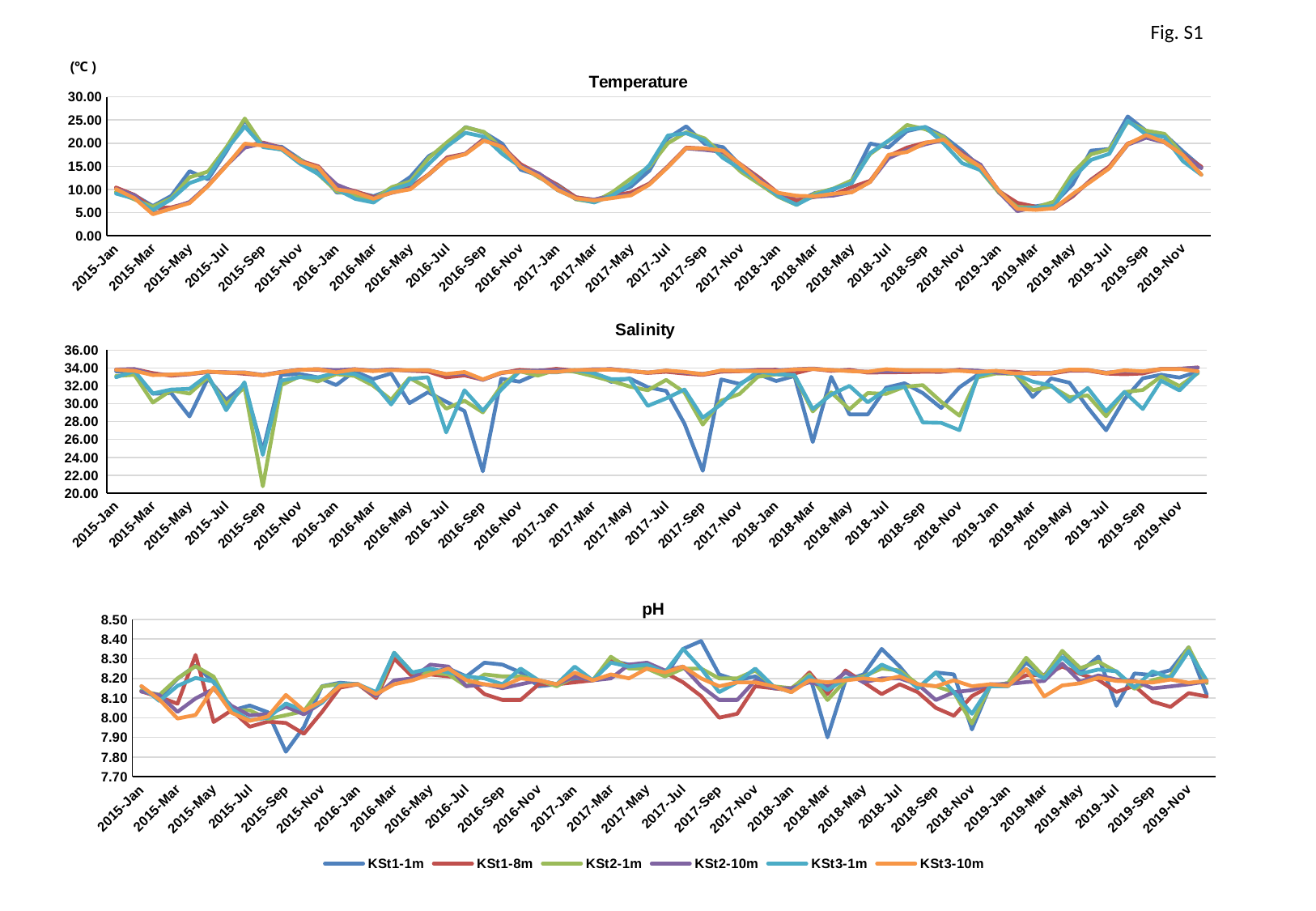

Fig. S1
### Chart: Temperature
| Category | KSt1-1m | KSt1-8m | KSt2-1m | KSt2-10m | KSt3-1m | KSt3-10m |
|---|---|---|---|---|---|---|
| 2015-Jan | 10.275 | 10.415 | 9.481000000000003 | 10.126 | 9.109 | 10.083 |
| 2015-Feb | 8.745999999999999 | 8.827 | 7.856999999999999 | 8.753 | 8.032 | 8.116000000000001 |
| 2015-Mar | 6.427999999999999 | 5.998 | 6.262 | 4.801 | 5.547 | 4.660999999999999 |
| 2015-Apr | 8.617999999999999 | 6.133999999999999 | 8.101 | 5.913 | 7.860999999999999 | 5.867999999999999 |
| 2015-May | 13.946 | 7.174999999999999 | 12.631 | 7.302999999999999 | 11.379 | 7.053999999999999 |
| 2015-Jun | 12.208 | 10.863 | 13.864 | 10.842 | 12.772 | 10.726 |
| 2015-Jul | 18.218 | 15.233 | 19.127 | 15.2 | 18.623 | 15.116 |
| 2015-Aug | 25.01 | 19.399 | 25.312 | 19.033 | 23.566 | 19.893 |
| 2015-Sep | 19.439 | 20.113 | 19.393 | 19.963 | 19.131 | 19.48 |
| 2015-Oct | 19.219 | 19.0 | 18.585 | 18.948 | 18.632 | 18.861 |
| 2015-Nov | 16.531 | 16.202 | 16.41 | 16.03 | 15.57 | 16.0 |
| 2015-Dec | 14.158 | 14.993 | 13.267 | 14.759 | 13.319 | 14.717 |
| 2016-Jan | 9.33 | 10.7 | 9.6 | 11.04 | 10.05 | 10.02 |
| 2016-Feb | 9.42 | 9.639999999999999 | 8.639999999999999 | 9.42 | 7.98 | 9.39 |
| 2016-Mar | 8.55 | 8.34 | 7.93 | 8.120000000000001 | 7.18 | 8.0 |
| 2016-Apr | 10.02 | 9.47 | 10.52 | 9.290000000000001 | 10.01 | 9.35 |
| 2016-May | 12.71 | 10.48 | 11.75 | 10.22 | 11.22 | 10.03 |
| 2016-Jun | 17.21 | 13.3 | 16.81 | 13.19 | 15.52 | 13.25 |
| 2016-Jul | 19.32 | 16.94 | 20.2 | 16.65 | 19.32999999999999 | 16.56 |
| 2016-Aug | 23.47 | 17.73 | 23.39 | 17.7 | 22.23 | 17.61 |
| 2016-Sep | 22.32 | 20.94 | 22.43 | 20.76 | 21.38 | 20.56 |
| 2016-Oct | 19.87 | 19.23999999999999 | 18.79 | 19.31 | 17.64 | 19.17000000000001 |
| 2016-Nov | 14.27 | 15.44 | 14.99 | 15.03 | 14.83 | 14.82 |
| 2016-Dec | 13.02 | 13.31 | 12.56 | 13.43 | 12.79 | 12.79 |
| 2017-Jan | 11.03 | 10.94 | 10.67 | 10.52 | 9.85 | 9.9 |
| 2017-Feb | 8.31 | 8.31 | 7.859999999999999 | 8.0 | 8.07 | 8.07 |
| 2017-Mar | 7.75 | 7.76 | 7.27 | 7.819999999999999 | 7.189999999999999 | 7.63 |
| 2017-Apr | 8.92 | 8.65 | 9.45 | 8.56 | 8.75 | 8.139999999999999 |
| 2017-May | 10.65 | 9.36 | 12.39 | 8.84 | 11.31 | 8.739999999999998 |
| 2017-Jun | 14.07 | 11.29 | 14.93 | 11.14 | 15.27 | 11.06 |
| 2017-Jul | 21.05 | 15.0 | 19.97 | 14.87 | 21.65 | 14.81 |
| 2017-Aug | 23.62 | 19.03 | 22.35 | 18.87 | 22.14 | 18.91 |
| 2017-Sep | 19.86 | 18.84 | 21.03 | 18.55 | 20.58 | 18.85 |
| 2017-Oct | 19.12 | 18.1 | 17.41 | 18.09 | 16.79 | 18.35 |
| 2017-Nov | 14.93 | 15.31 | 13.73 | 15.23 | 14.34 | 15.19 |
| 2017-Dec | 11.97 | 12.45 | 11.13 | 12.12 | 11.64 | 11.69 |
| 2018-Jan | 8.790000000000001 | 9.200000000000001 | 8.42 | 9.040000000000001 | 8.59 | 9.280000000000001 |
| 2018-Feb | 7.37 | 7.659999999999999 | 6.659999999999999 | 8.56 | 6.689999999999999 | 8.639999999999999 |
| 2018-Mar | 9.239999999999998 | 8.41 | 9.129999999999997 | 8.42 | 8.75 | 8.530000000000001 |
| 2018-Apr | 9.92 | 8.93 | 10.19 | 8.69 | 10.12 | 9.07 |
| 2018-May | 11.98 | 10.51 | 11.97 | 9.45 | 11.37 | 9.39 |
| 2018-Jun | 19.92 | 11.89 | 17.65 | 11.64 | 17.88 | 11.63 |
| 2018-Jul | 19.07 | 17.23 | 20.5 | 16.73999999999999 | 20.54 | 17.5 |
| 2018-Aug | 22.6 | 19.04 | 23.92 | 18.51000000000001 | 22.91 | 18.07999999999999 |
| 2018-Sep | 23.49 | 20.12 | 22.99 | 19.76 | 23.47 | 20.07 |
| 2018-Oct | 21.45 | 20.57 | 21.26 | 20.69 | 19.92 | 20.76 |
| 2018-Nov | 18.37 | 17.85 | 17.13 | 17.61 | 15.67 | 17.42 |
| 2018-Dec | 14.79 | 15.1 | 14.08 | 15.35 | 14.16 | 14.89 |
| 2019-Jan | 9.717999999999998 | 9.623000000000001 | 9.265 | 9.407 | 9.656 | 9.67 |
| 2019-Feb | 6.716 | 7.114999999999999 | 6.217 | 5.331 | 5.879 | 5.825999999999999 |
| 2019-Mar | 6.356999999999999 | 6.279 | 6.106999999999999 | 6.197999999999999 | 6.113 | 5.591 |
| 2019-Apr | 7.036 | 5.895999999999999 | 7.389 | 5.89 | 6.422 | 5.922 |
| 2019-May | 11.065 | 8.477 | 13.522 | 8.764000000000001 | 12.444 | 8.985 |
| 2019-Jun | 18.386 | 12.117 | 17.567 | 11.874 | 16.374 | 11.725 |
| 2019-Jul | 18.732 | 14.96 | 18.677 | 14.632 | 17.688 | 14.591 |
| 2019-Aug | 25.756 | 19.916 | 24.593 | 19.665 | 24.844 | 19.802 |
| 2019-Sep | 22.645 | 21.287 | 22.688 | 21.163 | 21.861 | 21.739 |
| 2019-Oct | 21.887 | 20.36199999999999 | 21.998 | 20.158 | 21.366 | 20.228 |
| 2019-Nov | 18.232 | 17.54 | 17.441 | 17.45 | 16.116 | 17.433 |
| 2019-Dec | 14.792 | 14.925 | 13.19904 | 14.60429 | 13.171 | 13.21 |(℃ )
### Chart: Salinity
| Category | KSt1-1m | KSt1-8m | KSt2-1m | KSt2-10m | KSt3-1m | KSt3-10m |
|---|---|---|---|---|---|---|
| 2015-Jan | 33.62 | 33.832 | 33.112 | 33.818 | 32.971 | 33.79 |
| 2015-Feb | 33.493 | 33.869 | 33.235 | 33.877 | 33.655 | 33.687 |
| 2015-Mar | 31.123 | 33.431 | 30.132 | 33.261 | 31.152 | 33.205 |
| 2015-Apr | 31.204 | 33.14100000000001 | 31.501 | 33.23900000000001 | 31.588 | 33.267 |
| 2015-May | 28.576 | 33.302 | 31.134 | 33.335 | 31.669 | 33.36 |
| 2015-Jun | 32.82700000000001 | 33.545 | 32.959 | 33.561 | 33.216 | 33.603 |
| 2015-Jul | 30.437 | 33.512 | 29.902 | 33.492 | 29.263 | 33.471 |
| 2015-Aug | 32.112 | 33.353 | 31.858 | 33.506 | 32.399 | 33.485 |
| 2015-Sep | 24.728 | 33.19 | 20.756 | 33.23300000000001 | 24.279 | 33.181 |
| 2015-Oct | 33.22300000000001 | 33.517 | 32.078 | 33.569 | 32.59 | 33.538 |
| 2015-Nov | 33.34 | 33.82 | 33.045 | 33.806 | 32.971 | 33.79 |
| 2015-Dec | 32.954 | 33.792 | 32.5 | 33.865 | 32.94 | 33.873 |
| 2016-Jan | 32.09 | 33.59 | 33.31 | 33.76 | 33.48 | 33.51 |
| 2016-Feb | 33.62 | 33.86 | 33.12 | 33.87 | 33.33 | 33.83 |
| 2016-Mar | 32.76 | 33.68 | 32.05 | 33.7 | 32.4 | 33.66 |
| 2016-Apr | 33.39 | 33.85 | 30.45 | 33.83 | 29.92 | 33.76 |
| 2016-May | 30.07 | 33.69 | 32.89 | 33.72 | 32.77 | 33.76 |
| 2016-Jun | 31.29 | 33.61 | 31.78 | 33.76 | 32.97 | 33.75 |
| 2016-Jul | 30.23 | 32.95 | 29.44 | 33.30000000000001 | 26.79 | 33.32 |
| 2016-Aug | 29.18 | 33.17 | 30.34 | 33.38 | 31.51 | 33.55 |
| 2016-Sep | 22.44 | 32.69 | 29.03 | 32.67 | 29.23 | 32.73000000000001 |
| 2016-Oct | 32.79 | 33.43 | 31.96 | 33.46 | 31.57 | 33.49 |
| 2016-Nov | 32.47 | 33.79 | 33.64 | 33.65 | 33.61 | 33.6 |
| 2016-Dec | 33.38 | 33.66 | 33.15 | 33.71 | 33.56 | 33.57 |
| 2017-Jan | 33.85 | 33.91 | 33.78 | 33.77 | 33.51 | 33.55 |
| 2017-Feb | 33.6 | 33.72 | 33.59 | 33.71 | 33.77 | 33.77 |
| 2017-Mar | 33.51 | 33.73000000000001 | 33.09 | 33.86 | 33.45 | 33.80000000000001 |
| 2017-Apr | 32.45 | 33.87 | 32.55 | 33.88 | 32.75 | 33.82 |
| 2017-May | 32.84 | 33.66 | 31.93 | 33.67 | 32.78 | 33.67 |
| 2017-Jun | 31.85 | 33.45 | 31.51 | 33.49 | 29.76 | 33.49 |
| 2017-Jul | 31.44 | 33.58 | 32.67 | 33.7 | 30.6 | 33.71 |
| 2017-Aug | 27.76 | 33.38 | 31.27 | 33.49 | 31.59 | 33.57 |
| 2017-Sep | 22.49 | 33.23000000000001 | 27.66 | 33.34 | 28.43 | 33.30000000000001 |
| 2017-Oct | 32.72 | 33.59 | 30.34 | 33.66 | 29.94 | 33.73000000000001 |
| 2017-Nov | 32.22 | 33.64 | 31.09 | 33.7 | 32.07 | 33.67 |
| 2017-Dec | 33.32 | 33.79 | 33.02 | 33.7 | 33.63 | 33.65 |
| 2018-Jan | 32.54 | 33.81 | 33.29 | 33.72 | 33.31 | 33.64 |
| 2018-Feb | 33.11 | 33.39 | 33.15 | 33.86 | 33.22 | 33.84 |
| 2018-Mar | 25.71 | 33.9 | 29.13 | 33.94 | 29.4 | 33.89 |
| 2018-Apr | 33.01 | 33.68 | 31.27 | 33.77 | 31.02 | 33.78 |
| 2018-May | 28.8 | 33.80000000000001 | 29.37 | 33.69 | 32.0 | 33.65 |
| 2018-Jun | 28.8 | 33.48 | 31.2 | 33.58 | 30.16 | 33.58 |
| 2018-Jul | 31.8 | 33.52 | 31.1 | 33.6 | 31.59 | 33.86 |
| 2018-Aug | 32.29 | 33.53 | 31.9 | 33.65 | 31.93 | 33.75 |
| 2018-Sep | 31.2 | 33.58 | 32.08 | 33.72 | 27.9 | 33.74 |
| 2018-Oct | 29.52 | 33.57 | 30.23 | 33.64 | 27.86 | 33.72 |
| 2018-Nov | 31.84 | 33.81 | 28.66 | 33.76 | 27.04 | 33.69 |
| 2018-Dec | 33.25 | 33.54 | 32.95 | 33.7 | 33.15 | 33.55 |
| 2019-Jan | 33.46100000000001 | 33.603 | 33.39 | 33.481 | 33.673 | 33.678 |
| 2019-Feb | 33.426 | 33.6 | 33.347 | 33.399 | 33.344 | 33.39100000000001 |
| 2019-Mar | 30.749 | 33.362 | 31.514 | 33.503 | 32.477 | 33.43 |
| 2019-Apr | 32.837 | 33.382 | 31.942 | 33.465 | 32.04300000000001 | 33.437 |
| 2019-May | 32.354 | 33.69300000000001 | 30.739 | 33.765 | 30.222 | 33.825 |
| 2019-Jun | 29.57 | 33.71100000000001 | 30.92299999999999 | 33.759 | 31.761 | 33.80000000000001 |
| 2019-Jul | 27.02199999999999 | 33.38 | 28.599 | 33.475 | 29.14699999999999 | 33.42400000000001 |
| 2019-Aug | 30.499 | 33.303 | 31.305 | 33.648 | 31.32700000000001 | 33.746 |
| 2019-Sep | 32.843 | 33.39100000000001 | 31.542 | 33.58300000000001 | 29.405 | 33.624 |
| 2019-Oct | 33.245 | 33.872 | 33.05 | 33.911 | 32.578 | 33.89 |
| 2019-Nov | 32.933 | 33.89100000000001 | 31.971 | 33.923 | 31.486 | 33.893 |
| 2019-Dec | 33.579 | 34.06 | 33.41263 | 34.0323 | 33.51900000000001 | 33.63 |
### Chart: pH
| Category | KSt1-1m | KSt1-8m | KSt2-1m | KSt2-10m | KSt3-1m | KSt3-10m |
|---|---|---|---|---|---|---|
| 2015-Jan | 8.138000000000002 | 8.133999999999999 | 8.136999999999999 | 8.133000000000001 | 8.159 | 8.161000000000001 |
| 2015-Feb | 8.102 | 8.105 | 8.116000000000001 | 8.115 | 8.085000000000003 | 8.092 |
| 2015-Mar | 8.197999999999999 | 8.071000000000002 | 8.200000000000001 | 8.030000000000001 | 8.162 | 7.996 |
| 2015-Apr | 8.265 | 8.319 | 8.262 | 8.097999999999997 | 8.202 | 8.014000000000001 |
| 2015-May | 8.18 | 7.978 | 8.209 | 8.146 | 8.184000000000001 | 8.154000000000003 |
| 2015-Jun | 8.036000000000001 | 8.037999999999998 | 8.037999999999998 | 8.06 | 8.043999999999999 | 8.025 |
| 2015-Jul | 8.062 | 7.954 | 8.037999999999998 | 8.012 | 7.987 | 7.984 |
| 2015-Aug | 8.027999999999999 | 7.98 | 7.993 | 8.017999999999997 | 7.999 | 8.006 |
| 2015-Sep | 7.826999999999999 | 7.973 | 8.013000000000003 | 8.056000000000003 | 8.072 | 8.116000000000001 |
| 2015-Oct | 7.953 | 7.918 | 8.033000000000001 | 8.017000000000001 | 8.037999999999998 | 8.037 |
| 2015-Nov | 8.16 | 8.029000000000002 | 8.158000000000001 | 8.077 | 8.076 | 8.084000000000001 |
| 2015-Dec | 8.178 | 8.153 | 8.172 | 8.171000000000001 | 8.172 | 8.162 |
| 2016-Jan | 8.17 | 8.17 | 8.17 | 8.17 | 8.17 | 8.17 |
| 2016-Feb | 8.11 | 8.1 | 8.11 | 8.11 | 8.129999999999997 | 8.120000000000001 |
| 2016-Mar | 8.33 | 8.3 | 8.33 | 8.19 | 8.33 | 8.17 |
| 2016-Apr | 8.219999999999999 | 8.21 | 8.229999999999999 | 8.200000000000001 | 8.229999999999999 | 8.19 |
| 2016-May | 8.229999999999999 | 8.219999999999999 | 8.229999999999999 | 8.27 | 8.25 | 8.219999999999999 |
| 2016-Jun | 8.25 | 8.21 | 8.219999999999999 | 8.26 | 8.229999999999999 | 8.25 |
| 2016-Jul | 8.21 | 8.200000000000001 | 8.16 | 8.16 | 8.21 | 8.19 |
| 2016-Aug | 8.280000000000001 | 8.120000000000001 | 8.219999999999999 | 8.17 | 8.200000000000001 | 8.17 |
| 2016-Sep | 8.27 | 8.09 | 8.21 | 8.15 | 8.17 | 8.16 |
| 2016-Oct | 8.229999999999999 | 8.09 | 8.21 | 8.17 | 8.25 | 8.200000000000001 |
| 2016-Nov | 8.16 | 8.17 | 8.19 | 8.19 | 8.19 | 8.19 |
| 2016-Dec | 8.17 | 8.17 | 8.16 | 8.17 | 8.17 | 8.17 |
| 2017-Jan | 8.19 | 8.18 | 8.21 | 8.21 | 8.26 | 8.229999999999999 |
| 2017-Feb | 8.19 | 8.19 | 8.19 | 8.19 | 8.19 | 8.19 |
| 2017-Mar | 8.290000000000001 | 8.3 | 8.31 | 8.200000000000001 | 8.280000000000001 | 8.219999999999999 |
| 2017-Apr | 8.27 | 8.26 | 8.25 | 8.27 | 8.26 | 8.200000000000001 |
| 2017-May | 8.27 | 8.25 | 8.25 | 8.280000000000001 | 8.27 | 8.25 |
| 2017-Jun | 8.21 | 8.229999999999999 | 8.21 | 8.239999999999998 | 8.229999999999999 | 8.229999999999999 |
| 2017-Jul | 8.35 | 8.18 | 8.25 | 8.26 | 8.35 | 8.26 |
| 2017-Aug | 8.39 | 8.11 | 8.25 | 8.16 | 8.25 | 8.200000000000001 |
| 2017-Sep | 8.219999999999999 | 8.0 | 8.200000000000001 | 8.09 | 8.129999999999997 | 8.16 |
| 2017-Oct | 8.19 | 8.02 | 8.200000000000001 | 8.09 | 8.18 | 8.18 |
| 2017-Nov | 8.21 | 8.16 | 8.239999999999998 | 8.19 | 8.25 | 8.18 |
| 2017-Dec | 8.15 | 8.15 | 8.16 | 8.15 | 8.16 | 8.16 |
| 2018-Jan | 8.139999999999999 | 8.139999999999999 | 8.15 | 8.15 | 8.129999999999997 | 8.129999999999997 |
| 2018-Feb | 8.219999999999999 | 8.229999999999999 | 8.219999999999999 | 8.18 | 8.21 | 8.19 |
| 2018-Mar | 7.9 | 8.120000000000001 | 8.09 | 8.16 | 8.139999999999999 | 8.18 |
| 2018-Apr | 8.19 | 8.239999999999998 | 8.19 | 8.229999999999999 | 8.200000000000001 | 8.19 |
| 2018-May | 8.219999999999999 | 8.18 | 8.21 | 8.18 | 8.200000000000001 | 8.200000000000001 |
| 2018-Jun | 8.35 | 8.120000000000001 | 8.25 | 8.200000000000001 | 8.27 | 8.19 |
| 2018-Jul | 8.26 | 8.17 | 8.239999999999998 | 8.200000000000001 | 8.229999999999999 | 8.21 |
| 2018-Aug | 8.15 | 8.129999999999997 | 8.17 | 8.17 | 8.15 | 8.17 |
| 2018-Sep | 8.229999999999999 | 8.05 | 8.16 | 8.09 | 8.229999999999999 | 8.16 |
| 2018-Oct | 8.219999999999999 | 8.01 | 8.129999999999997 | 8.129999999999997 | 8.129999999999997 | 8.19 |
| 2018-Nov | 7.94 | 8.11 | 7.97 | 8.139999999999999 | 8.02 | 8.16 |
| 2018-Dec | 8.16 | 8.16 | 8.16 | 8.17 | 8.16 | 8.17 |
| 2019-Jan | 8.176 | 8.17 | 8.176 | 8.171000000000001 | 8.16 | 8.162 |
| 2019-Feb | 8.280000000000001 | 8.216000000000001 | 8.305 | 8.181000000000001 | 8.247000000000002 | 8.248 |
| 2019-Mar | 8.212 | 8.216999999999999 | 8.206999999999997 | 8.187000000000001 | 8.200000000000001 | 8.107999999999999 |
| 2019-Apr | 8.337000000000002 | 8.26 | 8.34 | 8.275 | 8.307 | 8.164000000000001 |
| 2019-May | 8.232999999999999 | 8.222 | 8.252 | 8.183000000000002 | 8.223999999999998 | 8.175 |
| 2019-Jun | 8.311000000000003 | 8.193000000000001 | 8.285 | 8.215000000000002 | 8.245 | 8.203000000000001 |
| 2019-Jul | 8.061000000000003 | 8.130999999999998 | 8.233999999999998 | 8.194 | 8.235999999999999 | 8.187000000000001 |
| 2019-Aug | 8.225 | 8.162 | 8.146999999999998 | 8.187999999999999 | 8.154000000000003 | 8.184000000000001 |
| 2019-Sep | 8.216999999999999 | 8.082 | 8.190999999999997 | 8.149 | 8.235999999999999 | 8.179 |
| 2019-Oct | 8.242 | 8.055 | 8.213000000000001 | 8.159 | 8.200999999999999 | 8.194 |
| 2019-Nov | 8.358 | 8.125 | 8.358 | 8.17 | 8.338000000000001 | 8.178 |
| 2019-Dec | 8.117999999999999 | 8.107999999999999 | 8.176 | 8.184919999999998 | 8.189 | 8.187000000000001 |

## Slide 2
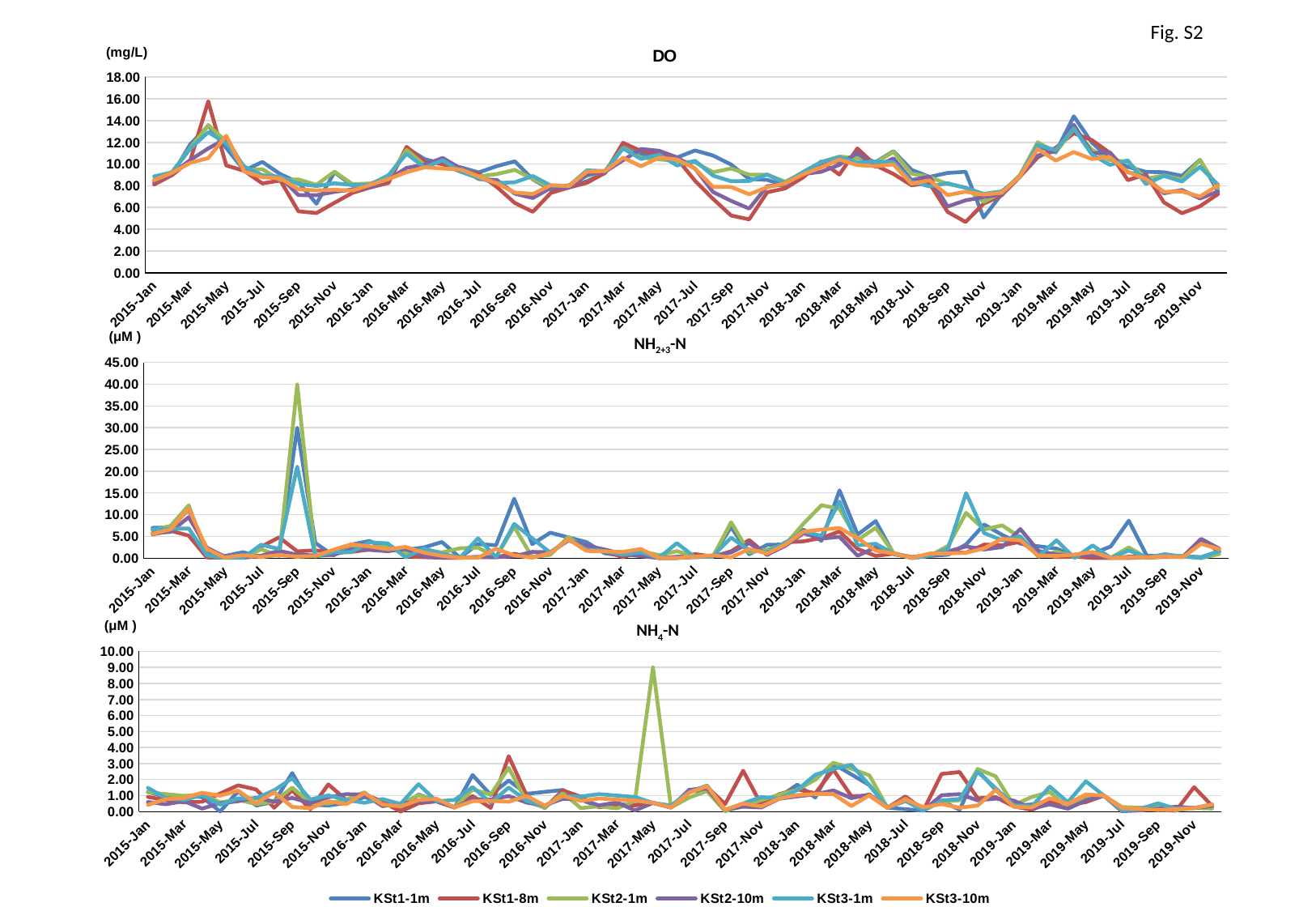

Fig. S2
### Chart: DO
| Category | KSt1-1m | KSt1-8m | KSt2-1m | KSt2-10m | KSt3-1m | KSt3-10m |
|---|---|---|---|---|---|---|
| 2015-Jan | 8.226999999999999 | 8.112 | 8.543000000000001 | 8.404000000000003 | 8.868 | 8.506 |
| 2015-Feb | 9.024000000000001 | 8.986 | 9.289 | 9.109 | 9.235999999999999 | 9.199 |
| 2015-Mar | 11.813 | 10.319 | 11.479 | 10.368 | 11.405 | 10.107 |
| 2015-Apr | 13.531 | 15.767 | 13.592 | 11.438 | 12.935 | 10.549 |
| 2015-May | 11.486 | 9.869 | 12.055 | 12.347 | 11.904 | 12.605 |
| 2015-Jun | 9.402 | 9.374 | 9.446000000000002 | 9.607000000000001 | 9.775 | 9.303 |
| 2015-Jul | 10.194 | 8.215000000000002 | 9.522 | 8.907 | 8.969 | 8.789 |
| 2015-Aug | 9.071000000000002 | 8.485 | 8.521 | 8.775 | 8.838000000000001 | 8.687000000000001 |
| 2015-Sep | 8.304 | 5.649999999999999 | 8.588000000000001 | 7.144999999999999 | 8.171000000000001 | 7.679 |
| 2015-Oct | 6.346999999999999 | 5.492 | 8.087000000000002 | 7.128999999999999 | 7.978 | 7.572 |
| 2015-Nov | 9.247000000000002 | 6.433 | 9.3 | 7.438 | 8.231000000000002 | 7.632 |
| 2015-Dec | 8.005 | 7.346 | 8.148 | 7.660999999999999 | 8.06 | 7.561999999999999 |
| 2016-Jan | 8.19 | 7.85 | 8.21 | 7.85 | 8.1 | 8.09 |
| 2016-Feb | 8.52 | 8.26 | 8.82 | 8.6 | 8.99 | 8.620000000000001 |
| 2016-Mar | 11.49 | 11.58 | 11.31 | 9.65 | 10.95 | 9.25 |
| 2016-Apr | 10.45 | 10.2 | 9.9 | 9.94 | 9.68 | 9.700000000000001 |
| 2016-May | 10.02 | 9.99 | 10.39 | 10.56 | 10.35 | 9.59 |
| 2016-Jun | 9.700000000000001 | 9.3 | 9.51 | 9.59 | 9.290000000000001 | 9.51 |
| 2016-Jul | 9.229999999999999 | 9.120000000000001 | 8.86 | 8.620000000000001 | 8.71 | 8.84 |
| 2016-Aug | 9.81 | 7.89 | 9.08 | 8.530000000000001 | 8.25 | 8.229999999999999 |
| 2016-Sep | 10.25 | 6.42 | 9.460000000000003 | 7.27 | 8.32 | 7.38 |
| 2016-Oct | 8.59 | 5.6 | 8.629999999999997 | 6.87 | 8.9 | 7.24 |
| 2016-Nov | 7.64 | 7.34 | 7.8 | 7.81 | 8.02 | 8.05 |
| 2016-Dec | 7.9 | 7.84 | 8.05 | 7.83 | 7.99 | 7.96 |
| 2017-Jan | 8.42 | 8.27 | 8.91 | 9.02 | 9.42 | 9.3 |
| 2017-Feb | 9.17 | 9.19 | 9.35 | 9.280000000000001 | 9.35 | 9.36 |
| 2017-Mar | 11.55 | 11.95 | 11.5 | 10.35 | 11.43 | 10.58 |
| 2017-Apr | 10.82 | 11.2 | 10.59 | 11.39 | 10.47 | 9.8 |
| 2017-May | 10.9 | 10.97 | 10.44 | 11.22 | 10.87 | 10.59 |
| 2017-Jun | 10.6 | 10.5 | 10.26 | 10.62 | 9.870000000000003 | 10.42 |
| 2017-Jul | 11.25 | 8.45 | 10.15 | 9.57 | 10.3 | 9.57 |
| 2017-Aug | 10.78 | 6.79 | 9.25 | 7.42 | 8.93 | 7.9 |
| 2017-Sep | 9.95 | 5.26 | 9.59 | 6.609999999999999 | 8.42 | 7.89 |
| 2017-Oct | 8.629999999999997 | 4.91 | 9.030000000000001 | 5.9 | 8.44 | 7.21 |
| 2017-Nov | 8.540000000000001 | 7.4 | 9.02 | 7.96 | 9.040000000000001 | 7.87 |
| 2017-Dec | 8.030000000000001 | 7.76 | 8.370000000000003 | 8.1 | 8.239999999999998 | 8.219999999999999 |
| 2018-Jan | 8.99 | 8.780000000000001 | 9.239999999999998 | 9.08 | 9.280000000000001 | 9.06 |
| 2018-Feb | 10.09 | 10.25 | 10.19 | 9.290000000000001 | 10.19 | 9.69 |
| 2018-Mar | 10.21 | 9.030000000000001 | 10.69 | 9.93 | 10.63 | 10.41 |
| 2018-Apr | 10.79 | 11.43 | 10.53 | 11.04 | 10.14 | 9.9 |
| 2018-May | 10.17 | 9.89 | 10.1 | 9.739999999999998 | 10.22 | 9.81 |
| 2018-Jun | 11.18 | 9.09 | 11.13 | 10.52 | 10.18 | 9.960000000000003 |
| 2018-Jul | 9.47 | 8.05 | 9.120000000000001 | 8.52 | 8.370000000000003 | 8.18 |
| 2018-Aug | 8.82 | 8.219999999999999 | 8.82 | 8.85 | 7.94 | 8.5 |
| 2018-Sep | 9.18 | 5.6 | 8.16 | 6.09 | 8.229999999999999 | 7.14 |
| 2018-Oct | 9.290000000000001 | 4.67 | 7.84 | 6.659999999999999 | 7.81 | 7.45 |
| 2018-Nov | 5.08 | 6.35 | 6.52 | 6.95 | 7.26 | 7.189999999999999 |
| 2018-Dec | 7.23 | 7.119999999999999 | 7.359999999999999 | 7.21 | 7.48 | 7.35 |
| 2019-Jan | 8.802 | 8.817 | 8.956000000000003 | 8.895 | 8.837000000000002 | 8.845 |
| 2019-Feb | 11.641 | 10.612 | 12.007 | 10.78 | 11.76 | 11.383 |
| 2019-Mar | 11.056 | 11.492 | 11.08 | 11.191 | 11.273 | 10.319 |
| 2019-Apr | 14.39 | 12.839 | 13.243 | 13.614 | 13.299 | 11.103 |
| 2019-May | 11.989 | 12.205 | 11.32 | 10.991 | 10.883 | 10.459 |
| 2019-Jun | 10.46 | 11.022 | 10.367 | 11.076 | 9.929 | 10.64 |
| 2019-Jul | 9.747000000000002 | 8.515 | 10.216 | 9.253 | 10.332 | 9.279000000000002 |
| 2019-Aug | 9.280000000000001 | 9.040000000000001 | 8.651000000000002 | 8.83 | 8.158000000000001 | 8.625 |
| 2019-Sep | 9.251000000000001 | 6.474 | 8.952 | 7.306999999999999 | 8.914000000000001 | 7.439 |
| 2019-Oct | 8.915 | 5.475 | 8.586 | 7.611999999999999 | 8.393 | 7.473 |
| 2019-Nov | 10.387 | 6.118999999999999 | 10.37 | 6.826999999999999 | 9.747000000000002 | 7.012 |
| 2019-Dec | 7.462 | 7.237 | 7.769255 | 7.472024 | 8.091000000000001 | 8.039 |(mg/L)
### Chart: NOx-N
| Category | KSt1-1m | KSt1-8m | KSt2-1m | KSt2-10m | KSt3-1m | KSt3-10m |
|---|---|---|---|---|---|---|
| 2015-Jan | 6.963999999999999 | 5.467 | 6.401 | 5.705 | 6.633 | 5.581 |
| 2015-Feb | 7.07 | 6.301 | 7.437 | 6.051 | 6.759 | 6.639 |
| 2015-Mar | 11.125 | 5.173999999999999 | 12.148 | 9.428 | 6.817999999999999 | 11.395 |
| 2015-Apr | 1.202 | 0.163 | 0.671 | 2.418 | 0.628 | 2.113 |
| 2015-May | 0.505 | 0.121 | 0.085 | 0.329 | 0.094 | 0.145 |
| 2015-Jun | 1.367 | 0.113 | 0.134 | 0.072 | 0.072 | 0.665 |
| 2015-Jul | 0.328 | 2.618 | 2.035 | 0.563 | 3.083 | 0.272 |
| 2015-Aug | 0.86 | 4.783 | 0.487 | 1.678 | 2.118 | 0.712 |
| 2015-Sep | 29.98699999999999 | 1.553 | 39.915 | 0.781 | 21.038 | 0.319 |
| 2015-Oct | 3.503 | 1.804 | 0.466 | 0.628 | 0.365 | 0.492 |
| 2015-Nov | 0.601 | 1.218 | 0.783 | 0.766 | 1.374 | 1.93 |
| 2015-Dec | 3.13 | 1.423999999999999 | 2.255 | 2.041 | 1.411999999999999 | 3.193 |
| 2016-Jan | 3.95 | 2.0 | 2.47 | 1.9 | 3.64 | 2.69 |
| 2016-Feb | 2.13 | 3.23 | 2.74 | 1.6 | 3.39 | 2.04 |
| 2016-Mar | 1.93 | 0.42 | 1.03 | 2.1 | 0.19 | 2.57 |
| 2016-Apr | 2.45 | 0.23 | 1.91 | 0.48 | 2.23 | 1.27 |
| 2016-May | 3.71 | 0.09 | 1.3 | 0.09 | 1.04 | 0.53 |
| 2016-Jun | 0.13 | 0.09 | 2.27 | 0.09 | 0.09 | 0.09 |
| 2016-Jul | 3.23 | 0.21 | 2.42 | 0.29 | 4.56 | 0.12 |
| 2016-Aug | 2.97 | 0.24 | 0.25 | 0.22 | 0.22 | 2.16 |
| 2016-Sep | 13.67 | 0.99 | 7.21 | 0.45 | 7.859999999999999 | 0.62 |
| 2016-Oct | 3.23 | 0.21 | 0.23 | 1.44 | 4.56 | 0.12 |
| 2016-Nov | 5.859999999999999 | 1.26 | 0.77 | 1.37 | 1.25 | 1.16 |
| 2016-Dec | 4.769999999999999 | 4.22 | 4.9 | 4.06 | 4.33 | 4.34 |
| 2017-Jan | 3.69 | 2.41 | 2.0 | 2.88 | 1.97 | 1.67 |
| 2017-Feb | 1.13 | 1.65 | 1.8 | 1.99 | 1.65 | 1.54 |
| 2017-Mar | 0.57 | 0.24 | 0.88 | 0.98 | 0.77 | 1.42 |
| 2017-Apr | 1.88 | 1.39 | 1.69 | 0.46 | 1.36 | 2.1 |
| 2017-May | 0.04 | 0.04 | 0.67 | 0.04 | 0.04 | 0.04 |
| 2017-Jun | 0.32 | 0.05 | 1.61 | 0.05 | 3.46 | 0.05 |
| 2017-Jul | 0.27 | 0.93 | 0.31 | 0.25 | 0.24 | 0.33 |
| 2017-Aug | 0.4 | 0.37 | 0.33 | 0.43 | 0.52 | 0.66 |
| 2017-Sep | 7.17 | 1.56 | 8.25 | 1.3 | 4.7 | 0.17 |
| 2017-Oct | 0.9 | 4.17 | 1.31 | 3.45 | 1.84 | 2.1 |
| 2017-Nov | 3.06 | 0.7 | 1.97 | 0.82 | 1.45 | 1.07 |
| 2017-Dec | 3.14 | 3.64 | 3.11 | 2.82 | 3.53 | 3.11 |
| 2018-Jan | 6.55 | 3.86 | 7.91 | 5.73 | 6.119999999999999 | 6.08 |
| 2018-Feb | 3.99 | 4.649999999999999 | 12.16 | 4.49 | 5.149999999999999 | 6.57 |
| 2018-Mar | 15.59 | 6.13 | 11.32 | 4.9 | 13.01 | 6.93 |
| 2018-Apr | 5.41 | 2.12 | 4.18 | 0.56 | 2.96 | 4.58 |
| 2018-May | 8.52 | 0.52 | 6.98 | 2.49 | 3.3 | 1.73 |
| 2018-Jun | 0.88 | 1.03 | 1.1 | 1.07 | 1.03 | 1.06 |
| 2018-Jul | 0.16 | 0.25 | 0.18 | 0.09 | 0.02 | 0.07 |
| 2018-Aug | 0.51 | 0.76 | 0.55 | 0.5 | 0.56 | 1.07 |
| 2018-Sep | 0.79 | 1.45 | 2.82 | 1.52 | 2.15 | 1.08 |
| 2018-Oct | 3.16 | 1.21 | 10.41 | 2.7 | 14.97 | 1.23 |
| 2018-Nov | 7.74 | 3.12 | 6.59 | 1.99 | 5.77 | 2.25 |
| 2018-Dec | 5.34 | 2.93 | 7.54 | 2.54 | 4.1 | 4.44 |
| 2019-Jan | 3.341665718915619 | 3.790956400420641 | 4.770913133845974 | 6.704687845351122 | 5.023423374164932 | 4.104621464566847 |
| 2019-Feb | 2.732566267616875 | 0.952860771111759 | 0.360962097112171 | 1.667048417554955 | 0.417094740201039 | 0.496783046014701 |
| 2019-Mar | 2.195296683766274 | 0.258720497200302 | 1.442016660886187 | 0.82305403396875 | 4.106813654667572 | 0.516329234233147 |
| 2019-Apr | 0.507307916593864 | 0.344423014773486 | 0.389028418656605 | 0.70026387721185 | 0.0447147954239902 | 0.815536269269348 |
| 2019-May | 1.041 | 0.045 | 1.085 | 0.7 | 2.929 | 1.411999999999999 |
| 2019-Jun | 2.691 | 0.059 | 0.059 | 0.059 | 0.059 | 0.059 |
| 2019-Jul | 8.610407400000001 | 0.3206946 | 2.47101 | 0.046924 | 1.7661415 | 0.17423512 |
| 2019-Aug | 0.5271 | 0.3202 | 0.075918 | 0.216173 | 0.1342 | 0.2157 |
| 2019-Sep | 0.5062 | 0.5135 | 0.312347 | 0.2847971 | 0.9106 | 0.375 |
| 2019-Oct | 0.4866 | 0.3568 | 0.5207251 | 0.307338 | 0.3411 | 0.213 |
| 2019-Nov | 0.222 | 4.336 | 0.042 | 4.424999999999999 | 0.049 | 3.293 |
| 2019-Dec | 1.5943 | 1.4403 | 0.9255 | 2.1887 | 1.632 | 2.0884 |(μM )
NH2+3-N
### Chart: NH4-N
| Category | KSt1-1m | KSt1-8m | KSt2-1m | KSt2-10m | KSt3-1m | KSt3-10m |
|---|---|---|---|---|---|---|
| 2015-Jan | 1.229 | 0.918 | 1.202 | 0.599 | 1.477 | 0.42 |
| 2015-Feb | 0.918 | 0.718 | 1.089 | 0.457 | 0.845 | 0.781 |
| 2015-Mar | 0.974 | 0.576 | 0.975 | 0.658 | 0.731 | 0.844 |
| 2015-Apr | 0.908 | 0.616 | 1.078 | 0.185 | 1.028 | 1.175 |
| 2015-May | 0.006 | 1.125 | 0.577 | 0.521 | 0.437 | 0.997 |
| 2015-Jun | 1.263 | 1.638 | 0.734 | 0.647 | 0.823 | 1.309 |
| 2015-Jul | 0.376 | 1.378 | 0.47 | 0.863 | 0.822 | 0.524 |
| 2015-Aug | 0.608 | 0.24 | 0.619 | 0.625 | 1.332 | 1.205 |
| 2015-Sep | 2.405 | 1.338 | 1.494 | 0.851 | 2.077 | 0.29 |
| 2015-Oct | 0.437 | 0.137 | 0.545 | 0.537 | 0.758 | 0.213 |
| 2015-Nov | 0.38 | 1.698 | 0.503 | 0.895 | 1.011 | 0.644 |
| 2015-Dec | 0.539 | 0.718 | 0.752 | 1.088 | 0.732 | 0.467 |
| 2016-Jan | 0.9 | 1.09 | 1.2 | 1.06 | 0.55 | 1.15 |
| 2016-Feb | 0.64 | 0.62 | 0.34 | 0.35 | 0.78 | 0.45 |
| 2016-Mar | 0.07 | 0.0 | 0.47 | 0.51 | 0.48 | 0.32 |
| 2016-Apr | 1.07 | 0.52 | 1.03 | 0.62 | 1.71 | 0.72 |
| 2016-May | 0.64 | 0.64 | 0.64 | 0.64 | 0.64 | 0.8 |
| 2016-Jun | 0.26 | 0.26 | 0.28 | 0.26 | 0.73 | 0.26 |
| 2016-Jul | 2.28 | 0.97 | 1.38 | 0.78 | 1.53 | 0.65 |
| 2016-Aug | 1.04 | 0.23 | 1.07 | 0.75 | 0.55 | 0.67 |
| 2016-Sep | 1.94 | 3.46 | 2.73 | 0.97 | 1.5 | 0.62 |
| 2016-Oct | 1.11 | 1.0 | 0.62 | 0.56 | 0.67 | 0.97 |
| 2016-Nov | 1.23 | 0.22 | 0.24 | 0.39 | 0.34 | 0.37 |
| 2016-Dec | 1.35 | 1.32 | 1.16 | 0.8 | 0.95 | 0.98 |
| 2017-Jan | 0.74 | 0.93 | 0.22 | 0.76 | 0.94 | 0.67 |
| 2017-Feb | 0.31 | 0.27 | 0.33 | 0.39 | 1.09 | 0.82 |
| 2017-Mar | 0.46 | 0.42 | 0.2 | 0.57 | 1.0 | 0.75 |
| 2017-Apr | 0.42 | 0.39 | 0.67 | 0.07 | 0.91 | 0.69 |
| 2017-May | 0.54 | 0.54 | 9.0 | 0.54 | 0.54 | 0.54 |
| 2017-Jun | 0.26 | 0.26 | 0.26 | 0.26 | 0.38 | 0.26 |
| 2017-Jul | 1.28 | 1.33 | 0.87 | 1.36 | 1.21 | 1.17 |
| 2017-Aug | 1.31 | 1.53 | 1.28 | 1.44 | 1.63 | 1.59 |
| 2017-Sep | 0.07 | 0.47 | 0.02 | 0.12 | 0.1 | 0.12 |
| 2017-Oct | 0.34 | 2.55 | 0.37 | 0.31 | 0.51 | 0.5 |
| 2017-Nov | 0.53 | 0.44 | 0.71 | 0.26 | 0.91 | 0.33 |
| 2017-Dec | 0.91 | 1.09 | 1.06 | 0.8 | 0.82 | 0.82 |
| 2018-Jan | 1.68 | 1.39 | 1.36 | 0.95 | 1.34 | 1.03 |
| 2018-Feb | 0.88 | 1.14 | 1.99 | 1.08 | 2.3 | 1.1 |
| 2018-Mar | 2.98 | 2.62 | 3.05 | 1.32 | 2.66 | 1.1 |
| 2018-Apr | 2.319999999999999 | 0.96 | 2.68 | 0.84 | 2.92 | 0.35 |
| 2018-May | 1.65 | 1.0 | 2.26 | 1.07 | 1.65 | 1.02 |
| 2018-Jun | 0.24 | 0.24 | 0.24 | 0.24 | 0.24 | 0.24 |
| 2018-Jul | 0.15 | 0.95 | 0.81 | 0.77 | 0.66 | 0.75 |
| 2018-Aug | 0.06 | 0.09 | 0.11 | 0.07 | 0.07 | 0.27 |
| 2018-Sep | 0.68 | 2.35 | 0.62 | 1.02 | 0.69 | 0.47 |
| 2018-Oct | 0.11 | 2.47 | 0.71 | 1.09 | 0.79 | 0.24 |
| 2018-Nov | 2.56 | 0.83 | 2.66 | 0.7 | 2.48 | 0.38 |
| 2018-Dec | 1.36 | 0.9 | 2.2 | 0.81 | 1.46 | 1.35 |
| 2019-Jan | 0.360708589080648 | 0.32928124307002 | 0.400168933847356 | 0.687947591038846 | 0.390585707261156 | 0.296021809623796 |
| 2019-Feb | 0.444757075703222 | 0.108954530231859 | 0.91030486841385 | 0.19382770106528 | 0.327558297195854 | 0.261726237732017 |
| 2019-Mar | 0.684616036754195 | 0.584391892413512 | 1.2276567245736 | 0.451104112826417 | 1.554898150204373 | 0.83163112918913 |
| 2019-Apr | 0.327115480652375 | 0.403132320616222 | 0.24357075944939 | 0.181724048876862 | 0.586163158587426 | 0.481363243297463 |
| 2019-May | 0.92 | 0.593 | 0.947 | 0.699 | 1.892 | 1.072 |
| 2019-Jun | 0.998 | 0.998 | 0.998 | 0.998 | 0.998 | 0.998 |
| 2019-Jul | 0.1801775 | 0.0195175 | 0.285029 | 0.029383 | 0.0730709 | 0.22908015 |
| 2019-Aug | 0.1253 | 0.2097 | 0.243813 | 0.075544 | 0.1624 | 0.1597 |
| 2019-Sep | 0.1460397 | 0.1188958 | 0.3414484 | 0.1765074 | 0.518161 | 0.10352343 |
| 2019-Oct | 0.2363349 | 0.0560215 | 0.0873151 | 0.3075185 | 0.1753 | 0.1538 |
| 2019-Nov | 0.222 | 1.523 | 0.222 | 0.222 | 0.222 | 0.222 |
| 2019-Dec | 0.3851 | 0.3537 | 0.1888 | 0.3534 | 0.4476 | 0.4389 |(μM )
NH4-N

## Slide 3
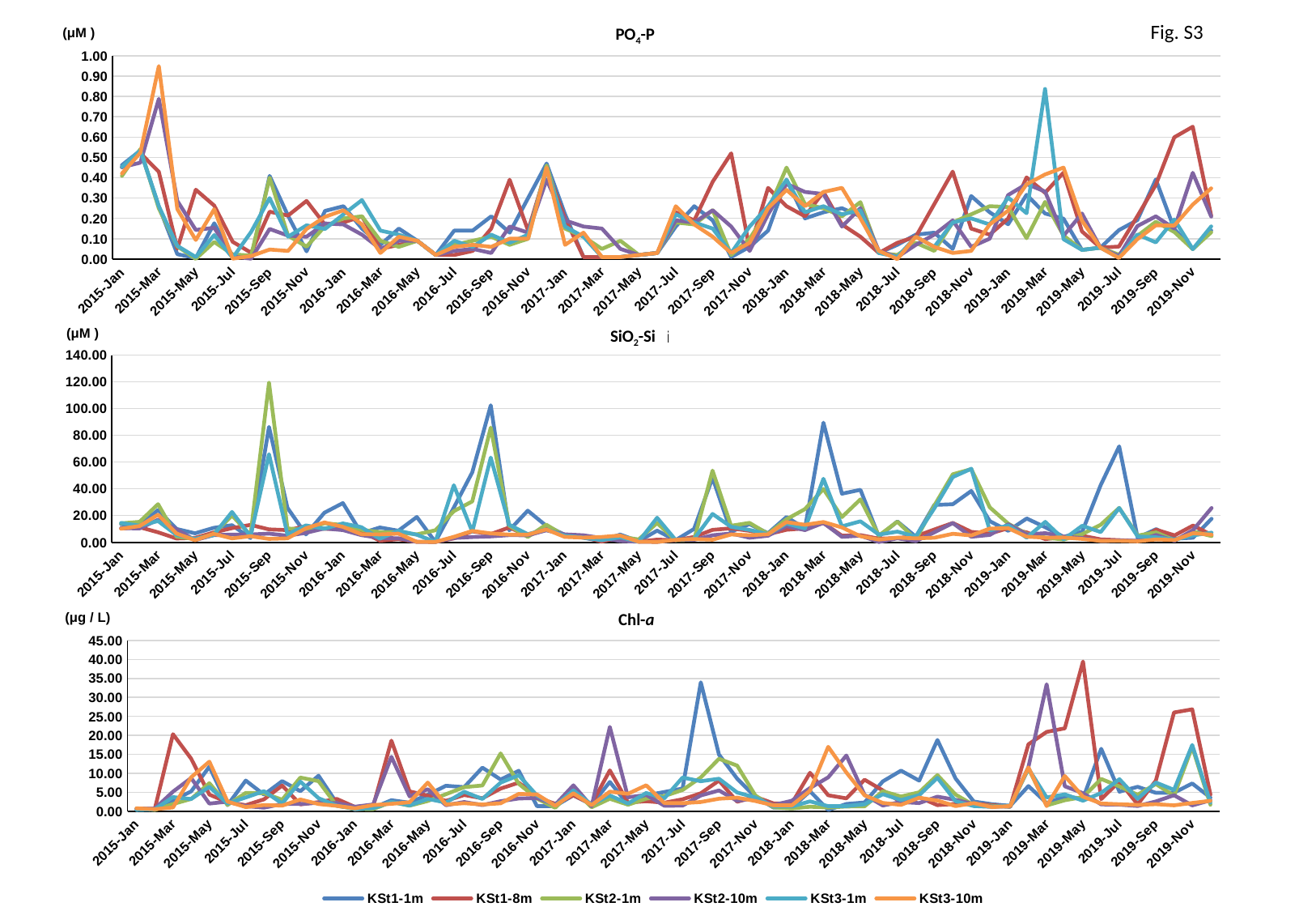

### Chart: PO4-P
| Category | KSt1-1m | KSt1-8m | KSt2-1m | KSt2-10m | KSt3-1m | KSt3-10m |
|---|---|---|---|---|---|---|
| 2015-Jan | 0.462 | 0.451 | 0.409 | 0.452 | 0.453 | 0.421 |
| 2015-Feb | 0.534 | 0.521 | 0.541 | 0.474 | 0.534 | 0.523 |
| 2015-Mar | 0.259 | 0.429 | 0.249 | 0.788 | 0.26 | 0.949 |
| 2015-Apr | 0.024 | 0.051 | 0.063 | 0.286 | 0.065 | 0.246 |
| 2015-May | 0.005 | 0.341 | 0.001 | 0.143 | 0.01 | 0.094 |
| 2015-Jun | 0.176 | 0.263 | 0.084 | 0.154 | 0.118 | 0.245 |
| 2015-Jul | 0.015 | 0.085 | 0.023 | 0.012 | 0.006 | 0.002 |
| 2015-Aug | 0.006 | 0.031 | 0.017 | 0.002 | 0.134 | 0.015 |
| 2015-Sep | 0.409 | 0.233 | 0.4 | 0.148 | 0.3 | 0.047 |
| 2015-Oct | 0.213 | 0.214 | 0.115 | 0.115 | 0.11 | 0.04 |
| 2015-Nov | 0.038 | 0.286 | 0.061 | 0.11 | 0.166 | 0.148 |
| 2015-Dec | 0.237 | 0.169 | 0.162 | 0.175 | 0.148 | 0.208 |
| 2016-Jan | 0.26 | 0.18 | 0.2 | 0.17 | 0.22 | 0.24 |
| 2016-Feb | 0.15 | 0.21 | 0.21 | 0.12 | 0.29 | 0.17 |
| 2016-Mar | 0.07 | 0.05 | 0.09 | 0.05 | 0.14 | 0.03 |
| 2016-Apr | 0.15 | 0.07 | 0.06 | 0.09 | 0.12 | 0.11 |
| 2016-May | 0.09 | 0.09 | 0.09 | 0.09 | 0.09 | 0.09 |
| 2016-Jun | 0.02 | 0.02 | 0.02 | 0.02 | 0.02 | 0.02 |
| 2016-Jul | 0.14 | 0.02 | 0.07 | 0.04 | 0.09 | 0.06 |
| 2016-Aug | 0.14 | 0.04 | 0.09 | 0.05 | 0.06 | 0.07 |
| 2016-Sep | 0.21 | 0.15 | 0.11 | 0.03 | 0.12 | 0.06 |
| 2016-Oct | 0.13 | 0.39 | 0.07 | 0.16 | 0.08 | 0.1 |
| 2016-Nov | 0.3 | 0.14 | 0.1 | 0.13 | 0.12 | 0.1 |
| 2016-Dec | 0.47 | 0.44 | 0.46 | 0.39 | 0.44 | 0.45 |
| 2017-Jan | 0.22 | 0.2 | 0.15 | 0.19 | 0.16 | 0.07 |
| 2017-Feb | 0.01 | 0.01 | 0.11 | 0.16 | 0.11 | 0.13 |
| 2017-Mar | 0.01 | 0.01 | 0.05 | 0.15 | 0.01 | 0.01 |
| 2017-Apr | 0.01 | 0.01 | 0.09 | 0.05 | 0.01 | 0.01 |
| 2017-May | 0.02 | 0.02 | 0.02 | 0.02 | 0.02 | 0.02 |
| 2017-Jun | 0.03 | 0.03 | 0.03 | 0.03 | 0.03 | 0.03 |
| 2017-Jul | 0.16 | 0.23 | 0.18 | 0.19 | 0.22 | 0.26 |
| 2017-Aug | 0.26 | 0.19 | 0.17 | 0.18 | 0.18 | 0.17 |
| 2017-Sep | 0.19 | 0.38 | 0.24 | 0.24 | 0.15 | 0.11 |
| 2017-Oct | 0.01 | 0.52 | 0.02 | 0.16 | 0.03 | 0.03 |
| 2017-Nov | 0.06 | 0.05 | 0.1 | 0.04 | 0.16 | 0.08 |
| 2017-Dec | 0.14 | 0.35 | 0.24 | 0.22 | 0.26 | 0.25 |
| 2018-Jan | 0.39 | 0.26 | 0.45 | 0.37 | 0.39 | 0.34 |
| 2018-Feb | 0.2 | 0.21 | 0.27 | 0.33 | 0.23 | 0.26 |
| 2018-Mar | 0.23 | 0.33 | 0.25 | 0.32 | 0.26 | 0.33 |
| 2018-Apr | 0.25 | 0.17 | 0.21 | 0.16 | 0.22 | 0.35 |
| 2018-May | 0.21 | 0.11 | 0.28 | 0.25 | 0.24 | 0.2 |
| 2018-Jun | 0.03 | 0.03 | 0.03 | 0.04 | 0.03 | 0.04 |
| 2018-Jul | 0.07 | 0.08 | 0.02 | 0.01 | 0.01 | 0.0 |
| 2018-Aug | 0.12 | 0.11 | 0.08 | 0.07 | 0.11 | 0.11 |
| 2018-Sep | 0.13 | 0.27 | 0.04 | 0.12 | 0.06 | 0.06 |
| 2018-Oct | 0.05 | 0.43 | 0.18 | 0.19 | 0.18 | 0.03 |
| 2018-Nov | 0.31 | 0.15 | 0.22 | 0.06 | 0.2 | 0.04 |
| 2018-Dec | 0.23 | 0.12 | 0.26 | 0.1 | 0.17 | 0.17 |
| 2019-Jan | 0.171128876231102 | 0.199301220521541 | 0.256012975477537 | 0.314835362090472 | 0.302813938305301 | 0.238026723096671 |
| 2019-Feb | 0.315907740834383 | 0.401535551884088 | 0.102650401119807 | 0.368932008652425 | 0.225290775482325 | 0.367539686521536 |
| 2019-Mar | 0.224130507039917 | 0.326118103127573 | 0.280983660717905 | 0.332151499028094 | 0.8367522446313 | 0.416154934258428 |
| 2019-Apr | 0.198604601306942 | 0.425089001264969 | 0.115761434519017 | 0.11471719292085 | 0.0977772736616938 | 0.450034772776739 |
| 2019-May | 0.045 | 0.136 | 0.045 | 0.224 | 0.045 | 0.189 |
| 2019-Jun | 0.056 | 0.056 | 0.056 | 0.056 | 0.056 | 0.056 |
| 2019-Jul | 0.1423551 | 0.061491 | 0.021344 | 0.01451 | 0.0067275 | 0.0053987 |
| 2019-Aug | 0.1914777 | 0.2107537 | 0.113628 | 0.162145 | 0.120985 | 0.09779787 |
| 2019-Sep | 0.3915948 | 0.3647759 | 0.183283 | 0.210195 | 0.0821541 | 0.16568312 |
| 2019-Oct | 0.147897 | 0.5990685 | 0.1330908 | 0.1500388 | 0.19483 | 0.16503127 |
| 2019-Nov | 0.049 | 0.651 | 0.049 | 0.424 | 0.049 | 0.268 |
| 2019-Dec | 0.1408 | 0.2129 | 0.1315 | 0.2089 | 0.1606 | 0.3479 |Fig. S3
PO4-P
(μM )
### Chart: SiO2-Si
| Category | KSt1-1m | KSt1-8m | KSt2-1m | KSt2-10m | KSt3-1m | KSt3-10m |
|---|---|---|---|---|---|---|
| 2015-Jan | 13.551 | 10.305 | 14.21 | 10.604 | 14.492 | 10.178 |
| 2015-Feb | 12.921 | 11.294 | 15.326 | 10.386 | 12.431 | 12.16 |
| 2015-Mar | 24.148 | 7.477 | 28.567 | 16.887 | 15.924 | 20.674 |
| 2015-Apr | 9.981000000000003 | 2.86 | 4.291 | 9.752 | 5.46 | 6.556999999999999 |
| 2015-May | 6.724999999999999 | 3.315999999999999 | 2.411 | 1.647 | 2.66 | 1.557 |
| 2015-Jun | 10.794 | 7.298 | 6.101999999999999 | 5.347999999999999 | 5.343999999999999 | 6.365999999999999 |
| 2015-Jul | 12.805 | 10.589 | 20.065 | 5.796 | 22.7 | 2.963 |
| 2015-Aug | 3.154 | 13.039 | 5.293 | 5.860999999999999 | 4.526999999999999 | 4.561999999999999 |
| 2015-Sep | 86.34 | 9.677000000000001 | 119.319 | 6.531 | 65.88599999999998 | 2.572 |
| 2015-Oct | 25.815 | 9.139999999999999 | 10.382 | 4.918 | 5.873 | 2.934 |
| 2015-Nov | 5.924999999999999 | 11.998 | 9.282 | 7.115999999999999 | 12.647 | 10.509 |
| 2015-Dec | 22.157 | 10.04 | 14.277 | 10.338 | 10.163 | 14.869 |
| 2016-Jan | 29.35 | 10.27 | 12.98 | 9.139999999999999 | 14.15 | 11.57 |
| 2016-Feb | 6.77 | 8.120000000000001 | 9.33 | 5.37 | 11.32 | 6.05 |
| 2016-Mar | 11.11 | 1.2 | 7.659999999999999 | 3.24 | 2.71 | 5.92 |
| 2016-Apr | 8.68 | 2.35 | 7.47 | 3.05 | 8.629999999999997 | 6.48 |
| 2016-May | 18.93 | 0.39 | 5.97 | 0.39 | 5.67 | 0.39 |
| 2016-Jun | 0.32 | 0.32 | 9.01 | 0.32 | 0.32 | 0.32 |
| 2016-Jul | 25.97 | 2.92 | 23.29 | 3.23 | 42.68 | 4.09 |
| 2016-Aug | 52.34 | 8.200000000000001 | 30.6 | 3.95 | 7.94 | 8.52 |
| 2016-Sep | 102.49 | 6.18 | 85.8 | 4.42 | 63.38 | 6.659999999999999 |
| 2016-Oct | 8.99 | 11.1 | 12.35 | 5.38 | 13.09 | 5.73 |
| 2016-Nov | 23.68 | 4.92 | 4.03 | 5.26 | 6.1 | 5.74 |
| 2016-Dec | 12.36 | 9.91 | 13.23 | 8.97 | 9.65 | 9.65 |
| 2017-Jan | 5.68 | 5.18 | 4.859999999999999 | 5.95 | 4.94 | 4.07 |
| 2017-Feb | 3.4 | 3.97 | 3.66 | 5.26 | 3.55 | 3.52 |
| 2017-Mar | 1.0 | 0.56 | 2.33 | 3.22 | 1.61 | 4.0 |
| 2017-Apr | 5.8 | 3.45 | 4.22 | 1.0 | 3.77 | 5.08 |
| 2017-May | 0.8 | 0.47 | 2.12 | 0.47 | 0.47 | 0.47 |
| 2017-Jun | 8.75 | 2.03 | 14.43 | 0.27 | 18.35 | 0.27 |
| 2017-Jul | 1.55 | 1.53 | 1.51 | 1.81 | 1.87 | 1.85 |
| 2017-Aug | 9.98 | 4.02 | 1.87 | 1.85 | 2.5 | 2.46 |
| 2017-Sep | 48.26 | 9.32 | 53.62 | 5.28 | 21.23 | 1.94 |
| 2017-Oct | 7.96 | 10.47 | 12.39 | 6.619999999999999 | 11.6 | 5.859999999999999 |
| 2017-Nov | 13.62 | 8.61 | 14.52 | 3.45 | 9.290000000000001 | 5.31 |
| 2017-Dec | 6.79 | 6.05 | 6.31 | 4.93 | 6.119999999999999 | 6.02 |
| 2018-Jan | 18.89 | 9.39 | 17.21 | 12.11 | 14.0 | 15.13 |
| 2018-Feb | 10.34 | 10.44 | 24.82 | 9.120000000000001 | 10.42 | 13.1 |
| 2018-Mar | 89.5 | 14.43 | 39.88 | 14.58 | 47.51 | 15.18 |
| 2018-Apr | 36.36 | 4.94 | 18.85 | 4.1 | 11.96 | 11.14 |
| 2018-May | 39.28 | 5.38 | 32.13 | 4.97 | 15.73 | 4.57 |
| 2018-Jun | 4.33 | 2.69 | 5.07 | 0.23 | 5.79 | 2.07 |
| 2018-Jul | 15.56 | 3.2 | 15.48 | 2.97 | 7.98 | 3.49 |
| 2018-Aug | 4.149999999999999 | 4.47 | 2.04 | 0.86 | 4.35 | 2.97 |
| 2018-Sep | 27.94 | 9.59 | 27.52 | 7.46 | 25.39 | 3.41 |
| 2018-Oct | 28.48 | 14.48 | 50.89 | 14.39 | 48.78 | 6.39 |
| 2018-Nov | 38.43 | 7.83 | 54.73 | 4.159999999999999 | 55.03 | 5.14 |
| 2018-Dec | 15.83 | 6.53 | 26.14 | 5.54 | 8.88 | 10.33 |
| 2019-Jan | 8.602664877752668 | 10.57300460038599 | 13.93771798125268 | 13.51712880864435 | 12.67996883131884 | 10.53416037743808 |
| 2019-Feb | 17.84309035949211 | 7.383644621153265 | 6.343946564349576 | 5.91363094671387 | 3.4857 | 4.21068 |
| 2019-Mar | 11.27586 | 2.38741 | 3.57148 | 6.842177999999999 | 15.27483 | 3.51758 |
| 2019-Apr | 4.10687 | 3.814756 | 2.017479999999999 | 4.278574 | 2.203578 | 3.578409999999999 |
| 2019-May | 7.979 | 4.924999999999999 | 6.147999999999999 | 3.222 | 12.394 | 2.562 |
| 2019-Jun | 42.78400000000001 | 2.205 | 13.141 | 0.981 | 7.657999999999999 | 1.065 |
| 2019-Jul | 71.799826 | 1.6344769 | 25.72978 | 1.308235 | 25.694145 | 1.14374307 |
| 2019-Aug | 3.447321 | 0.9542901 | 5.056 | 1.226614 | 4.4102823 | 0.77882336 |
| 2019-Sep | 9.8694024 | 9.456704700000003 | 7.4142723 | 5.4701012 | 3.7521297 | 2.161501 |
| 2019-Oct | 2.7215907 | 5.2061993 | 1.3796214 | 1.1690613 | 1.9705932 | 1.68844276 |
| 2019-Nov | 3.429 | 12.578 | 8.040999999999999 | 9.167000000000002 | 5.212 | 6.8 |
| 2019-Dec | 17.595 | 5.556 | 4.677 | 25.655 | 7.227999999999999 | 5.701 |(μM )
SiO2-Si
### Chart: Chl-a
| Category | KSt1-1m | KSt1-8m | KSt2-1m | KSt2-10m | KSt3-1m | KSt3-10m |
|---|---|---|---|---|---|---|
| 2015-Jan | 0.564 | 0.709 | 0.753 | 0.648 | 0.375 | 0.8 |
| 2015-Feb | 0.673 | 0.613 | 0.373 | 0.716 | 0.541 | 0.576 |
| 2015-Mar | 2.858999999999999 | 20.318 | 1.927999999999999 | 5.172 | 3.752 | 1.029 |
| 2015-Apr | 5.26 | 13.875 | 3.271 | 8.783000000000001 | 3.245 | 8.963000000000003 |
| 2015-May | 11.752 | 4.417 | 7.419 | 2.048 | 6.458 | 13.086 |
| 2015-Jun | 1.641 | 2.389 | 1.713 | 2.555 | 2.058 | 2.576 |
| 2015-Jul | 8.110999999999999 | 1.625 | 4.847999999999999 | 1.39 | 3.653 | 1.132 |
| 2015-Aug | 4.4 | 3.164 | 4.758 | 0.963 | 5.311999999999999 | 1.672 |
| 2015-Sep | 7.961 | 6.793 | 3.063 | 1.937999999999999 | 2.078 | 1.514 |
| 2015-Oct | 5.312999999999999 | 1.835 | 8.886000000000003 | 1.933 | 7.828999999999999 | 3.124 |
| 2015-Nov | 9.387 | 2.416999999999999 | 7.953 | 2.254 | 3.461 | 1.925999999999999 |
| 2015-Dec | 2.185 | 3.277 | 1.461 | 2.179 | 1.504 | 1.452 |
| 2016-Jan | 0.46 | 1.16 | 0.62 | 1.23 | 0.69 | 0.8 |
| 2016-Feb | 0.93 | 1.36 | 0.57 | 1.82 | 0.82 | 1.66 |
| 2016-Mar | 2.94 | 18.63 | 2.47 | 14.35 | 2.5 | 1.98 |
| 2016-Apr | 2.27 | 5.26 | 1.49 | 3.96 | 1.56 | 2.4 |
| 2016-May | 4.39 | 4.149999999999999 | 2.73 | 5.819999999999999 | 3.26 | 7.57 |
| 2016-Jun | 6.74 | 2.76 | 4.53 | 1.62 | 2.319999999999999 | 1.81 |
| 2016-Jul | 6.38 | 4.31 | 6.37 | 2.46 | 5.149999999999999 | 2.15 |
| 2016-Aug | 11.51 | 3.44 | 6.819999999999999 | 1.65 | 3.26 | 1.81 |
| 2016-Sep | 8.38 | 6.0 | 15.24 | 2.68 | 7.56 | 2.12 |
| 2016-Oct | 10.68 | 7.56 | 7.26 | 3.39 | 9.5 | 4.6 |
| 2016-Nov | 1.37 | 3.64 | 3.37 | 3.51 | 4.17 | 4.45 |
| 2016-Dec | 1.39 | 2.0 | 0.92 | 1.73 | 1.48 | 1.48 |
| 2017-Jan | 4.22 | 4.21 | 5.72 | 6.85 | 5.53 | 4.75 |
| 2017-Feb | 1.78 | 1.93 | 1.07 | 1.34 | 1.77 | 1.65 |
| 2017-Mar | 7.75 | 10.78 | 3.31 | 22.23 | 4.18 | 5.189999999999999 |
| 2017-Apr | 1.76 | 2.31 | 1.76 | 3.69 | 2.06 | 4.619999999999999 |
| 2017-May | 4.21 | 2.67 | 3.33 | 4.33 | 4.84 | 6.859999999999999 |
| 2017-Jun | 5.1 | 2.27 | 4.05 | 1.48 | 3.43 | 2.2 |
| 2017-Jul | 6.01 | 3.18 | 5.619999999999999 | 1.52 | 8.9 | 2.16 |
| 2017-Aug | 33.97 | 4.75 | 8.93 | 4.18 | 7.94 | 2.46 |
| 2017-Sep | 14.93 | 8.11 | 13.84 | 5.51 | 8.59 | 3.33 |
| 2017-Oct | 8.61 | 2.53 | 12.04 | 2.76 | 4.96 | 3.61 |
| 2017-Nov | 3.81 | 3.82 | 4.18 | 3.5 | 3.65 | 2.72 |
| 2017-Dec | 0.99 | 2.1 | 1.05 | 1.66 | 1.29 | 1.58 |
| 2018-Jan | 0.99 | 2.1 | 0.76 | 2.8 | 1.29 | 1.58 |
| 2018-Feb | 5.3 | 10.15 | 1.22 | 6.13 | 2.64 | 5.43 |
| 2018-Mar | 0.37 | 4.23 | 1.0 | 8.95 | 1.36 | 17.0 |
| 2018-Apr | 1.94 | 3.42 | 1.28 | 14.71 | 1.38 | 10.23 |
| 2018-May | 2.35 | 8.32 | 1.33 | 4.319999999999999 | 1.87 | 4.22 |
| 2018-Jun | 7.88 | 5.52 | 5.3 | 1.54 | 4.52 | 2.22 |
| 2018-Jul | 10.71 | 2.44 | 3.91 | 2.46 | 2.97 | 1.7 |
| 2018-Aug | 8.08 | 3.69 | 5.02 | 2.18 | 4.27 | 3.59 |
| 2018-Sep | 18.8 | 1.64 | 9.540000000000001 | 3.87 | 8.83 | 2.84 |
| 2018-Oct | 8.639999999999999 | 1.96 | 4.41 | 3.04 | 2.68 | 1.39 |
| 2018-Nov | 2.59 | 2.0 | 1.64 | 1.89 | 1.39 | 2.17 |
| 2018-Dec | 1.88 | 1.65 | 1.47 | 1.28 | 1.18 | 1.11 |
| 2019-Jan | 1.467222756752635 | 1.230238018146694 | 1.201212254792419 | 1.270856388206488 | 1.546335660529226 | 1.412887943644328 |
| 2019-Feb | 6.660976040367743 | 17.65089993697061 | 11.30717776680427 | 11.6579647361224 | 11.27425830348783 | 11.56504689611634 |
| 2019-Mar | 2.201583418933889 | 20.92209461767931 | 1.556556622929893 | 33.45795013903066 | 3.668844605810588 | 1.389923963185686 |
| 2019-Apr | 3.861848233480324 | 21.86538768010277 | 2.932006134562504 | 6.596066292134937 | 4.47152200369154 | 9.305462013530788 |
| 2019-May | 3.374 | 39.438 | 3.767 | 4.792 | 2.805 | 3.86 |
| 2019-Jun | 16.472 | 3.222 | 8.530000000000001 | 1.832 | 4.855999999999999 | 2.074 |
| 2019-Jul | 5.177564799999999 | 7.8960311 | 6.571598 | 1.768719 | 8.459290200000003 | 1.86526508 |
| 2019-Aug | 6.4568223 | 1.8177885 | 4.367497999999999 | 1.410233 | 3.2991643 | 1.70858791 |
| 2019-Sep | 4.8894311 | 8.0873534 | 7.2008287 | 2.612147499999999 | 7.597853299999999 | 1.89362715 |
| 2019-Oct | 4.900935199999999 | 26.026072 | 4.108788499999999 | 4.247589999999999 | 5.660250899999999 | 1.59350253 |
| 2019-Nov | 7.338 | 26.864 | 16.982 | 1.569 | 17.482 | 2.212 |
| 2019-Dec | 3.616412399999999 | 4.4064353 | 1.7391181 | 2.911873899999999 | 2.2962711 | 2.77851471 |Chl-a
(μg / L)

## Slide 4
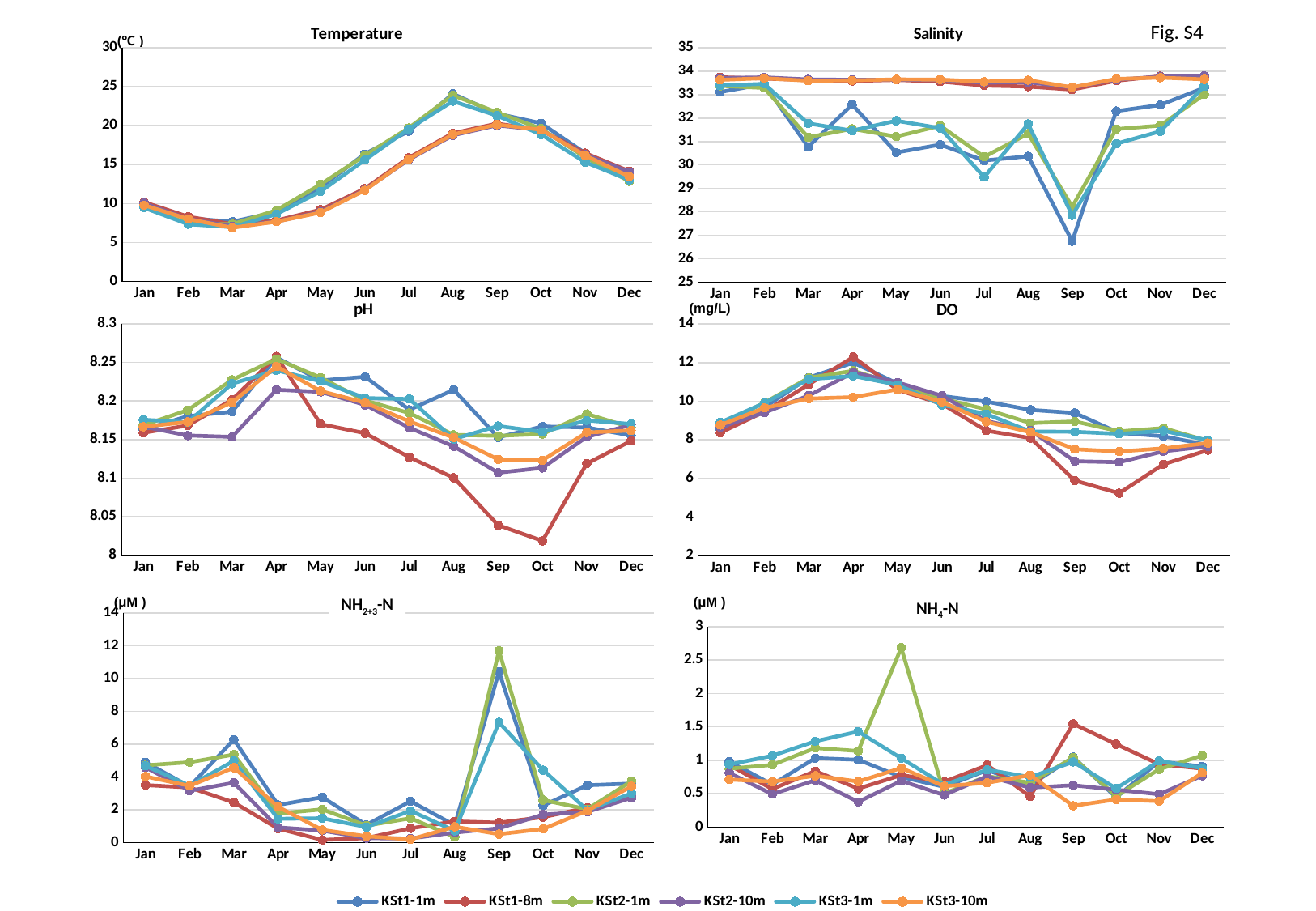

### Chart: Temperature
| Category | KSt1-1m | KSt1-8m | KSt2-1m | KSt2-10m | KSt3-1m | KSt3-10m |
|---|---|---|---|---|---|---|
| Jan | 9.828600000000002 | 10.1756 | 9.487200000000001 | 10.0266 | 9.451 | 9.790600000000001 |
| Feb | 8.1124 | 8.3104 | 7.446800000000001 | 8.012800000000002 | 7.330200000000001 | 8.0084 |
| Mar | 7.665 | 7.357399999999999 | 7.3398 | 7.071800000000001 | 6.956 | 6.8824 |
| Apr | 8.902800000000003 | 7.815999999999999 | 9.129999999999997 | 7.668599999999999 | 8.6326 | 7.669999999999998 |
| May | 12.0702 | 9.2004 | 12.4526 | 8.9154 | 11.5446 | 8.8398 |
| Jun | 16.3588 | 11.892 | 16.1642 | 11.7372 | 15.5632 | 11.6782 |
| Jul | 19.278 | 15.8726 | 19.69479999999999 | 15.6184 | 19.5662 | 15.7154 |
| Aug | 24.0912 | 19.023 | 23.913 | 18.7556 | 23.138 | 18.85899999999999 |
| Sep | 21.5508 | 20.26 | 21.7062 | 20.0392 | 21.28439999999999 | 20.1398 |
| Oct | 20.3092 | 19.4544 | 19.6086 | 19.4392 | 18.86959999999999 | 19.4738 |
| Nov | 16.4666 | 16.4684 | 15.9402 | 16.27 | 15.3052 | 16.1726 |
| Dec | 13.746 | 14.1556 | 12.847208 | 14.052658 | 13.016 | 13.4594 |
### Chart: Salinity
| Category | KSt1-1m | KSt1-8m | KSt2-1m | KSt2-10m | KSt3-1m | KSt3-10m |
|---|---|---|---|---|---|---|
| Jan | 33.11220000000001 | 33.749 | 33.3764 | 33.7098 | 33.3888 | 33.6336 |
| Feb | 33.4498 | 33.6878 | 33.2884 | 33.7432 | 33.46380000000001 | 33.7036 |
| Mar | 30.7704 | 33.62060000000001 | 31.1832 | 33.65280000000001 | 31.7758 | 33.597 |
| Apr | 32.5782 | 33.5846 | 31.5426 | 33.6368 | 31.46419999999999 | 33.61280000000001 |
| May | 30.528 | 33.62900000000001 | 31.2126 | 33.636 | 31.8882 | 33.65300000000001 |
| Jun | 30.86739999999999 | 33.5592 | 31.67440000000001 | 33.63000000000001 | 31.5734 | 33.6446 |
| Jul | 30.1858 | 33.3884 | 30.34219999999999 | 33.5134 | 29.47799999999999 | 33.557 |
| Aug | 30.3682 | 33.3472 | 31.33460000000001 | 33.5348 | 31.7512 | 33.62020000000001 |
| Sep | 26.7402 | 33.2162 | 28.21359999999999 | 33.3092 | 27.8488 | 33.315 |
| Oct | 32.29960000000001 | 33.59580000000001 | 31.5316 | 33.648 | 30.9076 | 33.6736 |
| Nov | 32.5606 | 33.79020000000001 | 31.6812 | 33.7678 | 31.43539999999999 | 33.7286 |
| Dec | 33.29660000000001 | 33.7684 | 33.006526 | 33.80146 | 33.3598 | 33.6546 |Fig. S4
(℃ )
### Chart: pH
| Category | KSt1-1m | KSt1-8m | KSt2-1m | KSt2-10m | KSt3-1m | KSt3-10m |
|---|---|---|---|---|---|---|
| Jan | 8.1628 | 8.158800000000001 | 8.168600000000001 | 8.166800000000002 | 8.1758 | 8.16656 |
| Feb | 8.1804 | 8.168199999999999 | 8.1882 | 8.155200000000004 | 8.1724 | 8.172880000000003 |
| Mar | 8.186000000000002 | 8.201600000000001 | 8.227400000000001 | 8.1534 | 8.2224 | 8.19816 |
| Apr | 8.2564 | 8.257800000000001 | 8.2544 | 8.214599999999997 | 8.239800000000002 | 8.244599999999998 |
| May | 8.226600000000001 | 8.17 | 8.230199999999998 | 8.211799999999998 | 8.2256 | 8.212840000000003 |
| Jun | 8.231399999999997 | 8.1582 | 8.200600000000001 | 8.195000000000002 | 8.203800000000001 | 8.1978 |
| Jul | 8.188600000000001 | 8.127000000000002 | 8.1844 | 8.165200000000002 | 8.2026 | 8.17356 |
| Aug | 8.214599999999997 | 8.1004 | 8.156 | 8.1412 | 8.1506 | 8.152560000000001 |
| Sep | 8.152800000000003 | 8.039 | 8.154800000000002 | 8.107000000000001 | 8.1676 | 8.124239999999999 |
| Oct | 8.167000000000002 | 8.018600000000001 | 8.157200000000001 | 8.1132 | 8.1598 | 8.12316 |
| Nov | 8.165600000000003 | 8.1188 | 8.183200000000001 | 8.153400000000003 | 8.174800000000001 | 8.15916 |
| Dec | 8.1552 | 8.1482 | 8.165600000000003 | 8.169184 | 8.170200000000001 | 8.1616768 |
### Chart: DO
| Category | KSt1-1m | KSt1-8m | KSt2-1m | KSt2-10m | KSt3-1m | KSt3-10m |
|---|---|---|---|---|---|---|
| Jan | 8.5258 | 8.3658 | 8.771800000000002 | 8.6498 | 8.901000000000002 | 8.760200000000001 |
| Feb | 9.689 | 9.4596 | 9.931199999999999 | 9.411800000000001 | 9.905200000000004 | 9.6504 |
| Mar | 11.2238 | 10.8742 | 11.2118 | 10.2978 | 11.1376 | 10.1332 |
| Apr | 11.9962 | 12.2872 | 11.571 | 11.4844 | 11.3048 | 10.2104 |
| May | 10.913 | 10.5848 | 10.861 | 10.9716 | 10.8454 | 10.6108 |
| Jun | 10.2684 | 9.857200000000004 | 10.1426 | 10.2826 | 9.808800000000002 | 9.966600000000003 |
| Jul | 9.978200000000001 | 8.47 | 9.573600000000003 | 8.974 | 9.336200000000002 | 8.931600000000001 |
| Aug | 9.552200000000003 | 8.085 | 8.8644 | 8.481000000000003 | 8.423200000000001 | 8.3884 |
| Sep | 9.387000000000002 | 5.8808 | 8.95 | 6.884399999999998 | 8.411000000000001 | 7.505600000000001 |
| Oct | 8.3544 | 5.2294 | 8.434600000000001 | 6.8342 | 8.304200000000002 | 7.389 |
| Nov | 8.1788 | 6.728400000000001 | 8.602 | 7.396999999999999 | 8.459600000000004 | 7.5508 |
| Dec | 7.7254 | 7.460599999999999 | 7.939451 | 7.654604799999999 | 7.9722 | 7.8262 |(mg/L)
### Chart: NH4-N
| Category | KSt1-1m | KSt1-8m | KSt2-1m | KSt2-10m | KSt3-1m | KSt3-10m |
|---|---|---|---|---|---|---|
| Jan | 0.98194171781613 | 0.931456248614004 | 0.876433786769471 | 0.811389518207769 | 0.939517141452231 | 0.713204361924759 |
| Feb | 0.638551415140644 | 0.571390906046372 | 0.93186097368277 | 0.494165540213056 | 1.06851165943917 | 0.682545247546404 |
| Mar | 1.033723207350839 | 0.840078378482702 | 1.18453134491472 | 0.701820822565283 | 1.285179630040874 | 0.769126225837826 |
| Apr | 1.009023096130474 | 0.577826464123244 | 1.140314151889878 | 0.379344809775372 | 1.430832631717485 | 0.683272648659493 |
| May | 0.7512 | 0.7796 | 2.6848 | 0.694 | 1.0318 | 0.8858 |
| Jun | 0.6042 | 0.6792 | 0.5024 | 0.481 | 0.6342 | 0.6134 |
| Jul | 0.8532355 | 0.9295035 | 0.7630058 | 0.7604766 | 0.85901418 | 0.66461603 |
| Aug | 0.62866 | 0.45994 | 0.6645626 | 0.5921088 | 0.74888 | 0.77894 |
| Sep | 1.04820794 | 1.54737916 | 1.04108968 | 0.62750148 | 0.9770322 | 0.320704686 |
| Oct | 0.44666698 | 1.2426043 | 0.46646302 | 0.5609037 | 0.58066 | 0.41536 |
| Nov | 0.9844 | 0.9422 | 0.867 | 0.4934 | 0.9926 | 0.3892 |
| Dec | 0.90882 | 0.87634 | 1.07216 | 0.77028 | 0.88192 | 0.81118 |
### Chart: NOx-N
| Category | KSt1-1m | KSt1-8m | KSt2-1m | KSt2-10m | KSt3-1m | KSt3-10m |
|---|---|---|---|---|---|---|
| Jan | 4.899133143783123 | 3.505591280084128 | 4.710382626769193 | 4.583937569070224 | 4.677284674832985 | 4.02512429291337 |
| Feb | 3.410513253523375 | 3.356772154222352 | 4.899592419422435 | 3.159609683510991 | 3.473218948040208 | 3.457156609202941 |
| Mar | 6.282059336753255 | 2.444544099440061 | 5.364003332177237 | 3.646210806793751 | 4.978962730933516 | 4.566265846846629 |
| Apr | 2.289861583318773 | 0.849484602954697 | 1.768005683731321 | 0.92365277544237 | 1.444542959084798 | 2.175707253853869 |
| May | 2.7632 | 0.1632 | 2.024 | 0.7298 | 1.4806 | 0.7714 |
| Jun | 1.0776 | 0.2684 | 1.0346 | 0.2682 | 0.9422 | 0.3848 |
| Jul | 2.51968148 | 0.86573892 | 1.483202 | 0.2479848 | 1.9338283 | 0.193247024 |
| Aug | 1.05342 | 1.29464 | 0.3385836 | 0.6088346 | 0.71044 | 0.96354 |
| Sep | 10.42464 | 1.2133 | 11.7014694 | 0.86715942 | 7.33172 | 0.5128 |
| Oct | 2.25592 | 1.55016 | 2.58734502 | 1.7050676 | 4.41522 | 0.831 |
| Nov | 3.4966 | 2.1268 | 2.031 | 1.8742 | 1.978599999999999 | 1.9406 |
| Dec | 3.594859999999999 | 2.73086 | 3.7461 | 2.72994 | 3.0008 | 3.434279999999999 |(μM )
(μM )
NH2+3-N
NH4-N

## Slide 5
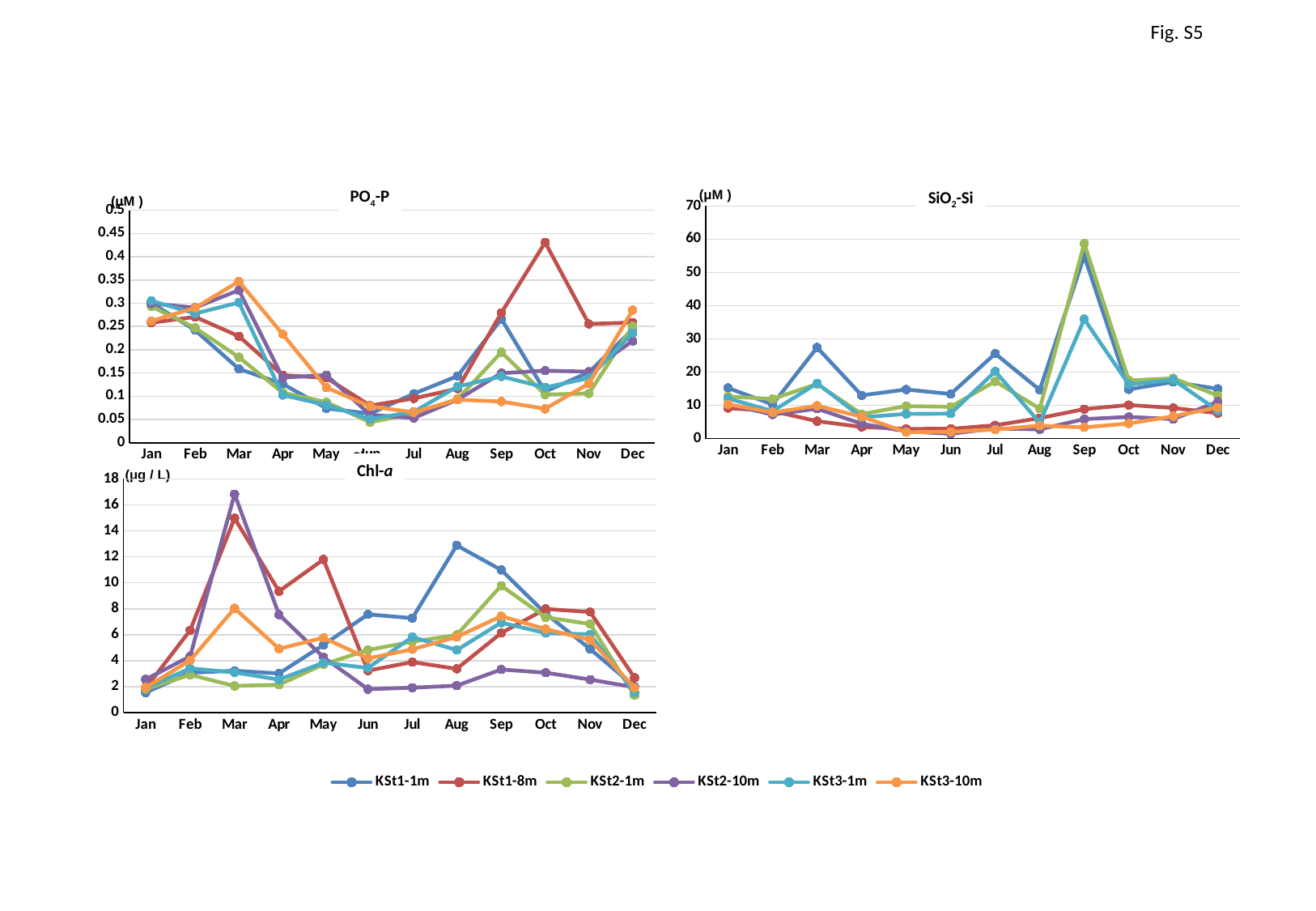

Fig. S5
### Chart: SiO2-Si
| Category | KSt1-1m | KSt1-8m | KSt2-1m | KSt2-10m | KSt3-1m | KSt3-10m |
|---|---|---|---|---|---|---|
| Jan | 15.21473297555053 | 9.143600920077196 | 12.63954359625054 | 10.26422576172887 | 12.05239376626377 | 10.29643207548762 |
| Feb | 10.25481807189842 | 8.24152892423065 | 11.89598931286992 | 7.209926189342772 | 8.24134 | 7.808135999999997 |
| Mar | 27.406772 | 5.210881999999999 | 16.401696 | 8.953835600000003 | 16.605766 | 9.858316 |
| Apr | 12.985574 | 3.4829512 | 7.369696 | 4.436114799999999 | 6.404715599999999 | 6.567082 |
| May | 14.7428 | 2.8962 | 9.755800000000004 | 2.1398 | 7.384799999999999 | 1.909799999999999 |
| Jun | 13.3956 | 2.9086 | 9.550600000000003 | 1.4298 | 7.492400000000001 | 2.0182 |
| Jul | 25.5369652 | 3.97469538 | 17.214956 | 3.022847 | 20.184829 | 2.707348614 |
| Aug | 14.6142642 | 6.13665802 | 8.971800000000002 | 2.7495228 | 4.745456459999999 | 3.858164672 |
| Sep | 54.97988047999999 | 8.844740940000001 | 58.73465445999998 | 5.832220240000001 | 35.92762594 | 3.3487002 |
| Oct | 14.79331814 | 10.07923986 | 17.47832428 | 6.495412259999999 | 16.26271863999999 | 4.520488552 |
| Nov | 17.0168 | 9.187200000000002 | 18.1206 | 5.8306 | 17.65580000000001 | 6.699800000000001 |
| Dec | 14.9464 | 7.617199999999999 | 12.9268 | 11.0866 | 8.408199999999997 | 9.314 |
### Chart: PO4-P
| Category | KSt1-1m | KSt1-8m | KSt2-1m | KSt2-10m | KSt3-1m | KSt3-10m |
|---|---|---|---|---|---|---|
| Jan | 0.30062577524622 | 0.258060244104308 | 0.293002595095507 | 0.299367072418094 | 0.30516278766106 | 0.261805344619334 |
| Feb | 0.241981548166877 | 0.270507110376818 | 0.246730080223961 | 0.290586401730485 | 0.277858155096465 | 0.290107937304307 |
| Mar | 0.158626101407983 | 0.229023620625515 | 0.183996732143581 | 0.328030299805619 | 0.30135044892626 | 0.347030986851686 |
| Apr | 0.126520920261388 | 0.145217800252994 | 0.107752286903803 | 0.14014343858417 | 0.102555454732339 | 0.233206954555348 |
| May | 0.074 | 0.1394 | 0.0872 | 0.1454 | 0.081 | 0.1186 |
| Jun | 0.0624 | 0.0798 | 0.044 | 0.06 | 0.0508 | 0.0782 |
| Jul | 0.10547102 | 0.0952982 | 0.0628688 | 0.053302 | 0.0665455 | 0.06547974 |
| Aug | 0.14349554 | 0.11635074 | 0.0941256 | 0.092829 | 0.120997 | 0.092559574 |
| Sep | 0.26611896 | 0.27955518 | 0.1946566 | 0.149639 | 0.14243082 | 0.088536624 |
| Oct | 0.1101794 | 0.4306137 | 0.10361816 | 0.15500776 | 0.118966 | 0.073006254 |
| Nov | 0.1514 | 0.2554 | 0.106 | 0.1528 | 0.139 | 0.1272 |
| Dec | 0.24356 | 0.25838 | 0.2507 | 0.21878 | 0.23572 | 0.28518 |PO4-P
(μM )
SiO2-Si
(μM )
### Chart: Chl-a
| Category | KSt1-1m | KSt1-8m | KSt2-1m | KSt2-10m | KSt3-1m | KSt3-10m |
|---|---|---|---|---|---|---|
| Jan | 1.540244551350527 | 1.881847603629339 | 1.810842450958484 | 2.559771277641297 | 1.886267132105845 | 1.935794603137098 |
| Feb | 3.06879520807355 | 6.340779987394123 | 2.908035553360853 | 4.332792947224479 | 3.409051660697567 | 4.011891071350115 |
| Mar | 3.224116683786778 | 14.97601892353586 | 2.052911324585978 | 16.83199002780614 | 3.092168921162117 | 8.035441176175375 |
| Apr | 3.018369646696065 | 9.346077536020553 | 2.146601226912501 | 7.547813258426985 | 2.543304400738309 | 4.920433213758883 |
| May | 5.2152 | 11.799 | 3.7152 | 4.262 | 3.8466 | 5.767599999999999 |
| Jun | 7.566599999999998 | 3.2322 | 4.824599999999998 | 1.8054 | 3.436799999999999 | 4.173119999999998 |
| Jul | 7.277712959999999 | 3.89020622 | 5.463919599999999 | 1.9197438 | 5.826458039999999 | 4.875608124 |
| Aug | 12.88336446 | 3.372357699999999 | 5.9790996 | 2.076646599999999 | 4.816232859999999 | 5.825540243999997 |
| Sep | 10.99208622 | 6.126070679999998 | 9.776765739999998 | 3.3220295 | 6.931170659999998 | 7.429624560000001 |
| Oct | 7.628787039999997 | 7.982214399999998 | 7.340957700000001 | 3.074118 | 6.12585018 | 6.430385464 |
| Nov | 4.899000000000001 | 7.7482 | 6.824999999999999 | 2.5446 | 6.0306 | 5.609479999999999 |
| Dec | 2.012282479999999 | 2.68668706 | 1.32802362 | 1.95217478 | 1.55005422 | 1.905844432 |
### Chart: Cyanobacteria
| Category | KSt1-1m | KSt1-8m | KSt2-1m | KSt2-10m | KSt3-1m | KSt3-10m |
|---|---|---|---|---|---|---|
| Jan | 54.37653009620222 | 45.29169324502691 | 54.77345201446629 | 52.56102266871581 | 53.67520519674484 | 56.55927608137404 |
| Feb | 39.21580464249507 | 38.11211419276637 | 33.34474549782981 | 36.91919766130171 | 31.07417931193468 | 32.22600827829985 |
| Mar | 16.976 | 15.56 | 17.066 | 20.748 | 19.308 | 17.714 |
| Apr | 9.597987795479165 | 9.939331996202478 | 11.35136459837242 | 13.41496543471956 | 13.98782896636516 | 13.93861918571418 |
| May | 12.434 | 13.848 | 16.00399999999999 | 17.246 | 16.33600000000001 | 16.506 |
| Jun | 21.34397471600141 | 19.4768147463046 | 20.3068898914762 | 20.6425253093766 | 23.7251651881638 | 25.8285401902786 |
| Jul | 32.39029087729524 | 32.0882285048091 | 33.2125537744098 | 35.28995861264937 | 32.74752958321188 | 40.60062663946371 |
| Aug | 41.89695190906441 | 57.70010434275721 | 38.53639463713203 | 65.4689903818128 | 37.78537044593414 | 41.25077178665113 |
| Sep | 42.85937044593413 | 45.24991372777616 | 41.34433576216846 | 52.18823549985427 | 39.3089309239289 | 38.60438764208686 |
| Oct | 58.03585028519772 | 71.38395162827463 | 61.08418874964661 | 77.94758235216493 | 87.26933407428159 | 88.15360728253262 |
| Nov | 78.24527614611125 | 85.88786692586494 | 83.23552524253074 | 90.28180359636777 | 85.66482070531534 | 87.19883888965518 |
| Dec | 54.68611053256733 | 52.43381088672651 | 61.48639794135571 | 62.07870002029951 | 67.31586011569557 | 61.62689108784503 |Chl-a
(μg / L)

## Slide 6
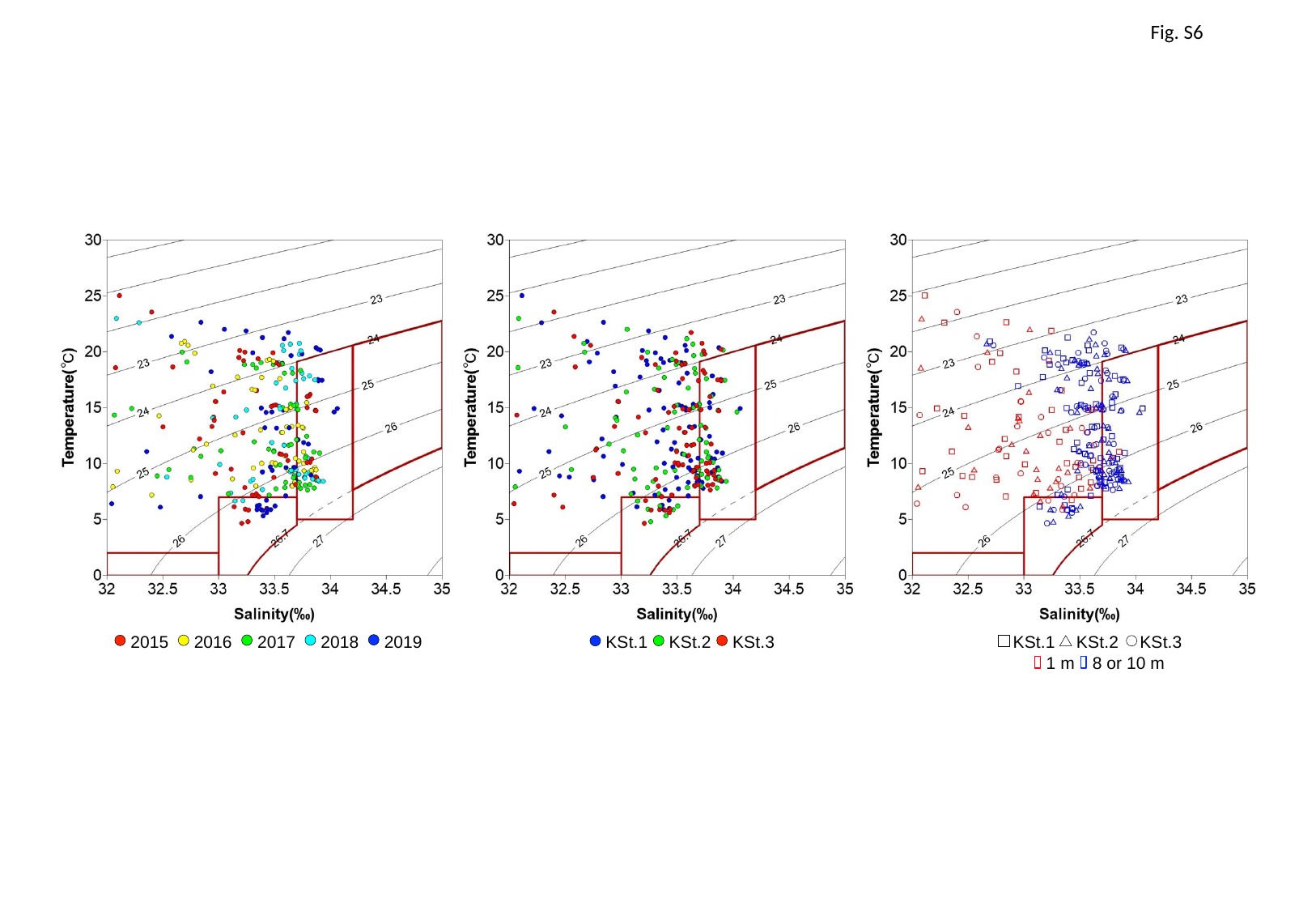

Fig. S6
KSt.1
KSt.2
KSt.3
2015
2016
2017
2018
2019
KSt.1
KSt.2
KSt.3
1 m
8 or 10 m

## Slide 7
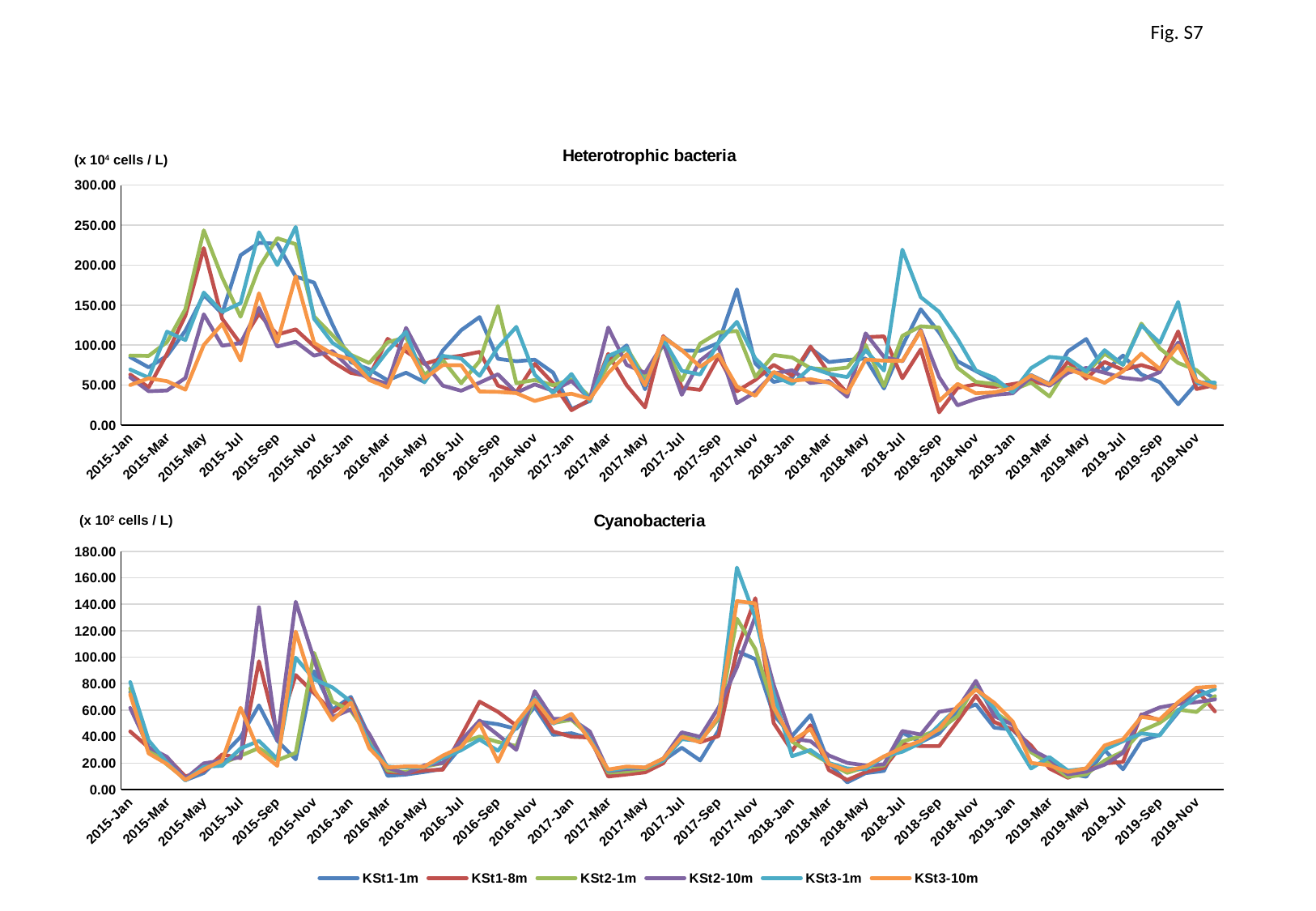

Fig. S7
### Chart: Heterotrophic bacteria
| Category | KSt1-1m | KSt1-8m | KSt2-1m | KSt2-10m | KSt3-1m | KSt3-10m |
|---|---|---|---|---|---|---|
| 2015-Jan | 84.99599875038155 | 63.16387612953711 | 86.95529180609836 | 59.71178836470274 | 69.5082536432868 | 50.1019224247584 |
| 2015-Feb | 72.21394405356237 | 47.2096326758431 | 86.58209312881895 | 42.35804987121099 | 59.33858968742334 | 58.49889266354471 |
| 2015-Mar | 86.58209312881895 | 89.10118420045484 | 103.8425319529908 | 43.29104656440948 | 116.8111859884497 | 54.95350522939049 |
| 2015-Apr | 118.0240816896078 | 136.9639145615369 | 145.0809857923637 | 58.87209134082411 | 106.3616230246268 | 44.31734292692782 |
| 2015-May | 162.7146232938149 | 221.4001152959994 | 243.6054365941232 | 138.5500089399743 | 165.7935123813699 | 100.2038448495168 |
| 2015-Jun | 139.3897059638529 | 133.0453284501033 | 184.6400455839792 | 99.27084815631827 | 141.6288980275294 | 126.5143515977139 |
| 2015-Jul | 212.4987149570954 | 101.6547906686645 | 135.5397208915526 | 102.5162719455176 | 152.4821860029968 | 80.69207959857268 |
| 2015-Aug | 228.005377940451 | 139.2728064245827 | 196.704891548122 | 146.7389774906428 | 241.214757518865 | 164.8300843045577 |
| 2015-Sep | 226.8567362379802 | 113.1412076933724 | 233.7485864528049 | 98.20886556125218 | 200.1508166555344 | 103.9520740736061 |
| 2015-Oct | 185.7927953746496 | 119.7458974825794 | 226.2824153867448 | 104.2392344992238 | 247.8194473080721 | 186.0799558002673 |
| 2015-Nov | 178.2478 | 98.57139999999998 | 135.8476 | 86.8147 | 132.8754 | 102.8257 |
| 2015-Dec | 125.8438 | 79.24570000000001 | 112.3985 | 92.57839999999999 | 102.8743 | 88.9367 |
| 2016-Jan | 78.6947 | 65.2345 | 87.9073 | 70.0274 | 87.5132 | 82.13869999999999 |
| 2016-Feb | 69.8247 | 61.1278 | 77.5359 | 58.2147 | 62.9357 | 56.3874 |
| 2016-Mar | 56.0101 | 107.9293 | 103.5859 | 51.7402 | 92.6969 | 47.1331 |
| 2016-Apr | 65.4891 | 92.17169999999999 | 109.6673 | 121.6093 | 116.7183 | 101.0356 |
| 2016-May | 53.78754072174134 | 76.37270819657314 | 62.65361886268774 | 77.99037859421952 | 53.56977739898128 | 58.34378654714674 |
| 2016-Jun | 93.27927825481505 | 83.6594039966222 | 81.40998066261693 | 49.3437331353075 | 86.62672839467174 | 75.04459122809132 |
| 2016-Jul | 118.6458931648478 | 86.8712 | 52.3406088454911 | 42.9350947731189 | 83.17777139574955 | 74.92102240091901 |
| 2016-Aug | 135.1234922458357 | 91.5781160252728 | 80.12636415852955 | 53.05858701895463 | 61.56662837449741 | 42.07352096496265 |
| 2016-Sep | 82.92647903503733 | 49.43279724296382 | 148.946008041355 | 63.54106835152211 | 97.1826536473291 | 41.60683515221137 |
| 2016-Oct | 79.83569999999997 | 41.0587 | 52.4781 | 40.5874 | 122.8791 | 39.9574 |
| 2016-Nov | 81.7659 | 76.5091 | 56.4319 | 50.8642 | 63.12860000000001 | 30.1543 |
| 2016-Dec | 65.6487 | 52.2783 | 49.8275 | 42.8527 | 40.1287 | 36.5297 |
| 2017-Jan | 20.0431699044095 | 18.6459296947271 | 57.1322849213691 | 55.19850447116871 | 63.89762565525749 | 39.17090656799259 |
| 2017-Feb | 30.2975 | 31.86809999999999 | 35.2974 | 32.6957 | 29.8367 | 32.8657 |
| 2017-Mar | 81.32379999999999 | 89.058 | 75.35089999999998 | 122.0348 | 86.37779999999998 | 65.1662 |
| 2017-Apr | 99.6446676893407 | 49.8701939156066 | 96.58162314941856 | 75.297665711188 | 96.58162314941856 | 88.64236102536198 |
| 2017-May | 44.79702639636064 | 22.20707291443519 | 58.96360739350034 | 65.28113675708964 | 51.7643 | 49.8751 |
| 2017-Jun | 110.6525 | 111.4182 | 100.889 | 102.0377 | 107.1108 | 109.8867 |
| 2017-Jul | 93.3271383257513 | 46.90286951755708 | 55.80484271170565 | 37.90517618153593 | 67.8655805876489 | 93.71001889324158 |
| 2017-Aug | 92.84853761638848 | 44.03126526138011 | 101.9419510942822 | 80.69207959857268 | 63.27101377776578 | 73.03446824876745 |
| 2017-Sep | 102.8478 | 85.4716 | 115.8745 | 98.2478 | 103.5744 | 88.2034 |
| 2017-Oct | 169.7118115400586 | 42.30830270767394 | 117.7357745032555 | 27.47168071742629 | 129.1264713860909 | 48.242951503773 |
| 2017-Nov | 77.15043434928778 | 56.47488370481361 | 60.20796923784367 | 41.25538114707572 | 83.27652342913197 | 36.66081433719258 |
| 2017-Dec | 53.98616001612693 | 75.331751653709 | 87.67964995526995 | 64.51537562210912 | 61.35661094031446 | 66.52549860143299 |
| 2018-Jan | 59.05932753537289 | 62.02665193342243 | 84.6166054153479 | 68.63134172262941 | 51.40171618556765 | 55.51768228608796 |
| 2018-Feb | 96.19874258192829 | 98.20886556125218 | 71.2157855531887 | 52.64607802991101 | 71.88582654629666 | 57.43208512353929 |
| 2018-Mar | 78.85819999999998 | 65.2975 | 69.4136 | 55.1397 | 64.1975 | 53.1756 |
| 2018-Apr | 81.36212059168065 | 40.2024595864775 | 71.88582654629666 | 35.41645249284922 | 60.01652895409855 | 40.29817972835007 |
| 2018-May | 83.27679999999998 | 109.9358 | 100.1624 | 114.7013 | 93.67429999999997 | 81.9505 |
| 2018-Jun | 45.75422781508629 | 110.9396444303034 | 48.72155221313583 | 85.19092626658326 | 68.24846115513918 | 80.59635945670013 |
| 2018-Jul | 98.80434980971906 | 58.59219233286457 | 111.6797041758581 | 79.77121726847005 | 219.4408222402826 | 80.424314953709 |
| 2018-Aug | 144.9876861230438 | 94.6058646903259 | 123.5287621794788 | 119.050378052126 | 160.1022325528592 | 117.7441826816483 |
| 2018-Sep | 115.7848896259314 | 15.95424345369401 | 121.9426678010414 | 60.08498704198212 | 141.7221976968492 | 30.22909285963075 |
| 2018-Oct | 79.86451693778989 | 46.55653499060417 | 71.65414603764327 | 24.72441236975973 | 107.8544177337443 | 51.5014174645561 |
| 2018-Nov | 67.539286529647 | 50.98629812574 | 54.09273276495 | 32.87532976453 | 68.2976089341 | 39.813234623 |
| 2018-Dec | 55.4298363208 | 47.6294523194 | 51.3298326051 | 37.824185432 | 59.0254149832 | 40.97322514312001 |
| 2019-Jan | 43.782323876 | 51.27649274500001 | 43.928732534 | 39.76132864 | 41.092827649 | 46.32823876000001 |
| 2019-Feb | 62.5478 | 55.2841 | 53.20780000000001 | 60.2385 | 71.2485 | 62.0318 |
| 2019-Mar | 51.5014174645561 | 49.728723747479 | 35.82707301882164 | 49.16892573155992 | 85.27589775834109 | 50.94161944863702 |
| 2019-Apr | 92.17452197 | 79.219824168 | 72.013821987 | 65.25172186999998 | 83.248729857 | 70.12752975 |
| 2019-May | 107.6678183951046 | 58.21899365558517 | 65.21646885457376 | 71.45872845969998 | 67.26906157961044 | 61.76438108973939 |
| 2019-Jun | 67.4291 | 78.9213 | 89.0542 | 65.4298 | 93.67209999999999 | 52.9186 |
| 2019-Jul | 86.9854 | 69.0875 | 75.32979999999999 | 59.1188 | 75.4219 | 67.2917 |
| 2019-Aug | 63.3504754681768 | 75.10623380247765 | 126.9808499443132 | 56.63289927714776 | 124.8349575499567 | 89.28778353909455 |
| 2019-Sep | 53.6781 | 68.24870000000001 | 95.87139999999998 | 66.2874 | 102.9841 | 70.2684 |
| 2019-Oct | 26.12390740955745 | 117.1843846657291 | 77.71862454343342 | 103.3760336063916 | 154.0377540470691 | 99.55074716427785 |
| 2019-Nov | 52.2873 | 45.09820000000001 | 68.7432 | 55.32080000000001 | 53.4209 | 55.4561 |
| 2019-Dec | 48.9081 | 50.09860000000001 | 48.7691 | 46.5409 | 53.09860000000001 | 47.0911 |(x 104 cells / L)
### Chart: Cyanobacteria
| Category | KSt1-1m | KSt1-8m | KSt2-1m | KSt2-10m | KSt3-1m | KSt3-10m |
|---|---|---|---|---|---|---|
| 2015-Jan | 73.71 | 43.85 | 76.51 | 61.58 | 81.16999999999997 | 71.84 |
| 2015-Feb | 30.78 | 31.72 | 31.25 | 31.96 | 37.13 | 27.26 |
| 2015-Mar | 21.38 | 24.2 | 18.8 | 24.67 | 20.44 | 19.27 |
| 2015-Apr | 7.05 | 9.870000000000003 | 7.99 | 8.219999999999999 | 6.81 | 7.28 |
| 2015-May | 12.45 | 14.1 | 18.8 | 19.73999999999999 | 17.15 | 15.27 |
| 2015-Jun | 25.429873580007 | 26.434073731523 | 19.644449457381 | 21.94262654688299 | 17.905825940819 | 21.852700951393 |
| 2015-Jul | 39.71145438647624 | 23.68114252404547 | 25.50276887204896 | 24.40979306324687 | 30.96764791605946 | 61.75313319731856 |
| 2015-Aug | 63.57475954532206 | 96.91052171378607 | 31.33197318566016 | 137.714951909064 | 36.79685222967065 | 28.96385893325562 |
| 2015-Sep | 36.79685222967065 | 42.0795686388808 | 22.04167881084232 | 37.16117749927135 | 23.13465461964442 | 17.85193821043428 |
| 2015-Oct | 22.77032935004372 | 86.52725153016614 | 27.68872048965316 | 141.9046925094724 | 99.64296123579132 | 119.3165257942291 |
| 2015-Nov | 89.37 | 72.85 | 103.25 | 98.24 | 83.27 | 75.16999999999997 |
| 2015-Dec | 60.28 | 58.27 | 66.37 | 54.59 | 77.15 | 52.27 |
| 2016-Jan | 69.96 | 68.27 | 59.59 | 60.57 | 66.41 | 65.64 |
| 2016-Feb | 40.27 | 37.75 | 39.27 | 42.01 | 34.27 | 31.18 |
| 2016-Mar | 10.32 | 13.24 | 14.31 | 16.43 | 17.34 | 16.64 |
| 2016-Apr | 11.34 | 12.41 | 13.12 | 11.78 | 16.53 | 17.51000000000001 |
| 2016-May | 13.28 | 14.4 | 17.28 | 18.28 | 16.98 | 17.28 |
| 2016-Jun | 15.59 | 14.98 | 19.92 | 20.05 | 23.67 | 25.59 |
| 2016-Jul | 32.38 | 40.51 | 35.17 | 36.97 | 29.67 | 32.07 |
| 2016-Aug | 51.28 | 66.39 | 40.06 | 52.08 | 37.78 | 49.87 |
| 2016-Sep | 49.28 | 58.67 | 35.87 | 41.26 | 29.27 | 21.07 |
| 2016-Oct | 45.78 | 48.27 | 32.87 | 29.97 | 47.25 | 50.27 |
| 2016-Nov | 62.41 | 65.64 | 68.51 | 74.31 | 68.91 | 67.61 |
| 2016-Dec | 41.29 | 43.82 | 50.24 | 53.34 | 49.68 | 50.15 |
| 2017-Jan | 42.51 | 39.85 | 52.67 | 53.27 | 56.87 | 57.14 |
| 2017-Feb | 38.57 | 39.27 | 40.18 | 43.85 | 38.29 | 36.87 |
| 2017-Mar | 10.86 | 9.81 | 12.75 | 13.86 | 14.24 | 15.11 |
| 2017-Apr | 12.16 | 11.38 | 13.52 | 15.49 | 16.42 | 17.35 |
| 2017-May | 14.38 | 12.96 | 15.63 | 16.45 | 16.51000000000001 | 16.65 |
| 2017-Jun | 21.86 | 19.87 | 21.87 | 23.46 | 21.95 | 23.39 |
| 2017-Jul | 31.57 | 41.26 | 40.41 | 43.17 | 38.02 | 39.7 |
| 2017-Aug | 21.98 | 35.71 | 36.81 | 39.83 | 36.71 | 35.82 |
| 2017-Sep | 44.58 | 40.35 | 53.27 | 61.87 | 54.68 | 55.27 |
| 2017-Oct | 104.5489220759449 | 105.512506611207 | 129.09222325858 | 92.2632192513523 | 167.6637091356166 | 142.341510618434 |
| 2017-Nov | 98.52651873055633 | 144.5376802893247 | 106.2351950126537 | 130.8066006618388 | 129.8430161265767 | 140.683342148276 |
| 2017-Dec | 56.36969531283664 | 50.10639583363256 | 61.66941025677854 | 79.01393189149749 | 72.50973627847789 | 62.39209865822517 |
| 2018-Jan | 40.47055048101114 | 29.20865622513453 | 36.13442007233138 | 38.42293334357904 | 25.17364598372419 | 36.73666040687024 |
| 2018-Feb | 56.18902321247531 | 48.54057096383183 | 27.88372748914905 | 36.55598830650858 | 29.81089655967339 | 45.7100413914992 |
| 2018-Mar | 19.85 | 14.68 | 19.95 | 25.74 | 20.03 | 19.28 |
| 2018-Apr | 5.359938977395821 | 7.166659981012391 | 12.5868229918621 | 20.1148271735978 | 15.89914483182581 | 14.27309592857089 |
| 2018-May | 12.38 | 13.2 | 16.82 | 18.18 | 15.2 | 17.3 |
| 2018-Jun | 14.08 | 16.35 | 18.2 | 19.01000000000001 | 24.98 | 25.02 |
| 2018-Jul | 42.98 | 34.18 | 36.27 | 44.18 | 28.57 | 31.57 |
| 2018-Aug | 35.84 | 32.78 | 40.27 | 41.51 | 35.27 | 36.74 |
| 2018-Sep | 42.38 | 32.78 | 45.17 | 58.64 | 48.61 | 45.86 |
| 2018-Oct | 58.71 | 51.41 | 55.39 | 61.08 | 61.98 | 62.87 |
| 2018-Nov | 64.32986199999999 | 70.92165434 | 79.64243119999999 | 82.08241731999999 | 76.23108739999998 | 75.62085229999997 |
| 2018-Dec | 46.74085735000001 | 50.96265860000001 | 58.63257945 | 55.43956821 | 61.4295643 | 65.48235678099998 |
| 2019-Jan | 45.2321 | 45.27981 | 48.96284 | 48.96218000000001 | 38.75238000000001 | 51.43972000000001 |
| 2019-Feb | 30.27 | 33.28 | 28.14 | 30.22 | 15.87 | 20.11 |
| 2019-Mar | 22.47 | 15.87 | 19.52 | 23.04 | 24.49 | 18.27 |
| 2019-Apr | 12.08 | 8.870000000000003 | 9.540000000000001 | 11.47 | 14.28 | 13.28 |
| 2019-May | 9.68 | 14.58 | 11.49 | 13.58 | 15.84 | 16.03 |
| 2019-Jun | 29.76 | 19.75 | 21.9 | 18.75 | 30.12 | 33.29 |
| 2019-Jul | 15.31 | 20.81 | 28.71 | 27.72 | 36.51 | 37.91 |
| 2019-Aug | 36.81 | 56.71 | 44.21 | 56.21 | 42.37 | 54.86 |
| 2019-Sep | 41.26 | 52.37 | 50.37 | 62.01 | 40.85 | 52.97 |
| 2019-Oct | 58.37 | 65.2 | 60.38 | 64.52 | 59.81 | 65.97 |
| 2019-Nov | 76.59 | 75.49 | 58.54 | 65.97 | 70.07 | 76.91 |
| 2019-Dec | 68.75 | 59.01 | 70.52 | 68.01 | 75.81 | 77.84 |(x 102 cells / L)

## Slide 8
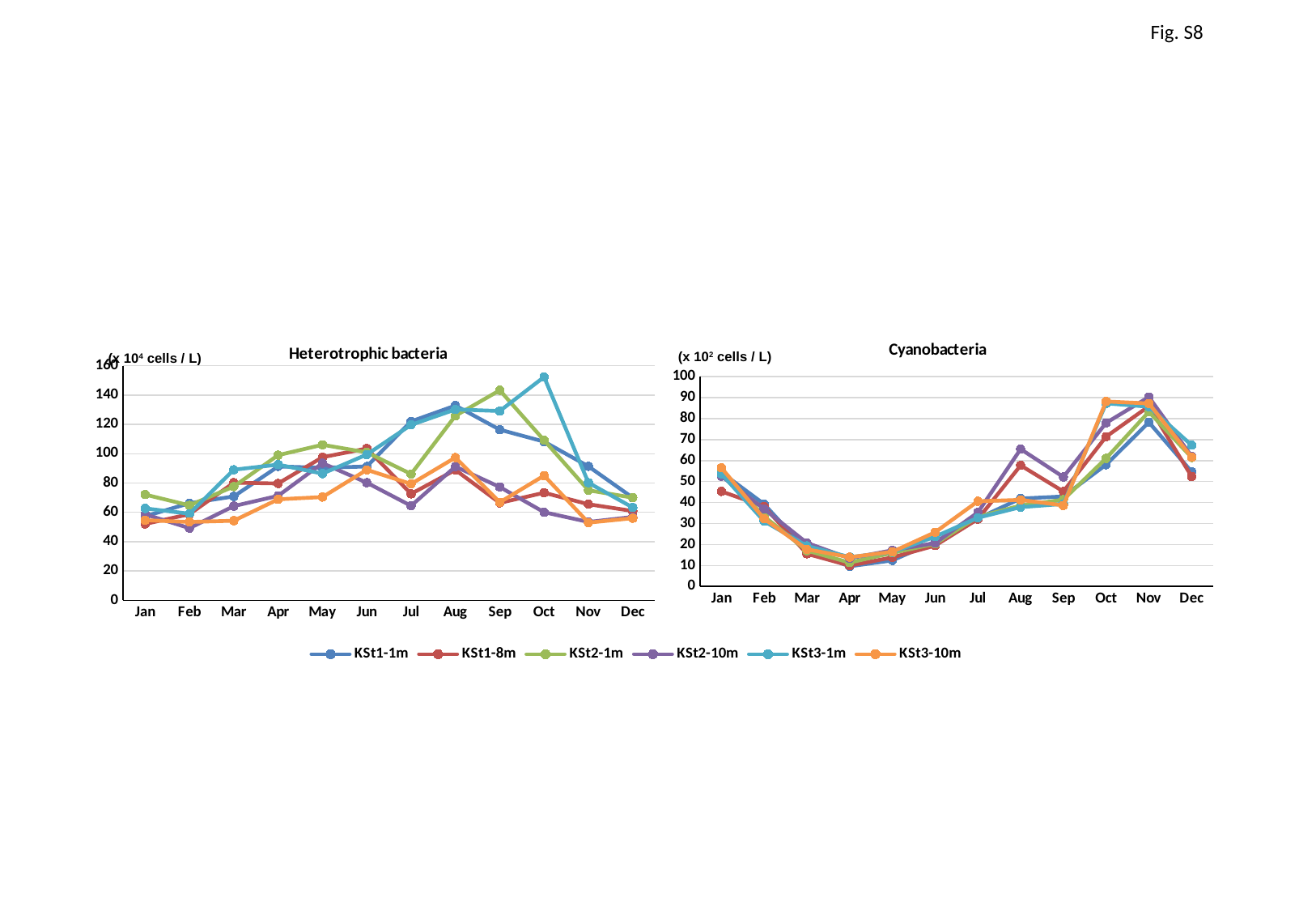

Fig. S8
### Chart: Cyanobacteria
| Category | KSt1-1m | KSt1-8m | KSt2-1m | KSt2-10m | KSt3-1m | KSt3-10m |
|---|---|---|---|---|---|---|
| Jan | 54.37653009620222 | 45.29169324502691 | 54.77345201446629 | 52.56102266871581 | 53.67520519674484 | 56.55927608137404 |
| Feb | 39.21580464249507 | 38.11211419276637 | 33.34474549782981 | 36.91919766130171 | 31.07417931193468 | 32.22600827829985 |
| Mar | 16.976 | 15.56 | 17.066 | 20.748 | 19.308 | 17.714 |
| Apr | 9.597987795479165 | 9.939331996202478 | 11.35136459837242 | 13.41496543471956 | 13.98782896636516 | 13.93861918571418 |
| May | 12.434 | 13.848 | 16.00399999999999 | 17.246 | 16.33600000000001 | 16.506 |
| Jun | 21.34397471600141 | 19.4768147463046 | 20.3068898914762 | 20.6425253093766 | 23.7251651881638 | 25.8285401902786 |
| Jul | 32.39029087729524 | 32.0882285048091 | 33.2125537744098 | 35.28995861264937 | 32.74752958321188 | 40.60062663946371 |
| Aug | 41.89695190906441 | 57.70010434275721 | 38.53639463713203 | 65.4689903818128 | 37.78537044593414 | 41.25077178665113 |
| Sep | 42.85937044593413 | 45.24991372777616 | 41.34433576216846 | 52.18823549985427 | 39.3089309239289 | 38.60438764208686 |
| Oct | 58.03585028519772 | 71.38395162827463 | 61.08418874964661 | 77.94758235216493 | 87.26933407428159 | 88.15360728253262 |
| Nov | 78.24527614611125 | 85.88786692586494 | 83.23552524253074 | 90.28180359636777 | 85.66482070531534 | 87.19883888965518 |
| Dec | 54.68611053256733 | 52.43381088672651 | 61.48639794135571 | 62.07870002029951 | 67.31586011569557 | 61.62689108784503 |
### Chart: Heterotrophic bacteria
| Category | KSt1-1m | KSt1-8m | KSt2-1m | KSt2-10m | KSt3-1m | KSt3-10m |
|---|---|---|---|---|---|---|
| Jan | 57.31510401323278 | 52.06949010053732 | 72.10804293536306 | 58.66607263970017 | 62.6827246266224 | 54.65149000776778 |
| Feb | 66.21653732709814 | 58.73969964741906 | 64.76779573640152 | 49.23060558022441 | 59.04906324674401 | 53.44317555741679 |
| Mar | 70.855122118675 | 80.22294158958677 | 77.60400099436252 | 64.27493445919387 | 89.07185674935816 | 54.2740049356055 |
| Apr | 91.3388983881258 | 79.68561844632418 | 99.04591149501579 | 71.28944628297226 | 92.58536099702876 | 68.884202686128 |
| May | 90.44876176140431 | 97.62693801251855 | 106.120306340977 | 93.59631055019669 | 86.41419027199235 | 70.4275224972806 |
| Jun | 91.30096240675086 | 103.5967753754058 | 100.9429556919464 | 80.25460151164181 | 99.45739751546803 | 88.99212045650108 |
| Jul | 122.0522992514827 | 72.62171050381724 | 86.13893532492148 | 64.44931203372849 | 119.6776520453356 | 79.40782716928847 |
| Aug | 132.8631138787792 | 88.91885724080787 | 125.8565637849452 | 91.2345842874888 | 130.1979179547888 | 97.39400794780613 |
| Sep | 116.4188009797898 | 66.44970967800604 | 143.2766324590402 | 77.27402419095128 | 129.1228335999425 | 66.85196041708964 |
| Oct | 108.2657462524111 | 73.37076396931732 | 109.1738120942154 | 60.0797522385603 | 152.3434380949952 | 85.06649438657483 |
| Nov | 91.39814417578694 | 65.5279763661107 | 75.06468040055873 | 53.42608218232115 | 80.19980647264637 | 52.98202979203851 |
| Dec | 69.9633192673854 | 60.9167607946218 | 70.00091651207397 | 56.86231221082185 | 63.2967251847029 | 56.01124474891059 |(x 102 cells / L)
(x 104 cells / L)

## Slide 9
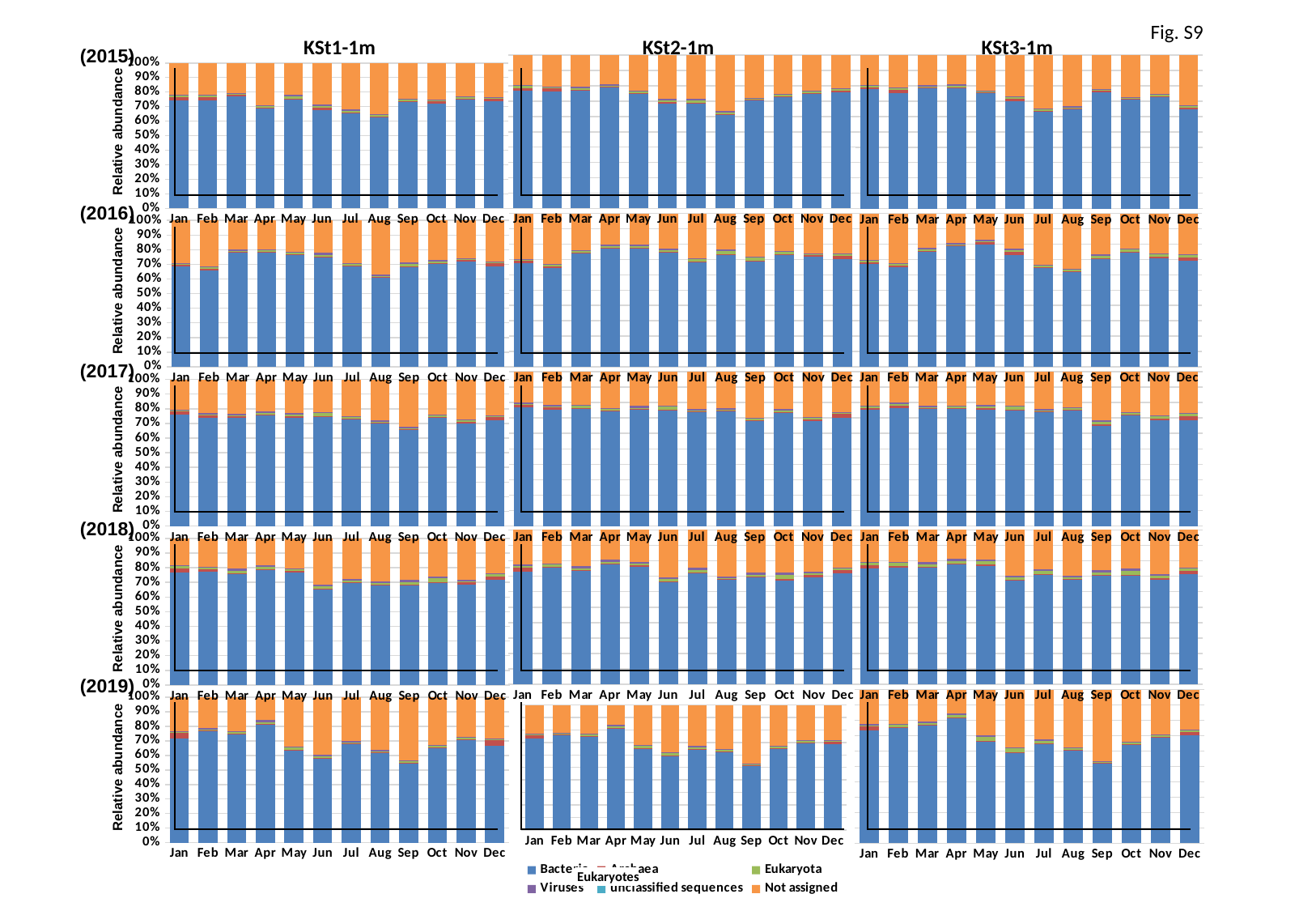

Fig. S9
KSt1-1m
KSt2-1m
KSt3-1m
(2015)
### Chart
| Category | Bacteria | Archaea | Eukaryota | Viruses | unclassified sequences | Not assigned |
|---|---|---|---|---|---|---|
| Jan | 238.994415283203 | 3.75000381469726 | 2.64850854873657 | 1.855869293212889 | 0.0 | 59.273021697998 |
| Feb | 218.627059936523 | 4.74171257019043 | 3.9814748764038 | 1.22289705276489 | 0.0 | 60.9732551574707 |
| Mar | 230.347229003906 | 1.97272300720214 | 2.11590456962585 | 2.67272162437438 | 0.0 | 56.8960876464843 |
| Apr | 213.435623168945 | 0.243956923484802 | 2.84743475914001 | 4.002418518066398 | 0.0 | 50.8650207519531 |
| May | 265.0102539062499 | 0.595903635025024 | 3.40686607360839 | 1.44549190998077 | 0.0 | 80.85561370849608 |
| Jun | 220.59114074707 | 3.69720864295959 | 3.35323762893676 | 2.68259000778198 | 0.0 | 83.6906585693359 |
| Jul | 202.5135955810539 | 1.70683348178863 | 3.932969093322749 | 2.78558325767517 | 0.0 | 111.030944824218 |
| Aug | 229.346939086914 | 0.396113991737365 | 2.935542583465569 | 3.1259229183197 | 0.0 | 116.482078552246 |
| Sep | 258.077423095703 | 2.20950555801391 | 2.00110387802124 | 1.85544693470001 | 0.0 | 75.08285522460929 |
| Oct | 252.276489257812 | 2.68252515792846 | 2.85532069206237 | 1.61555457115173 | 0.0 | 96.81584167480459 |
| Nov | 233.360031127929 | 1.12467503547668 | 3.5146095752716 | 1.67697083950042 | 0.0 | 81.8569259643554 |
| Dec | 234.09829711914 | 3.46444582939147 | 3.379693984985349 | 1.35429906845092 | 0.0 | 118.455337524414 |
### Chart
| Category | Bacteria | Archaea | Eukaryota | Viruses | unclassified sequences | Not assigned |
|---|---|---|---|---|---|---|
| Jan | 232.873413085937 | 5.032438278198239 | 4.07694339752197 | 1.96297264099121 | 0.0 | 58.4931182861328 |
| Feb | 217.7426757812499 | 6.04110765457153 | 3.15728664398193 | 1.33536529541015 | 0.0 | 57.96408843994139 |
| Mar | 233.209930419921 | 1.95237493515014 | 2.57080674171447 | 2.67867279052734 | 0.0 | 62.6198196411132 |
| Apr | 235.584243774414 | 0.205730944871902 | 1.86892938613891 | 2.98433804512023 | 0.0 | 56.0852317810058 |
| May | 217.454071044921 | 0.322311669588089 | 2.87380504608154 | 2.47724366188049 | 0.0 | 66.47045135498038 |
| Jun | 220.698196411132 | 3.47459053993225 | 3.30593633651733 | 2.50326681137084 | 0.0 | 92.5599746704101 |
| Jul | 207.47947692871 | 1.29960250854492 | 4.56599235534668 | 3.30446720123291 | 0.0 | 85.7373504638671 |
| Aug | 235.037826538085 | 0.304922014474868 | 3.61971950531005 | 3.2577474117279 | 0.0 | 139.54214477539 |
| Sep | 254.465499877929 | 1.49585974216461 | 2.08150291442871 | 2.40607643127441 | 0.0 | 99.8627548217773 |
| Oct | 273.3017578124999 | 3.598852157592769 | 2.76654434204101 | 1.983471512794489 | 0.0 | 95.7951126098632 |
| Nov | 248.404037475585 | 1.4909518957138 | 3.30050444602966 | 2.006189107894889 | 0.0 | 76.31980895996088 |
| Dec | 250.734283447265 | 4.166414737701408 | 2.88013911247253 | 2.12235498428344 | 0.0 | 71.4218597412109 |
### Chart
| Category | Bacteria | Archaea | Eukaryota | Viruses | unclassified sequences | Not assigned |
|---|---|---|---|---|---|---|
| Jan | 234.74624633789 | 6.37498378753662 | 3.12327098846435 | 1.76821243762969 | 0.0 | 69.3927001953125 |
| Feb | 219.452239990234 | 6.784182071685788 | 3.07945156097412 | 2.03356480598449 | 0.0 | 64.32673645019528 |
| Mar | 224.049102783203 | 3.10409903526306 | 1.72732484340667 | 2.72267079353332 | 0.0 | 59.49916458129879 |
| Apr | 236.289779663085 | 0.199328333139419 | 3.67732834815979 | 2.83903169631958 | 0.0 | 98.3769149780273 |
| May | 216.96435546875 | 0.371363282203674 | 5.19676494598388 | 2.72487807273864 | 0.0 | 63.89769744873039 |
| Jun | 225.437225341796 | 5.28168201446533 | 3.3715591430664 | 3.403608322143549 | 0.0 | 95.10873413085926 |
| Jul | 207.640426635742 | 1.55008125305175 | 3.77391934394836 | 2.63998198509216 | 0.0 | 101.917839050292 |
| Aug | 237.738159179687 | 0.367360591888427 | 3.147270679473869 | 3.0914146900177 | 0.0 | 131.805114746093 |
| Sep | 243.912490844726 | 2.428409099578849 | 2.65968608856201 | 1.68832242488861 | 0.0 | 83.0438766479492 |
| Oct | 260.5785217285149 | 5.05366849899292 | 1.92810642719268 | 1.85707092285156 | 0.0 | 91.581672668457 |
| Nov | 252.57469177246 | 1.44415593147277 | 3.1116349697113 | 2.25556325912475 | 0.0 | 77.57872009277337 |
| Dec | 250.813247680664 | 4.87526941299438 | 2.89876317977905 | 2.16603446006774 | 0.0 | 79.26332092285149 |Relative abundance
(2016)
### Chart
| Category | Bacteria | Archaea | Eukaryota | Viruses | unclassified sequences | Not assigned |
|---|---|---|---|---|---|---|
| Jan | 237.984970092773 | 4.40285396575927 | 2.739187002182 | 1.63251888751983 | 0.0 | 104.567321777343 |
| Feb | 231.942596435546 | 3.52325701713562 | 3.476762533187859 | 1.77023863792419 | 0.0 | 118.421730041503 |
| Mar | 223.252807617187 | 1.16687774658203 | 3.23754405975341 | 1.71162700653076 | 0.0 | 72.16226196289058 |
| Apr | 220.677703857421 | 2.34437537193298 | 2.53081774711608 | 3.02369022369384 | 0.0 | 58.662914276123 |
| May | 222.854095458984 | 2.27980804443359 | 2.87553668022155 | 2.73233270645141 | 0.0 | 58.7136497497558 |
| Jun | 205.755020141601 | 0.651822686195373 | 2.55757546424865 | 3.1928265094757 | 0.0 | 62.3705940246582 |
| Jul | 209.148895263671 | 0.699670314788818 | 4.164835929870598 | 2.66593766212463 | 0.0 | 89.07107543945307 |
| Aug | 230.255538940429 | 2.52083706855773 | 6.256736278533929 | 2.62109756469726 | 0.0 | 75.01638031005857 |
| Sep | 222.207778930664 | 1.16736984252929 | 5.71798944473266 | 2.309269666671749 | 0.0 | 91.31803131103509 |
| Oct | 234.308288574218 | 1.75666570663452 | 5.306112766265859 | 2.06581568717956 | 0.0 | 78.37965393066399 |
| Nov | 249.80908203125 | 3.786826133728019 | 3.29001235961914 | 1.225474357604979 | 0.0 | 90.538818359375 |
| Dec | 231.55191040039 | 8.15423965454101 | 2.92562413215637 | 1.1902539730072 | 0.0 | 86.9460525512695 |
### Chart
| Category | Bacteria | Archaea | Eukaryota | Viruses | unclassified sequences | Not assigned |
|---|---|---|---|---|---|---|
| Jan | 242.411315917968 | 4.75148200988769 | 2.962946891784659 | 1.77258396148681 | 0.0 | 102.877662658691 |
| Feb | 237.276947021484 | 3.850018978118889 | 3.612953424453729 | 2.010463476181029 | 0.0 | 113.859489440917 |
| Mar | 229.60237121582 | 1.52571153640747 | 2.31439757347106 | 2.42288494110107 | 0.0 | 58.50053405761709 |
| Apr | 225.344345092773 | 1.76995003223419 | 2.671662092208859 | 3.09009003639221 | 0.0 | 56.5651741027832 |
| May | 225.367874145507 | 2.13387584686279 | 3.10249519348144 | 1.92752826213836 | 0.0 | 63.00152969360349 |
| Jun | 219.503875732421 | 0.77014809846878 | 3.24006748199462 | 3.53956961631774 | 0.0 | 64.76634216308587 |
| Jul | 214.33299255371 | 0.838686168193817 | 3.871435403823848 | 2.64710330963134 | 0.0 | 90.4096221923828 |
| Aug | 200.721115112304 | 0.509194731712341 | 2.26462197303771 | 3.440085172653189 | 0.0 | 121.56980895996 |
| Sep | 207.176834106445 | 1.215256690979 | 5.636521339416499 | 3.6047887802124 | 0.0 | 86.42193603515618 |
| Oct | 247.738357543945 | 1.49878454208374 | 4.8185224533081 | 2.25984954833984 | 0.0 | 95.69342803955067 |
| Nov | 248.650268554687 | 3.73015904426574 | 2.310471534728999 | 2.22949123382568 | 0.0 | 89.35173034667957 |
| Dec | 239.268188476562 | 7.42933320999145 | 3.13478541374206 | 2.00568556785583 | 0.0 | 97.6076278686523 |
### Chart
| Category | Bacteria | Archaea | Eukaryota | Viruses | unclassified sequences | Not assigned |
|---|---|---|---|---|---|---|
| Jan | 236.151718139648 | 4.69562578201293 | 3.39354586601257 | 1.76920104026794 | 0.0 | 107.110717773437 |
| Feb | 222.088500976562 | 2.39175462722778 | 4.13162088394165 | 2.2628755569458 | 0.0 | 110.199043273925 |
| Mar | 212.850540161132 | 1.04552614688873 | 2.81555294990539 | 2.50856113433837 | 0.0 | 63.4367752075195 |
| Apr | 201.692230224609 | 1.6180715560913 | 1.81870210170745 | 3.11368036270141 | 0.0 | 48.88608551025389 |
| May | 242.064422607421 | 4.22896528244018 | 2.501001119613639 | 2.46202445030212 | 0.0 | 51.91688537597649 |
| Jun | 232.425476074218 | 7.05214262008667 | 4.03938150405883 | 2.885272502899169 | 0.0 | 73.0175247192382 |
| Jul | 204.180038452148 | 0.75795978307724 | 3.75744700431823 | 2.68752193450927 | 0.0 | 105.312507629394 |
| Aug | 205.839141845703 | 0.550447344779968 | 3.31351971626281 | 2.90176773071289 | 0.0 | 119.028823852539 |
| Sep | 216.756378173828 | 1.6068742275238 | 5.5867486000061 | 2.78342461585998 | 0.0 | 82.2814102172851 |
| Oct | 238.504287719726 | 1.39122939109802 | 4.1397099494934 | 2.012280225753779 | 0.0 | 73.4104766845703 |
| Nov | 240.331848144531 | 3.45713472366333 | 5.737494468688959 | 2.14546132087707 | 0.0 | 88.90533447265616 |
| Dec | 222.169998168945 | 6.71629619598388 | 4.94628000259399 | 1.8505824804306 | 0.0 | 86.42073822021479 |Relative abundance
(2017)
### Chart
| Category | Bacteria | Archaea | Eukaryota | Viruses | unclassified sequences | Not assigned |
|---|---|---|---|---|---|---|
| Jan | 246.607803344726 | 5.04139518737793 | 2.7524197101593 | 2.511805295944209 | 0.0 | 64.30263519287107 |
| Feb | 223.972747802734 | 4.167972087860099 | 2.017409801483149 | 2.52214956283569 | 0.0 | 63.69162750244139 |
| Mar | 231.013763427734 | 1.98820054531097 | 3.37476134300231 | 2.83819937705993 | 0.0 | 65.036865234375 |
| Apr | 239.896896362304 | 1.2718436717987 | 2.937420606613149 | 2.30574774742126 | 0.0 | 75.10958862304679 |
| May | 215.582305908203 | 1.57613885402679 | 1.78491413593292 | 3.64553809165954 | 0.0 | 63.2887420654296 |
| Jun | 210.925140380859 | 1.00556707382202 | 5.78796100616455 | 2.156911611557 | 0.0 | 61.3946304321289 |
| Jul | 240.483535766601 | 0.483262091875076 | 1.77516984939575 | 3.53301167488098 | 0.0 | 77.36621856689449 |
| Aug | 250.028915405273 | 1.22133100032806 | 2.55496835708618 | 3.24518418312072 | 0.0 | 79.41927337646479 |
| Sep | 219.314758300781 | 1.87752211093902 | 3.39970755577087 | 2.77866888046264 | 0.0 | 95.9376983642578 |
| Oct | 239.672164916992 | 1.20275235176086 | 3.49191451072692 | 3.10683703422546 | 0.0 | 78.9998550415039 |
| Nov | 222.161972045898 | 2.422195196151729 | 3.23000741004943 | 1.82657349109649 | 0.0 | 96.2313842773437 |
| Dec | 208.736831665039 | 7.11026859283447 | 2.52320289611816 | 1.3716846704483 | 0.0 | 77.66649627685538 |
### Chart
| Category | Bacteria | Archaea | Eukaryota | Viruses | unclassified sequences | Not assigned |
|---|---|---|---|---|---|---|
| Jan | 237.426376342773 | 3.355127573013299 | 2.85271000862121 | 2.14166140556335 | 0.0 | 69.64870452880858 |
| Feb | 221.663024902343 | 3.804063320159909 | 3.61322402954101 | 2.92938303947448 | 0.0 | 58.0501327514648 |
| Mar | 232.594604492187 | 2.006746530532829 | 1.56961095333099 | 2.819863796234129 | 0.0 | 67.8601913452148 |
| Apr | 238.726303100585 | 1.3361918926239 | 3.16579103469848 | 2.479869604110709 | 0.0 | 68.5194091796875 |
| May | 229.424530029296 | 2.230343103408809 | 3.350436925888059 | 2.62740755081176 | 0.0 | 65.88755035400389 |
| Jun | 209.028244018554 | 0.788168787956237 | 5.574135303497309 | 2.2028305530548 | 0.0 | 61.3136520385742 |
| Jul | 244.905075073242 | 0.523389458656311 | 1.91634738445281 | 2.87246036529541 | 0.0 | 79.64998626708979 |
| Aug | 254.935241699218 | 1.51699292659759 | 3.16803526878356 | 2.9736635684967 | 0.0 | 78.11508178710929 |
| Sep | 235.7451629638669 | 3.31322121620178 | 5.50124454498291 | 3.62009334564209 | 0.0 | 114.400939941406 |
| Oct | 242.163482666015 | 1.21843266487121 | 2.82418155670166 | 2.35415720939636 | 0.0 | 88.453369140625 |
| Nov | 216.28369140625 | 2.13893723487854 | 4.691019058227529 | 1.98204696178436 | 0.0 | 89.61833190917959 |
| Dec | 206.339370727539 | 9.14698123931884 | 3.51103448867797 | 1.52759301662445 | 0.0 | 81.3482589721679 |
### Chart
| Category | Bacteria | Archaea | Eukaryota | Viruses | unclassified sequences | Not assigned |
|---|---|---|---|---|---|---|
| Jan | 249.898849487304 | 6.004380702972409 | 2.82586765289306 | 2.18170142173767 | 0.0 | 67.15785217285146 |
| Feb | 222.827606201171 | 3.87827324867248 | 2.831773042678829 | 2.49576163291931 | 0.0 | 69.0260238647461 |
| Mar | 226.415618896484 | 3.26284170150756 | 2.58167171478271 | 2.645648956298819 | 0.0 | 72.4260559082031 |
| Apr | 234.18603515625 | 1.49101293087005 | 3.064660310745229 | 2.371249437332149 | 0.0 | 68.22731018066399 |
| May | 229.492370605468 | 4.12623119354248 | 3.25017714500427 | 3.114688873291009 | 0.0 | 71.30835723876947 |
| Jun | 222.784469604492 | 1.34018850326538 | 5.324012279510487 | 2.515915155410759 | 0.0 | 66.26934814453116 |
| Jul | 235.126739501953 | 0.582301318645477 | 2.6529848575592 | 2.74836182594299 | 0.0 | 80.633674621582 |
| Aug | 227.406997680664 | 0.847980976104736 | 2.64732122421264 | 3.08318638801574 | 0.0 | 91.0846176147461 |
| Sep | 233.653762817382 | 1.5750412940979 | 2.944166183471669 | 2.97264409065246 | 0.0 | 115.29345703125 |
| Oct | 264.4493408203121 | 1.18458712100982 | 4.261514663696278 | 2.12176060676574 | 0.0 | 85.72062683105459 |
| Nov | 236.726760864257 | 3.54179072380065 | 3.14467740058898 | 1.51133286952972 | 0.0 | 92.89575195312499 |
| Dec | 229.506118774414 | 6.03909921646118 | 2.350498914718619 | 1.70180451869964 | 0.0 | 77.7510299682617 |Relative abundance
(2018)
### Chart
| Category | Bacteria | Archaea | Eukaryota | Viruses | unclassified sequences | Not assigned |
|---|---|---|---|---|---|---|
| Jan | 197.825241088867 | 6.326856136322019 | 3.68745636940002 | 1.70892012119293 | 0.0 | 61.03865432739249 |
| Feb | 205.393692016601 | 2.27061295509338 | 3.92671632766723 | 1.7728601694107 | 0.0 | 58.8961372375488 |
| Mar | 183.90737915039 | 0.911100447177887 | 2.00794029235839 | 3.57499384880065 | 0.0 | 58.7112312316894 |
| Apr | 216.299346923828 | 0.979707300662994 | 3.00485324859619 | 4.25374507904052 | 0.0 | 52.8854141235351 |
| May | 211.582870483398 | 2.30518436431884 | 3.431368112564079 | 2.19784760475158 | 0.0 | 58.2651100158691 |
| Jun | 220.5395507812499 | 0.456902056932449 | 5.03680086135864 | 3.27582454681396 | 0.0 | 101.069183349609 |
| Jul | 232.010009765625 | 0.355605751276016 | 4.857136249542228 | 4.260527610778799 | 0.0 | 78.62763214111318 |
| Aug | 238.592178344726 | 0.535140573978424 | 2.42786478996276 | 3.214000463485709 | 0.0 | 105.994140625 |
| Sep | 209.897811889648 | 0.724166572093963 | 4.526534557342519 | 3.84775161743164 | 0.0 | 83.88295745849607 |
| Oct | 183.983245849609 | 2.4694447517395 | 7.91336107254028 | 3.92499637603759 | 0.0 | 75.35263061523429 |
| Nov | 233.816955566406 | 4.565094470977779 | 4.28763389587402 | 2.81579184532165 | 0.0 | 92.00637817382805 |
| Dec | 228.854995727539 | 6.674105644226069 | 3.049757003784169 | 1.59251689910888 | 0.0 | 77.2538146972656 |
### Chart
| Category | Bacteria | Archaea | Eukaryota | Viruses | unclassified sequences | Not assigned |
|---|---|---|---|---|---|---|
| Jan | 201.83984375 | 5.00831985473632 | 3.57544493675231 | 1.79025101661682 | 0.0 | 55.92427444458 |
| Feb | 193.205368041992 | 2.16329908370971 | 4.95763731002807 | 1.6710512638092 | 0.0 | 52.83520126342769 |
| Mar | 184.902450561523 | 1.45666861534118 | 2.88814973831176 | 3.838972330093379 | 0.0 | 51.2310752868652 |
| Apr | 199.292358398437 | 0.838143169879913 | 3.61607980728149 | 4.187541007995599 | 0.0 | 48.037670135498 |
| May | 213.659759521484 | 2.66824126243591 | 5.45537805557251 | 3.03045845031738 | 0.0 | 53.6537704467773 |
| Jun | 210.177383422851 | 0.536898970603942 | 5.37911987304687 | 3.19100332260131 | 0.0 | 92.06973266601558 |
| Jul | 231.976440429687 | 0.547136306762695 | 6.9896273612976 | 3.315552473068229 | 0.0 | 82.37752532958979 |
| Aug | 230.26480102539 | 0.572487711906433 | 3.49249505996704 | 2.6417589187622 | 0.0 | 101.395889282226 |
| Sep | 208.611175537109 | 0.567652404308319 | 5.42589998245239 | 3.69295358657836 | 0.0 | 77.10861206054679 |
| Oct | 193.026611328125 | 1.98141825199127 | 6.975034236907948 | 4.101010799407949 | 0.0 | 69.48780822753899 |
| Nov | 206.83934020996 | 2.58483791351318 | 5.15805339813232 | 2.81263852119445 | 0.0 | 87.26734161376949 |
| Dec | 216.022659301757 | 6.25131702423095 | 4.05978298187255 | 1.63230729103088 | 0.0 | 74.15370941162107 |
### Chart
| Category | Bacteria | Archaea | Eukaryota | Viruses | unclassified sequences | Not assigned |
|---|---|---|---|---|---|---|
| Jan | 209.682647705078 | 7.47472143173217 | 4.03822135925293 | 1.99504196643829 | 0.0 | 49.61128997802729 |
| Feb | 205.820968627929 | 3.60692048072814 | 3.50121521949768 | 1.69128322601318 | 0.0 | 51.426658630371 |
| Mar | 180.159530639648 | 1.26745820045471 | 3.4582302570343 | 4.27662181854248 | 0.0 | 48.3438758850097 |
| Apr | 221.279800415039 | 1.74398374557495 | 4.980454921722409 | 3.86727380752563 | 0.0 | 51.14448928833 |
| May | 216.944641113281 | 2.66557621955871 | 3.19325470924377 | 2.02116870880126 | 0.0 | 57.09641647338859 |
| Jun | 212.729141235351 | 0.442878156900405 | 6.02637863159179 | 3.38731026649475 | 0.0 | 102.387771606445 |
| Jul | 226.344635009765 | 0.454336941242218 | 4.78656196594238 | 3.59510588645935 | 0.0 | 88.9086456298828 |
| Aug | 238.230346679687 | 0.935457587242126 | 3.40742969512939 | 3.387250185012809 | 0.0 | 103.103569030761 |
| Sep | 215.524261474609 | 0.462491422891616 | 4.96038341522216 | 4.65096998214721 | 0.0 | 88.93515014648428 |
| Oct | 199.621688842773 | 2.359829187393179 | 7.768744468688959 | 2.24300599098205 | 0.0 | 75.6313552856445 |
| Nov | 238.195693969726 | 6.34340095520019 | 2.7227931022644 | 2.57471370697021 | 0.0 | 99.28256225585929 |
| Dec | 225.7190704345699 | 7.612457752227779 | 4.186557769775389 | 1.80465221405029 | 0.0 | 75.564956665039 |
### Chart
| Category | Bacteria | Archaea | Eukaryota | Viruses | unclassified sequences | Not assigned |
|---|---|---|---|---|---|---|
| Jan | 224.811431884765 | 7.511554718017569 | 2.90552711486816 | 1.40586459636688 | 0.0 | 69.4512557983398 |
| Feb | 224.411437988281 | 1.37711119651794 | 2.73452925682067 | 2.27736759185791 | 0.0 | 65.87767791748037 |
| Mar | 221.885864257812 | 0.402669847011566 | 3.15855717658996 | 2.38277125358581 | 0.0 | 67.9292984008789 |
| Apr | 231.721984863281 | 0.174201190471649 | 3.2711112499237 | 3.70553874969482 | 0.0 | 45.6084518432617 |
| May | 192.960830688476 | 0.651257991790771 | 5.53927135467529 | 2.013412952423089 | 0.0 | 93.87180328369139 |
| Jun | 206.969696044921 | 0.310487568378448 | 5.90256690979003 | 3.6763048171997 | 0.0 | 130.959686279296 |
| Jul | 192.104125976562 | 0.459954440593719 | 4.40292263031005 | 3.05817031860351 | 0.0 | 98.3611984252929 |
| Aug | 204.585479736328 | 0.3766930103302 | 3.13951206207275 | 2.55812096595764 | 0.0 | 115.555122375488 |
| Sep | 235.839279174804 | 0.495150178670883 | 3.23994946479797 | 2.48838233947753 | 0.0 | 215.168014526367 |
| Oct | 245.115280151367 | 1.49629497528076 | 3.46018195152282 | 3.28007245063781 | 0.0 | 123.236511230468 |
| Nov | 232.756195068359 | 1.69328248500823 | 3.14451456069946 | 2.5683057308197 | 0.0 | 94.00341796875 |
| Dec | 231.8190917968749 | 6.50295114517211 | 3.578406333923339 | 2.2405047416687 | 0.0 | 94.15699005126949 |Relative abundance
(2019)
### Chart
| Category | Bacteria | Archaea | Eukaryota | Viruses | unclassified sequences | Not assigned |
|---|---|---|---|---|---|---|
| Jan | 220.823410034179 | 7.787411689758299 | 2.18451380729675 | 2.42053580284118 | 0.0 | 67.8471221923828 |
| Feb | 216.893829345703 | 1.34840393066406 | 3.73894119262695 | 2.40815448760986 | 0.0 | 64.08238983154288 |
| Mar | 232.565353393554 | 0.519788444042205 | 3.08874297142028 | 2.598942279815669 | 0.0 | 63.3692092895507 |
| Apr | 220.741317749023 | 0.155456110835075 | 3.372445583343499 | 3.38513588905334 | 0.0 | 41.7621726989746 |
| May | 192.577377319335 | 0.643316686153411 | 6.56822776794433 | 3.42983746528625 | 0.0 | 86.4016952514648 |
| Jun | 195.454437255859 | 0.442675024271011 | 8.13806629180908 | 2.530849218368529 | 0.0 | 123.879699707031 |
| Jul | 177.521987915039 | 0.603211045265197 | 4.768900871276848 | 2.5933027267456 | 0.0 | 89.02536773681638 |
| Aug | 185.55696105957 | 0.376589834690094 | 3.50729584693908 | 2.249841451644889 | 0.0 | 114.989196777343 |
| Sep | 225.596313476562 | 0.480127424001693 | 4.30038452148437 | 2.08920311927795 | 0.0 | 200.990432739257 |
| Oct | 240.64875793457 | 1.18524193763732 | 3.68155622482299 | 3.206647634506219 | 0.0 | 127.474395751953 |
| Nov | 225.872528076171 | 1.57701396942138 | 3.73650765419006 | 2.64392066001892 | 0.0 | 95.60019683837889 |
| Dec | 231.20750427246 | 6.838537216186518 | 3.01736378669738 | 1.30654597282409 | 0.0 | 86.2235107421875 |
### Chart
| Category | Bacteria | Archaea | Eukaryota | Viruses | unclassified sequences | Not assigned |
|---|---|---|---|---|---|---|
| Jan | 221.217498779296 | 11.0390615463256 | 3.23578429222106 | 1.7043832540512 | 0.0 | 71.37168121337889 |
| Feb | 240.715423583984 | 1.36430740356445 | 1.97127938270568 | 2.27338600158691 | 0.0 | 66.8910675048828 |
| Mar | 237.212707519531 | 0.413803249597549 | 2.95899748802185 | 1.95795881748199 | 0.0 | 73.7026214599609 |
| Apr | 236.137878417968 | 0.234002843499183 | 3.56032443046569 | 3.80593061447143 | 0.0 | 45.74078750610349 |
| May | 212.192016601562 | 1.21611452102661 | 4.793444156646719 | 1.83082938194274 | 0.0 | 113.660102844238 |
| Jun | 184.941818237304 | 0.421665161848068 | 4.13335990905761 | 3.45140743255615 | 0.0 | 125.989387512207 |
| Jul | 191.196945190429 | 1.23392391204833 | 2.1445767879486 | 3.156647920608519 | 0.0 | 84.8491744995117 |
| Aug | 202.321258544921 | 0.286394149065017 | 2.98575615882873 | 2.59569001197814 | 0.0 | 119.101089477539 |
| Sep | 256.70962524414 | 1.22091650962829 | 4.67316341400146 | 2.78425097465515 | 0.0 | 204.510543823242 |
| Oct | 247.801025390625 | 1.63348519802093 | 2.7323157787323 | 3.486736536026 | 0.0 | 124.161277770996 |
| Nov | 238.383514404296 | 1.38081157207489 | 2.88322854042053 | 2.458262920379629 | 0.0 | 91.82788848876949 |
| Dec | 240.051040649414 | 13.0182266235351 | 1.90765643119812 | 3.31423258781433 | 0.0 | 101.559524536132 |Relative abundance
Eukaryotes

## Slide 10
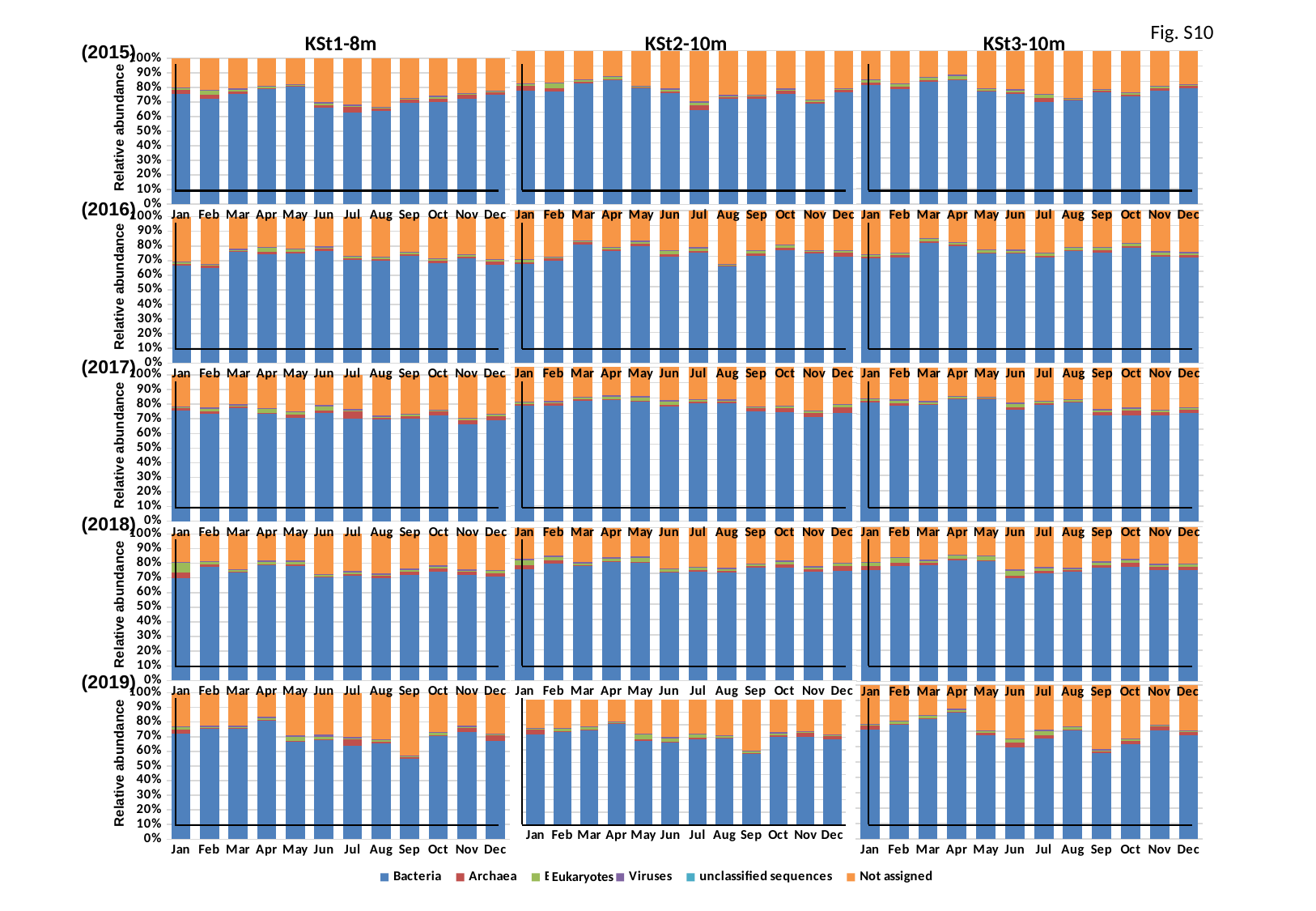

Fig. S10
KSt1-8m
KSt2-10m
KSt3-10m
(2015)
### Chart
| Category | Bacteria | Archaea | Eukaryota | Viruses | unclassified sequences | Not assigned |
|---|---|---|---|---|---|---|
| Jan | 232.7337341308589 | 10.4232063293457 | 3.58912301063537 | 1.90518510341644 | 0.0 | 66.7398681640625 |
| Feb | 219.6948547363279 | 5.641435623168939 | 10.5740747451782 | 1.27872538566589 | 0.0 | 61.8735275268554 |
| Mar | 240.951263427734 | 3.34871244430542 | 3.00242495536804 | 1.78851675987243 | 0.0 | 56.4981040954589 |
| Apr | 247.802536010742 | 1.66476356983184 | 5.142636299133299 | 2.09331655502319 | 0.0 | 49.9099426269531 |
| May | 252.077346801757 | 1.43304669857025 | 2.84596633911132 | 1.95836305618286 | 0.0 | 75.74417114257808 |
| Jun | 239.458633422851 | 4.381115436553949 | 3.638370990753169 | 2.65450382232666 | 0.0 | 81.1055297851562 |
| Jul | 218.78271484375 | 11.6130943298339 | 6.04146862030029 | 3.28003430366516 | 0.0 | 116.948738098144 |
| Aug | 248.830245971679 | 3.15864324569702 | 3.40789484977722 | 2.24472117424011 | 0.0 | 104.515090942382 |
| Sep | 265.380401611328 | 4.99944496154785 | 2.53687071800231 | 2.20493245124816 | 0.0 | 109.688201904296 |
| Oct | 260.1575927734371 | 8.08394527435302 | 2.415803194046019 | 2.959358930587759 | 0.0 | 88.55075073242176 |
| Nov | 269.035461425781 | 5.373557567596428 | 3.01690554618835 | 2.27854466438293 | 0.0 | 130.938827514648 |
| Dec | 270.7185974121089 | 6.05852699279785 | 2.90436458587646 | 1.659104347229 | 0.0 | 89.64134216308587 |
### Chart
| Category | Bacteria | Archaea | Eukaryota | Viruses | unclassified sequences | Not assigned |
|---|---|---|---|---|---|---|
| Jan | 247.963714599609 | 5.60284090042114 | 4.18100833892822 | 1.22147238254547 | 0.0 | 60.35908126831048 |
| Feb | 217.9696502685539 | 4.80901622772216 | 5.676985263824459 | 1.22375786304473 | 0.0 | 61.6727027893066 |
| Mar | 246.330795288085 | 4.130487918853759 | 3.40385794639587 | 1.92153263092041 | 0.0 | 53.30880737304679 |
| Apr | 255.450057983398 | 2.319465160369869 | 6.282370567321769 | 2.24953150749206 | 0.0 | 49.6004257202148 |
| May | 228.991027832031 | 0.336108326911926 | 2.91078424453735 | 2.80521178245544 | 0.0 | 75.53819274902338 |
| Jun | 241.065383911132 | 3.20269632339477 | 3.66390514373779 | 2.965650320053099 | 0.0 | 84.56355285644528 |
| Jul | 224.25390625 | 9.196311950683588 | 5.44989156723022 | 3.13749361038208 | 0.0 | 94.14307403564449 |
| Aug | 256.901916503906 | 1.55629444122314 | 2.97471332550048 | 2.075688838958739 | 0.0 | 116.367012023925 |
| Sep | 271.469085693359 | 3.69696879386901 | 2.74358391761779 | 2.22317719459533 | 0.0 | 92.8322219848632 |
| Oct | 265.2847595214839 | 4.51916599273681 | 2.878425598144529 | 2.19589281082153 | 0.0 | 103.24308013916 |
| Nov | 265.4179687499999 | 5.86236238479614 | 2.69279193878173 | 2.451045036315909 | 0.0 | 82.5028457641601 |
| Dec | 269.082794189453 | 5.660543918609609 | 2.24687337875366 | 1.54127621650695 | 0.0 | 77.51909637451169 |
### Chart
| Category | Bacteria | Archaea | Eukaryota | Viruses | unclassified sequences | Not assigned |
|---|---|---|---|---|---|---|
| Jan | 240.890670776367 | 8.29955005645752 | 3.78087878227233 | 1.14519393444061 | 0.0 | 63.60918426513669 |
| Feb | 210.7099609375 | 8.61144161224365 | 7.617507457733149 | 1.21657478809356 | 0.0 | 63.00744247436519 |
| Mar | 216.684143066406 | 4.565595626831048 | 4.096926212310789 | 2.15462136268615 | 0.0 | 59.38262176513668 |
| Apr | 239.331558227539 | 0.189866289496421 | 1.91295385360717 | 3.233851671218869 | 0.0 | 55.7063598632812 |
| May | 259.257171630859 | 0.868070185184478 | 1.68441188335418 | 2.2598922252655 | 0.0 | 57.2004928588867 |
| Jun | 226.140228271484 | 5.13928794860839 | 4.31820440292358 | 4.04990673065185 | 0.0 | 101.675559997558 |
| Jul | 228.338607788085 | 14.1690855026245 | 2.40717887878417 | 2.75944900512695 | 0.0 | 115.67268371582 |
| Aug | 244.362503051757 | 5.550577163696279 | 2.97752261161804 | 2.72844362258911 | 0.0 | 125.951713562011 |
| Sep | 264.948059082031 | 7.70602464675903 | 1.98109436035156 | 2.59975981712341 | 0.0 | 102.896354675292 |
| Oct | 255.365921020507 | 8.86476135253906 | 3.51422953605651 | 2.962054491043089 | 0.0 | 93.6471557617187 |
| Nov | 250.390563964843 | 10.3825721740722 | 1.28527927398681 | 2.379439115524289 | 0.0 | 82.35821533203116 |
| Dec | 255.235000610351 | 6.18327283859252 | 2.62706351280212 | 1.39753675460815 | 0.0 | 74.5149230957031 |Relative abundance
(2016)
### Chart
| Category | Bacteria | Archaea | Eukaryota | Viruses | unclassified sequences | Not assigned |
|---|---|---|---|---|---|---|
| Jan | 239.051849365234 | 5.33635473251342 | 3.6237506866455 | 1.65261316299438 | 0.0 | 110.142303466796 |
| Feb | 247.666915893554 | 5.49030923843383 | 3.819562911987299 | 1.93060326576232 | 0.0 | 122.264320373535 |
| Mar | 216.832626342773 | 1.92852246761322 | 2.42966175079345 | 1.75225961208343 | 0.0 | 62.91890716552729 |
| Apr | 237.381927490234 | 4.63421010971069 | 8.88722229003906 | 2.86341428756713 | 0.0 | 65.3111114501953 |
| May | 238.997436523437 | 3.91719007492065 | 4.747287273406979 | 3.1521987915039 | 0.0 | 69.11507415771477 |
| Jun | 228.455535888671 | 3.90739655494689 | 2.45079326629638 | 2.866965532302849 | 0.0 | 60.2987594604492 |
| Jul | 218.356185913085 | 3.11544704437255 | 3.29444670677185 | 3.02807831764221 | 0.0 | 82.51033782958977 |
| Aug | 240.327713012695 | 3.07516622543334 | 3.9709186553955 | 2.94961094856262 | 0.0 | 93.56301116943358 |
| Sep | 260.379241943359 | 4.39266157150268 | 3.99151110649108 | 1.84954178333282 | 0.0 | 85.78499603271479 |
| Oct | 252.251083374023 | 5.85363101959228 | 2.82217526435852 | 2.695778369903559 | 0.0 | 105.021392822265 |
| Nov | 252.722259521484 | 5.28474569320678 | 3.264168024063109 | 1.67113900184631 | 0.0 | 91.25693511962888 |
| Dec | 237.258224487304 | 8.531208992004387 | 3.545667886734 | 2.17029047012329 | 0.0 | 101.574760437011 |
### Chart
| Category | Bacteria | Archaea | Eukaryota | Viruses | unclassified sequences | Not assigned |
|---|---|---|---|---|---|---|
| Jan | 229.161590576171 | 4.634184360504149 | 3.92920041084289 | 1.14601683616638 | 0.0 | 96.3339004516601 |
| Feb | 243.677978515625 | 4.79591703414917 | 4.321103096008299 | 1.06011867523193 | 0.0 | 98.3162994384765 |
| Mar | 251.2136230468749 | 4.38744020462036 | 3.83880829811096 | 2.039618968963619 | 0.0 | 58.14689254760739 |
| Apr | 244.650314331054 | 3.7670681476593 | 3.77884936332702 | 1.35484862327575 | 0.0 | 65.6188507080078 |
| May | 196.930450439453 | 1.55460393428802 | 3.53192281723022 | 2.41827273368835 | 0.0 | 69.5655746459961 |
| Jun | 194.253814697265 | 1.63199007511138 | 3.14756202697753 | 2.22262525558471 | 0.0 | 69.68640136718749 |
| Jul | 229.124786376953 | 3.03794336318969 | 4.32080078125 | 2.38982343673706 | 0.0 | 91.35625457763669 |
| Aug | 239.179641723632 | 3.00950241088867 | 3.8352608680725 | 2.25470757484436 | 0.0 | 78.67607116699207 |
| Sep | 244.798263549804 | 5.060882568359369 | 4.45895290374755 | 2.472001314163199 | 0.0 | 80.9536666870117 |
| Oct | 254.345474243164 | 4.62539911270141 | 4.48384284973144 | 1.86229300498962 | 0.0 | 72.0632019042968 |
| Nov | 233.881286621093 | 3.85241651535034 | 6.34268569946289 | 2.09422636032104 | 0.0 | 90.2377548217773 |
| Dec | 222.00227355957 | 5.93767070770263 | 3.49690723419189 | 2.12825465202331 | 0.0 | 87.8309936523437 |
### Chart
| Category | Bacteria | Archaea | Eukaryota | Viruses | unclassified sequences | Not assigned |
|---|---|---|---|---|---|---|
| Jan | 235.727676391601 | 5.545175075530999 | 4.23840188980102 | 2.02803063392639 | 0.0 | 116.957199096679 |
| Feb | 248.844024658203 | 6.74276256561279 | 3.05742335319519 | 1.08858346939086 | 0.0 | 112.521682739257 |
| Mar | 232.0146484375 | 3.88381290435791 | 2.85658049583435 | 1.81623435020446 | 0.0 | 58.1238708496093 |
| Apr | 238.947189331054 | 3.77115201950073 | 3.45520877838134 | 2.36285185813903 | 0.0 | 77.9657135009765 |
| May | 240.525360107421 | 4.692396640777579 | 3.351139783859249 | 2.57441210746765 | 0.0 | 62.5065650939941 |
| Jun | 224.266006469726 | 5.319155216217038 | 6.04916191101074 | 2.56691813468933 | 0.0 | 83.1380996704101 |
| Jul | 222.002258300781 | 3.15496015548706 | 4.954357147216789 | 2.74321126937866 | 0.0 | 74.23781585693358 |
| Aug | 196.96907043457 | 0.327987164258956 | 2.10209941864013 | 2.32572698593139 | 0.0 | 107.918426513671 |
| Sep | 235.808212280273 | 4.884017944335929 | 5.39805269241333 | 2.260098695755 | 0.0 | 86.4267196655273 |
| Oct | 253.931594848632 | 5.78536128997802 | 5.41646003723144 | 2.099898099899289 | 0.0 | 76.2135314941406 |
| Nov | 249.236862182617 | 3.93365979194641 | 2.57744860649108 | 1.39319038391113 | 0.0 | 90.1469116210937 |
| Dec | 240.213165283203 | 8.785207748413079 | 4.02992916107177 | 1.33765757083892 | 0.0 | 90.7553634643554 |Relative abundance
(2017)
### Chart
| Category | Bacteria | Archaea | Eukaryota | Viruses | unclassified sequences | Not assigned |
|---|---|---|---|---|---|---|
| Jan | 244.637390136718 | 4.112247467041009 | 2.39633798599243 | 2.42707395553588 | 0.0 | 72.5822296142578 |
| Feb | 224.459014892578 | 3.76380538940429 | 2.52115225791931 | 2.82703614234924 | 0.0 | 65.06723785400389 |
| Mar | 226.364517211914 | 2.775610923767089 | 3.94268679618835 | 1.96880626678466 | 0.0 | 55.0149421691894 |
| Apr | 244.365005493164 | 1.52656686305999 | 4.22202682495117 | 3.399745464324949 | 0.0 | 56.5604057312011 |
| May | 255.54914855957 | 2.25622630119323 | 7.67073488235473 | 2.15841317176818 | 0.0 | 62.1309967041015 |
| Jun | 226.839614868164 | 4.055518627166738 | 6.48213815689086 | 3.34408593177795 | 0.0 | 64.7016372680664 |
| Jul | 251.175018310546 | 3.1156141757965 | 3.12273812294006 | 2.75940823554992 | 0.0 | 67.56275939941399 |
| Aug | 258.369934082031 | 3.84630131721496 | 3.09712743759155 | 2.58682084083557 | 0.0 | 70.7643432617187 |
| Sep | 259.223419189453 | 7.7758765220642 | 2.22777009010314 | 2.774053335189809 | 0.0 | 92.4684448242187 |
| Oct | 256.387054443359 | 8.92179489135742 | 3.319669961929319 | 3.22365856170654 | 0.0 | 89.7649230957031 |
| Nov | 241.123825073242 | 9.27213954925537 | 4.30037069320678 | 1.85256302356719 | 0.0 | 99.311050415039 |
| Dec | 211.050949096679 | 10.7140645980834 | 4.924744606018059 | 1.70552122592926 | 0.0 | 72.36997985839838 |
### Chart
| Category | Bacteria | Archaea | Eukaryota | Viruses | unclassified sequences | Not assigned |
|---|---|---|---|---|---|---|
| Jan | 248.737045288085 | 5.87915134429931 | 1.85305881500244 | 1.983534097671499 | 0.0 | 71.023567199707 |
| Feb | 221.370330810546 | 4.472836971282949 | 4.574927330017089 | 2.8712933063507 | 0.0 | 67.0829849243164 |
| Mar | 234.522415161132 | 2.328452110290519 | 2.002837419509879 | 2.96432209014892 | 0.0 | 60.9145164489746 |
| Apr | 236.096481323242 | 2.553815126419059 | 7.898444175720209 | 2.23477816581726 | 0.0 | 73.34963989257808 |
| May | 223.677139282226 | 7.01924562454223 | 4.39050674438476 | 2.46764636039733 | 0.0 | 78.12554168701169 |
| Jun | 224.854660034179 | 4.56916379928588 | 8.96406650543212 | 3.01679921150207 | 0.0 | 62.95949554443349 |
| Jul | 231.324661254882 | 16.1997699737548 | 2.73249149322509 | 3.086655378341669 | 0.0 | 77.1560440063476 |
| Aug | 259.592041015625 | 3.576650381088249 | 2.69793391227722 | 3.5749340057373 | 0.0 | 103.803520202636 |
| Sep | 258.657501220703 | 6.36632204055786 | 3.13835406303405 | 2.42517662048339 | 0.0 | 97.56901550292959 |
| Oct | 257.703491210937 | 9.23693656921386 | 1.90264439582824 | 2.7079861164093 | 0.0 | 83.6144638061523 |
| Nov | 227.866561889648 | 9.2102346420288 | 4.23372650146484 | 1.12538754940032 | 0.0 | 100.643684387207 |
| Dec | 230.306243896484 | 9.50444412231445 | 3.47789669036865 | 1.63215672969818 | 0.0 | 89.09918212890618 |
### Chart
| Category | Bacteria | Archaea | Eukaryota | Viruses | unclassified sequences | Not assigned |
|---|---|---|---|---|---|---|
| Jan | 240.478897094726 | 3.19501137733459 | 2.72971844673156 | 2.08495545387268 | 0.0 | 61.1616477966308 |
| Feb | 218.707290649414 | 4.18119382858276 | 4.24883699417114 | 2.99321031570434 | 0.0 | 59.0651741027832 |
| Mar | 227.50048828125 | 1.4114806652069 | 3.20752215385437 | 3.19755220413208 | 0.0 | 63.9951972961425 |
| Apr | 251.992141723632 | 2.08407330513 | 4.03726911544799 | 1.89835155010223 | 0.0 | 57.4466361999511 |
| May | 261.004486083984 | 1.91026592254638 | 2.491948604583739 | 2.10090136528015 | 0.0 | 61.04052352905269 |
| Jun | 231.927917480468 | 4.81662845611572 | 6.95234298706054 | 3.323318719863889 | 0.0 | 71.37879943847648 |
| Jul | 259.654754638671 | 4.01128959655761 | 2.9663496017456 | 2.57396411895751 | 0.0 | 72.431396484375 |
| Aug | 263.032623291015 | 3.04972505569458 | 2.37566184997558 | 1.91121590137481 | 0.0 | 69.7244415283203 |
| Sep | 249.188095092773 | 7.044049263000479 | 3.72428679466247 | 2.89343762397766 | 0.0 | 96.52503204345697 |
| Oct | 242.075439453125 | 11.059606552124 | 4.172819614410399 | 2.87542080879211 | 0.0 | 90.4239120483398 |
| Nov | 249.578643798828 | 7.893154621124259 | 4.065716743469229 | 1.91303813457489 | 0.0 | 97.87947845458976 |
| Dec | 215.525405883789 | 7.3478627204895 | 3.2654116153717 | 1.49538826942443 | 0.0 | 77.5500946044921 |Relative abundance
(2018)
### Chart
| Category | Bacteria | Archaea | Eukaryota | Viruses | unclassified sequences | Not assigned |
|---|---|---|---|---|---|---|
| Jan | 189.435317993164 | 9.596531867980953 | 18.4455680847167 | 1.89347374439239 | 0.0 | 51.81632614135739 |
| Feb | 210.585372924804 | 3.77975821495056 | 4.69908618927001 | 1.5987137556076 | 0.0 | 50.905418395996 |
| Mar | 252.0 | 2.0 | 5.0 | 1.0 | 0.0 | 82.0 |
| Apr | 243.373504638671 | 1.68679785728454 | 5.93190574645996 | 2.536811590194699 | 0.0 | 56.9062728881835 |
| May | 237.865402221679 | 2.966240406036369 | 6.14633321762085 | 3.215478420257559 | 0.0 | 54.48774337768549 |
| Jun | 243.659378051757 | 1.15285265445709 | 4.169182300567619 | 2.195843696594229 | 0.0 | 94.53252410888669 |
| Jul | 247.281341552734 | 3.30318284034729 | 4.73804950714111 | 2.938487529754629 | 0.0 | 87.43868255615229 |
| Aug | 254.935760498046 | 5.50699043273925 | 2.82534170150756 | 2.44838070869445 | 0.0 | 98.9018859863281 |
| Sep | 240.771896362304 | 5.76733589172363 | 5.025547504425039 | 3.72536492347717 | 0.0 | 78.14507293701168 |
| Oct | 273.116668701171 | 8.87333583831787 | 3.92347145080566 | 3.62879729270935 | 0.0 | 78.20786285400389 |
| Nov | 267.492645263671 | 6.55738830566406 | 3.4379014968872 | 3.45376110076904 | 0.0 | 89.74777221679678 |
| Dec | 232.759857177734 | 7.487930297851559 | 4.600528240203849 | 1.94266295433044 | 0.0 | 81.47287750244136 |
### Chart
| Category | Bacteria | Archaea | Eukaryota | Viruses | unclassified sequences | Not assigned |
|---|---|---|---|---|---|---|
| Jan | 186.84504699707 | 8.09223461151123 | 4.7494101524353 | 2.02548360824584 | 0.0 | 58.5721778869628 |
| Feb | 202.695999145507 | 5.324532985687249 | 8.26379203796386 | 1.77484428882598 | 0.0 | 53.47110366821279 |
| Mar | 232.813522338867 | 4.49940109252929 | 3.892646789550779 | 2.219224214553829 | 0.0 | 66.0115280151367 |
| Apr | 243.096389770507 | 3.07492232322692 | 5.69353008270263 | 3.17201042175292 | 0.0 | 55.00185775756829 |
| May | 244.593627929687 | 2.68676686286926 | 6.980186939239499 | 2.32629990577697 | 0.0 | 58.1574974060058 |
| Jun | 218.745391845703 | 4.41590166091918 | 10.8965768814086 | 2.90500020980834 | 0.0 | 90.67669677734368 |
| Jul | 235.874374389648 | 4.67006015777587 | 6.21076202392578 | 3.09377932548522 | 0.0 | 87.6158294677734 |
| Aug | 258.327606201171 | 4.14806604385376 | 2.76814413070678 | 2.9424500465393 | 0.0 | 96.55510711669916 |
| Sep | 242.792892456054 | 5.47363758087158 | 4.34554147720336 | 2.96528196334838 | 0.0 | 74.06758880615229 |
| Oct | 243.70637512207 | 8.95281219482421 | 5.02609395980835 | 3.58743691444396 | 0.0 | 67.8956604003906 |
| Nov | 271.112091064453 | 7.188176631927488 | 4.15189409255981 | 3.34899997711181 | 0.0 | 89.6428298950195 |
| Dec | 227.968353271484 | 7.164366245269769 | 4.503028869628899 | 2.28664207458496 | 0.0 | 75.1848602294921 |
### Chart
| Category | Bacteria | Archaea | Eukaryota | Viruses | unclassified sequences | Not assigned |
|---|---|---|---|---|---|---|
| Jan | 204.524765014648 | 7.40406131744384 | 8.67157554626464 | 1.82826781272888 | 0.0 | 57.6898880004882 |
| Feb | 206.521118164062 | 5.785524845123289 | 5.13403892517089 | 2.27610421180725 | 0.0 | 49.6563568115234 |
| Mar | 240.882125854492 | 3.0902817249298 | 3.218155384063719 | 2.2271339893341 | 0.0 | 73.00168609619139 |
| Apr | 244.779830932617 | 1.21891641616821 | 5.39178371429443 | 3.05415439605712 | 0.0 | 60.57108688354489 |
| May | 244.320877075195 | 2.46753811836242 | 8.14633560180664 | 2.8898446559906 | 0.0 | 59.85365676879879 |
| Jun | 229.685852050781 | 0.999177157878875 | 5.42973899841308 | 2.16926622390747 | 0.0 | 85.65314483642565 |
| Jul | 243.712158203125 | 3.66060137748718 | 5.784573078155509 | 2.99593496322631 | 0.0 | 87.4020004272461 |
| Aug | 244.75146484375 | 3.913446187973019 | 3.26594281196594 | 4.112434864044179 | 0.0 | 91.10685729980459 |
| Sep | 245.337753295898 | 3.882403373718259 | 3.45842242240905 | 2.27758312225341 | 0.0 | 77.9949645996093 |
| Oct | 252.992156982421 | 7.6042799949646 | 6.35183382034301 | 3.63388609886169 | 0.0 | 72.67971801757805 |
| Nov | 275.008026123046 | 6.653859138488759 | 3.24263453483581 | 3.275264739990229 | 0.0 | 97.86366271972649 |
| Dec | 235.666091918945 | 9.86926841735839 | 4.28952074050903 | 2.0725462436676 | 0.0 | 75.68043518066398 |Relative abundance
### Chart
| Category | Bacteria | Archaea | Eukaryota | Viruses | unclassified sequences | Not assigned |
|---|---|---|---|---|---|---|
| Jan | 211.434616 | 10.4709158 | 2.84360909 | 2.15659499 | 0.0 | 65.15868379999999 |
| Feb | 222.830734 | 2.21945739 | 5.87327194 | 2.62898827 | 0.0 | 67.37081909999999 |
| Mar | 243.101425 | 2.23592949 | 5.690054889999998 | 2.75443387 | 0.0 | 68.1033249 |
| Apr | 247.964569 | 0.41876599 | 2.73142481 | 2.0481751 | 0.0 | 52.9676018 |
| May | 219.063278 | 4.18606091 | 12.5488873 | 2.40133023 | 0.0 | 88.85851289999997 |
| Jun | 239.758881 | 3.40651584 | 9.10253811 | 3.67430854 | 0.0 | 109.189445 |
| Jul | 220.786026 | 3.88746524 | 8.924079899999999 | 2.532057999999999 | 0.0 | 87.10357669999999 |
| Aug | 240.947632 | 2.7594738 | 3.74427676 | 3.68225193 | 0.0 | 98.041069 |
| Sep | 257.172546 | 3.01270986 | 4.12936974 | 3.17872334 | 0.0 | 184.603622 |
| Oct | 238.622269 | 4.245381829999999 | 4.40219116 | 3.20306087 | 0.0 | 88.5165558 |
| Nov | 274.950195 | 11.4660177 | 2.17812324 | 3.09341884 | 0.0 | 98.25360109999998 |
| Dec | 251.648087 | 10.1673002 | 4.01224136 | 2.39219737 | 0.0 | 101.479042 |(2019)
### Chart
| Category | Bacteria | Archaea | Eukaryota | Viruses | unclassified sequences | Not assigned |
|---|---|---|---|---|---|---|
| Jan | 222.715347290039 | 8.200208663940426 | 2.64076948165893 | 1.47786176204681 | 0.0 | 79.33567047119139 |
| Feb | 217.803314208984 | 1.09983873367309 | 3.368608713150019 | 2.28992056846618 | 0.0 | 67.50753784179679 |
| Mar | 244.043563842773 | 2.498187303543089 | 3.9241988658905 | 2.77912235260009 | 0.0 | 60.5521507263183 |
| Apr | 258.090728759765 | 1.43402791023254 | 4.116826534271239 | 2.22308635711669 | 0.0 | 48.94678115844719 |
| May | 246.579483032226 | 4.46807527542114 | 5.57545518875122 | 1.47365081310272 | 0.0 | 106.835403442382 |
| Jun | 222.167343139648 | 11.2325811386108 | 7.24943828582763 | 2.32827234268188 | 0.0 | 129.514343261718 |
| Jul | 199.587768554687 | 6.314037799835199 | 7.59567594528198 | 3.09937381744384 | 0.0 | 89.30731201171868 |
| Aug | 258.532989501953 | 2.76705336570739 | 4.816911220550529 | 2.71279740333557 | 0.0 | 98.9747238159179 |
| Sep | 256.580780029296 | 3.377781391143789 | 3.15337753295898 | 3.96056175231933 | 0.0 | 192.421340942382 |
| Oct | 246.462539672851 | 7.782444953918449 | 3.80181789398193 | 3.02272200584411 | 0.0 | 138.112854003906 |
| Nov | 272.615234375 | 9.950501441955558 | 2.4583728313446 | 2.10850071907043 | 0.0 | 99.17599487304678 |
| Dec | 222.752120971679 | 6.63100862503051 | 2.879642963409419 | 1.70168626308441 | 0.0 | 97.63990020751949 |
### Chart
| Category | Bacteria | Archaea | Eukaryota | Viruses | unclassified sequences | Not assigned |
|---|---|---|---|---|---|---|
| Jan | 215.330825805664 | 9.56799221038818 | 3.76686692237854 | 1.70362436771392 | 0.0 | 69.11003875732418 |
| Feb | 224.790344238281 | 1.92007064819335 | 2.419429779052729 | 2.084648132324209 | 0.0 | 67.59633636474608 |
| Mar | 235.990509033203 | 0.852692008018493 | 2.52107572555542 | 3.02394533157348 | 0.0 | 70.1410675048828 |
| Apr | 250.688903808593 | 0.686362266540527 | 3.01999402046203 | 2.71113085746765 | 0.0 | 50.45252990722649 |
| May | 254.233444213867 | 1.03376543521881 | 8.87581062316894 | 4.125953674316398 | 0.0 | 111.01138305664 |
| Jun | 242.368270874023 | 0.561523675918579 | 6.362307548522939 | 4.03808784484863 | 0.0 | 101.982467651367 |
| Jul | 206.070007324218 | 14.7063789367675 | 2.436491012573239 | 2.62010216712951 | 0.0 | 97.7015380859375 |
| Aug | 238.264999389648 | 5.150522232055659 | 2.68909978866577 | 3.327312707901 | 0.0 | 114.946464538574 |
| Sep | 255.048065185546 | 4.125222206115719 | 3.62443494796752 | 2.869435310363759 | 0.0 | 197.596069335937 |
| Oct | 238.967163085937 | 2.45290160179138 | 4.43110370635986 | 2.696293115615839 | 0.0 | 90.82468414306639 |
| Nov | 260.738830566406 | 10.809865951538 | 1.66340935230255 | 2.7084891796112 | 0.0 | 80.5709609985351 |
| Dec | 240.695419311523 | 12.9807968139648 | 1.91539180278778 | 2.474878549575799 | 0.0 | 100.20677947998 |Relative abundance
Eukaryotes

## Slide 11
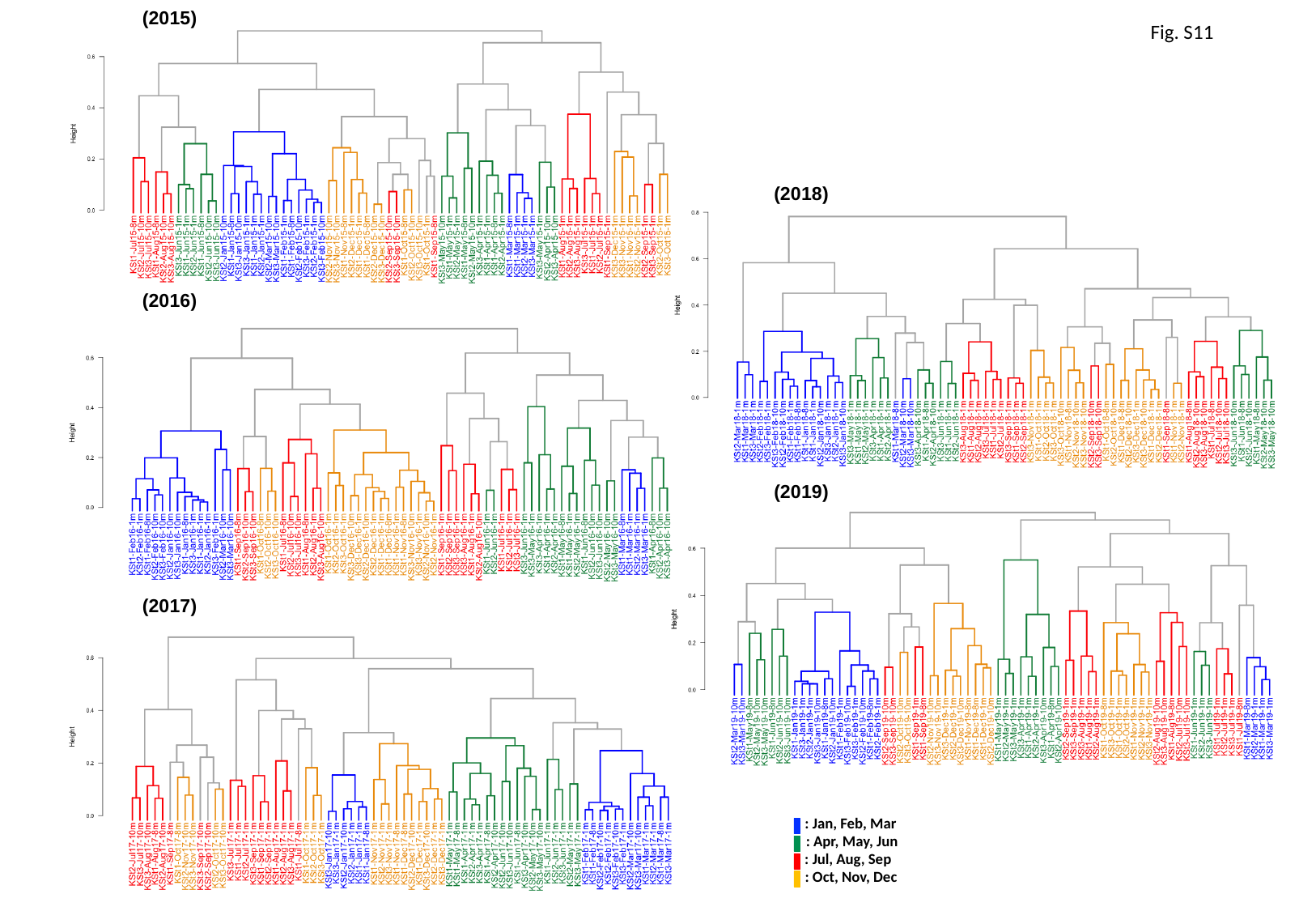

(2015)
Fig. S11
(2018)
(2016)
(2019)
(2017)
: Jan, Feb, Mar
: Apr, May, Jun
: Jul, Aug, Sep
: Oct, Nov, Dec

## Slide 12
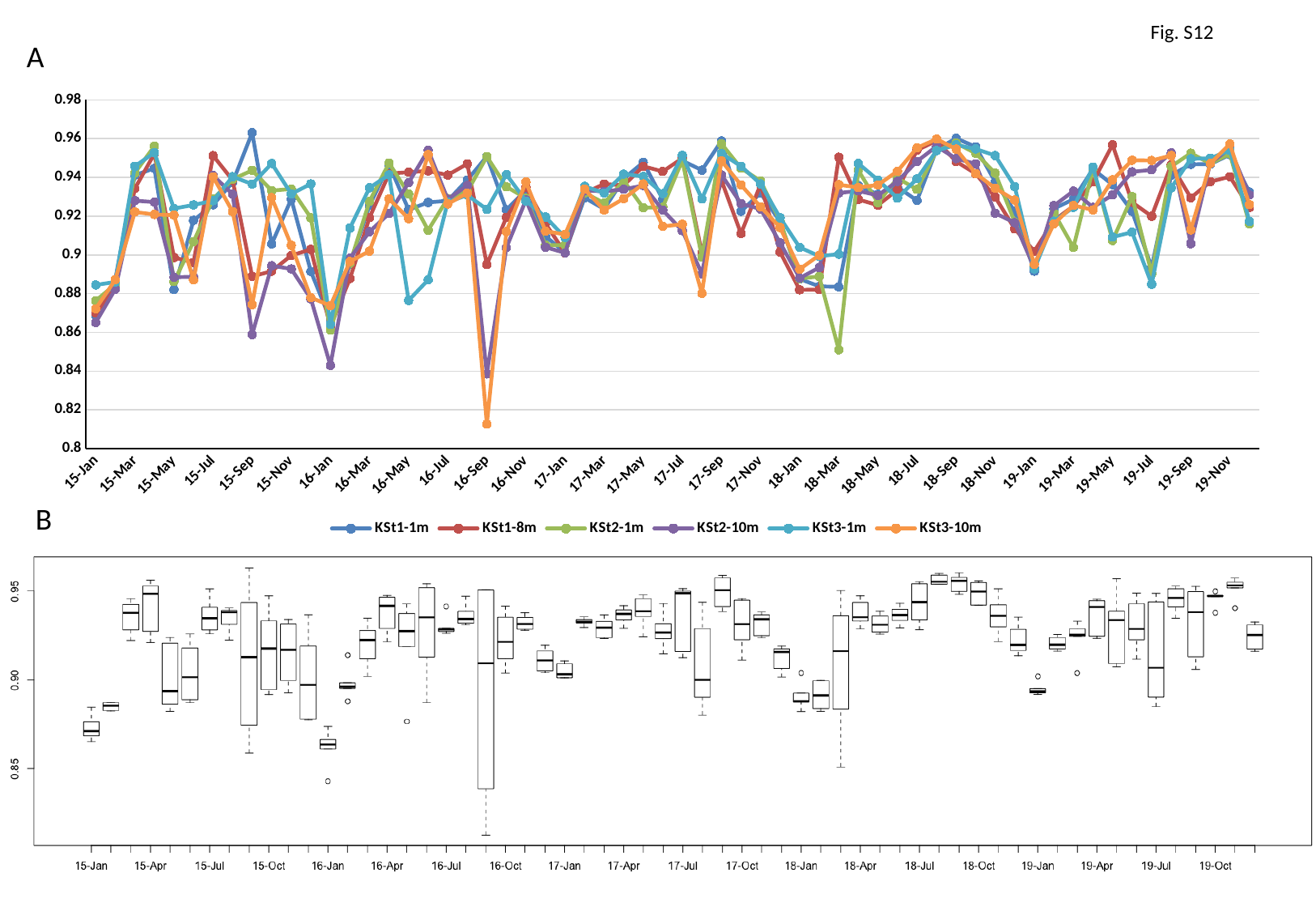

Fig. S12
A
### Chart
| Category | KSt1-1m | KSt1-8m | KSt2-1m | KSt2-10m | KSt3-1m | KSt3-10m |
|---|---|---|---|---|---|---|
| 15-Jan | 0.868548905838879 | 0.870022161997345 | 0.876365595120044 | 0.86510271138872 | 0.884474244051604 | 0.872244242711678 |
| 15-Feb | 0.887379946427102 | 0.882638723466441 | 0.884768991673484 | 0.88230494790486 | 0.886037043349621 | 0.887182621875122 |
| 15-Mar | 0.941153407242314 | 0.934205540129282 | 0.942351416976335 | 0.928009321902854 | 0.945722922431116 | 0.922158298268171 |
| 15-Apr | 0.944655244747515 | 0.952261606466294 | 0.956137719366523 | 0.927272903009327 | 0.952799617853556 | 0.920874789755923 |
| 15-May | 0.882071841151934 | 0.898676181657539 | 0.886242019842507 | 0.888466817268475 | 0.92392969990919 | 0.920520959441184 |
| 15-Jun | 0.917838823800911 | 0.896121969578111 | 0.906843521511228 | 0.888722828421754 | 0.925776730782018 | 0.88709362903504 |
| 15-Jul | 0.925860011597135 | 0.951255003218192 | 0.928888893559156 | 0.940973053189392 | 0.927977233857647 | 0.940378279744771 |
| 15-Aug | 0.9374618495862 | 0.938594946791208 | 0.939062298756104 | 0.931333091738645 | 0.940390893911036 | 0.922321803697842 |
| 15-Sep | 0.963079231370524 | 0.888936270578801 | 0.943633360169115 | 0.858849706676629 | 0.936537144053459 | 0.874350243197029 |
| 15-Oct | 0.905642445611492 | 0.891582120191593 | 0.933179887520151 | 0.894424506142712 | 0.947200949006391 | 0.929562189365694 |
| 15-Nov | 0.928817760997898 | 0.899724198336946 | 0.934030122572777 | 0.892746590060904 | 0.931510208866758 | 0.904927690903753 |
| 15-Dec | 0.891469180152815 | 0.902821705781532 | 0.919157113343297 | 0.877339477909194 | 0.93665100623502 | 0.877929666934904 |
| 16-Jan | 0.86643661317426 | 0.863021930932865 | 0.861110889874507 | 0.842975820872049 | 0.864084338602624 | 0.873789268653811 |
| 16-Feb | 0.895259016700102 | 0.887815420049737 | 0.89565049690194 | 0.898270100298902 | 0.913885399560908 | 0.896597635815438 |
| 16-Mar | 0.925511132010067 | 0.91925296052441 | 0.927686012920896 | 0.912007213603975 | 0.934660009929343 | 0.901975797390338 |
| 16-Apr | 0.946520569743528 | 0.941977476058757 | 0.947446784723078 | 0.921376223536581 | 0.941340450660888 | 0.928985945859418 |
| 16-May | 0.923345567049664 | 0.942700563095743 | 0.931422937718942 | 0.93727357075132 | 0.876513292301366 | 0.918711634690663 |
| 16-Jun | 0.927080839111146 | 0.94337066679163 | 0.912757078056005 | 0.954058091389973 | 0.887117594648121 | 0.951827157246746 |
| 16-Jul | 0.927940824018485 | 0.941224005087038 | 0.929083442108978 | 0.928727487771043 | 0.927220873279258 | 0.926295863841548 |
| 16-Aug | 0.938664856862794 | 0.947010668561871 | 0.932741267843255 | 0.935822469957789 | 0.930874173054575 | 0.931908909218482 |
| 16-Sep | 0.950811643575458 | 0.895010294729415 | 0.950731439865075 | 0.838528771919731 | 0.923540651108535 | 0.812622529233794 |
| 16-Oct | 0.92325374760176 | 0.919419170910701 | 0.935172073919715 | 0.903724832635445 | 0.941495439844521 | 0.912039917372406 |
| 16-Nov | 0.933516232460165 | 0.935039695782915 | 0.929275786324434 | 0.928377535048272 | 0.927825790545556 | 0.937656951273512 |
| 16-Dec | 0.90959154591892 | 0.916169470176056 | 0.905095837546993 | 0.90398340385252 | 0.919555624823737 | 0.912264383448546 |
| 17-Jan | 0.901208773580259 | 0.901047732245311 | 0.904974508903035 | 0.901058346548176 | 0.909013377656011 | 0.910573363960405 |
| 17-Feb | 0.929474999294603 | 0.931981095439295 | 0.932045344896791 | 0.933260649766597 | 0.935443265716717 | 0.93398541832954 |
| 17-Mar | 0.923671264875021 | 0.936596900380317 | 0.92673263388042 | 0.932798877888022 | 0.931955047192561 | 0.923148171257611 |
| 17-Apr | 0.939349088889437 | 0.935905769302918 | 0.938245051601948 | 0.933844283057815 | 0.941736757405726 | 0.929049747874649 |
| 17-May | 0.94779705579357 | 0.945754077789566 | 0.924321305855562 | 0.935810582501776 | 0.940492453031212 | 0.93663054463722 |
| 17-Jun | 0.928653249114149 | 0.943065747724581 | 0.924641298354543 | 0.923086265869063 | 0.931637003226727 | 0.914716170860575 |
| 17-Jul | 0.948612824380006 | 0.949889830638461 | 0.948963412882927 | 0.912499911292654 | 0.95134717671679 | 0.915825642842735 |
| 17-Aug | 0.943750219513055 | 0.901053256084302 | 0.898827869182918 | 0.890288504700191 | 0.928853579938381 | 0.88018757803106 |
| 17-Sep | 0.958795395741054 | 0.938499928033004 | 0.957409068811755 | 0.941328053493176 | 0.952365568394151 | 0.948572019870792 |
| 17-Oct | 0.922522105525581 | 0.911012544304419 | 0.944762377119724 | 0.926521586244396 | 0.945805493102202 | 0.936100885124489 |
| 17-Nov | 0.931867508904132 | 0.936365731403 | 0.938173822529481 | 0.923628595973906 | 0.93661664012638 | 0.924822568825699 |
| 17-Dec | 0.917149915273961 | 0.9014438760157 | 0.91742581633068 | 0.906251941011562 | 0.919108724197944 | 0.914140564640391 |
| 18-Jan | 0.887584655263759 | 0.88197550595078 | 0.887823997422918 | 0.888006176156699 | 0.903887961630483 | 0.892625793312346 |
| 18-Feb | 0.883769059085724 | 0.882093880406773 | 0.888857421325017 | 0.893458917141377 | 0.899364638144723 | 0.899808827777546 |
| 18-Mar | 0.883512350289243 | 0.950418845044952 | 0.850965921070573 | 0.931966387489844 | 0.900386339153085 | 0.936205184065589 |
| 18-Apr | 0.935793104897097 | 0.92861801637203 | 0.943672683084283 | 0.933034883379527 | 0.94721027762772 | 0.934807519896812 |
| 18-May | 0.93125552738499 | 0.925662447370984 | 0.926931057160624 | 0.930731970778914 | 0.938685149724159 | 0.9360689724377 |
| 18-Jun | 0.93505791753851 | 0.933560111201377 | 0.939599429709449 | 0.93796283125988 | 0.92931327316808 | 0.943221839335067 |
| 18-Jul | 0.928064851575883 | 0.954077285578406 | 0.933852080696372 | 0.948289963105799 | 0.939314624906602 | 0.955241979916664 |
| 18-Aug | 0.954396053751984 | 0.958912314639341 | 0.953832885645825 | 0.956044582986549 | 0.953851720513041 | 0.959804536003287 |
| 18-Sep | 0.960181875405326 | 0.948239057890813 | 0.957772937635032 | 0.949718435368668 | 0.956805421944432 | 0.954646497235874 |
| 18-Oct | 0.95579322694985 | 0.941940603801808 | 0.952449821790324 | 0.947011323453102 | 0.954698101971721 | 0.94201882996455 |
| 18-Nov | 0.937710218025613 | 0.929799013280375 | 0.942183744928675 | 0.921512341453424 | 0.951280880605505 | 0.934402058051469 |
| 18-Dec | 0.922455713559057 | 0.913422690004428 | 0.916970637359919 | 0.916621720002329 | 0.935224463333124 | 0.928296508222085 |
| 19-Jan | 0.891624363817897 | 0.901909727791752 | 0.89353373661994 | 0.89327545098756 | 0.892768219965567 | 0.895148334427376 |
| 19-Feb | 0.923879625997325 | 0.917804045184555 | 0.921766975244393 | 0.925419579287769 | 0.917402241752599 | 0.916056014554708 |
| 19-Mar | 0.928711533148656 | 0.925177791527767 | 0.903858668640856 | 0.932938873357402 | 0.924557872529147 | 0.925540646733734 |
| 19-Apr | 0.94432096394235 | 0.937673111293971 | 0.944521397084914 | 0.924561171124354 | 0.945286606926587 | 0.923229802415278 |
| 19-May | 0.936253506644062 | 0.956855646740609 | 0.907441318509408 | 0.930978356992235 | 0.909201718248891 | 0.938723567558222 |
| 19-Jun | 0.922500821325701 | 0.927138724383328 | 0.930109767407206 | 0.942890633652464 | 0.91173608597277 | 0.948859367366515 |
| 19-Jul | 0.893543184937232 | 0.919975212285474 | 0.890347206431855 | 0.943907305280465 | 0.884887032713762 | 0.948736496492322 |
| 19-Aug | 0.941166812608861 | 0.94651484188207 | 0.945623078736355 | 0.952704251236783 | 0.93464137244875 | 0.951290965962931 |
| 19-Sep | 0.946763634610664 | 0.92947513803825 | 0.952518885105012 | 0.905767705155734 | 0.949598981193712 | 0.912905597676156 |
| 19-Oct | 0.946856709641684 | 0.937793270669089 | 0.947135882301901 | 0.947485516752831 | 0.949873048734172 | 0.947206064969697 |
| 19-Nov | 0.951732422347063 | 0.940380075157077 | 0.95219629644585 | 0.954873175003837 | 0.954008054696137 | 0.957262082752356 |
| 19-Dec | 0.932472295245786 | 0.924473426454874 | 0.915968230055904 | 0.930993962458654 | 0.917331161719154 | 0.92594157413341 |B

## Slide 13
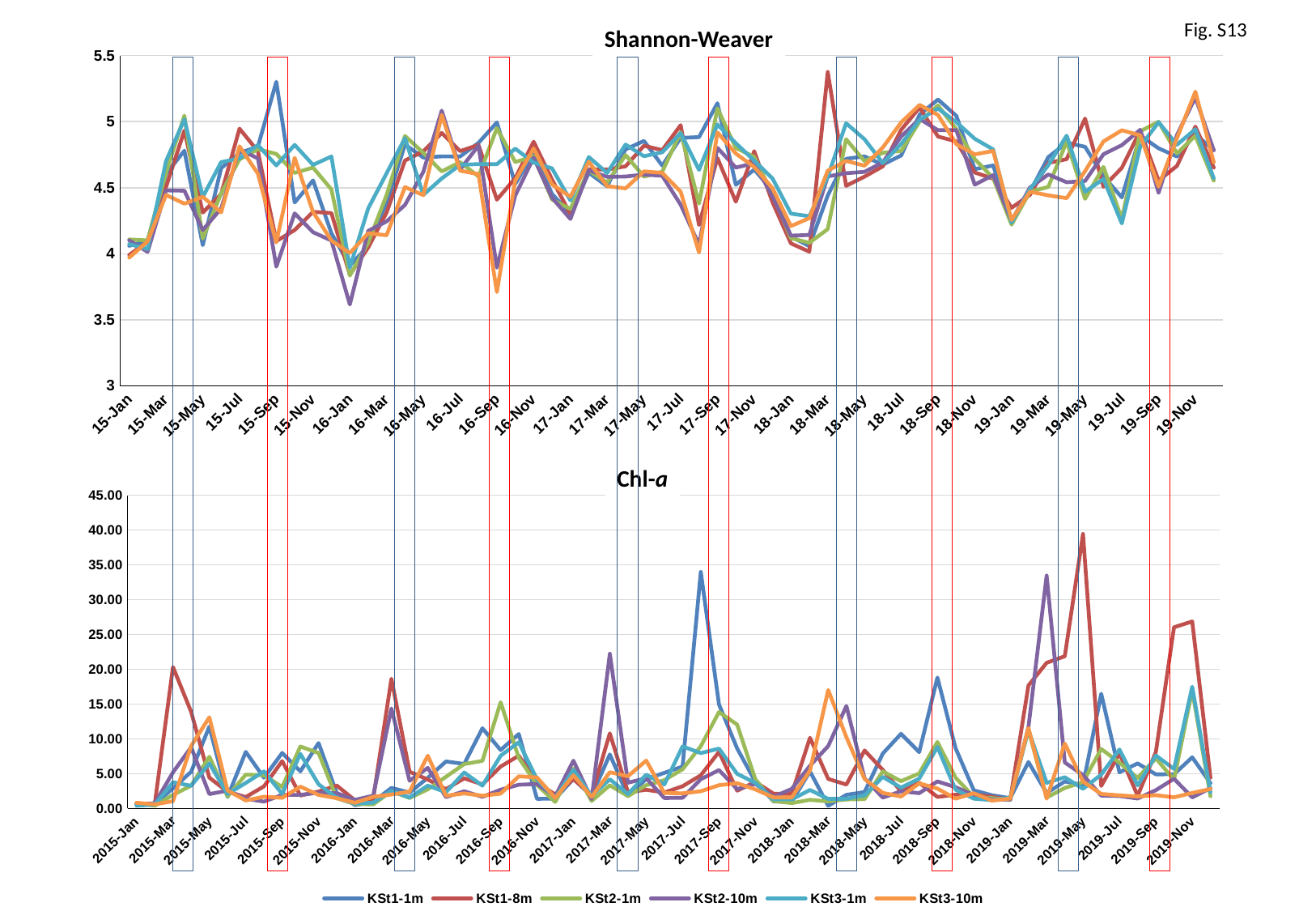

Fig. S13
Shannon-Weaver
### Chart
| Category | KSt1-1m | KSt1-8m | KSt2-1m | KSt2-10m | KSt3-1m | KSt3-10m |
|---|---|---|---|---|---|---|
| 15-Jan | 4.06022644487682 | 3.99063268067338 | 4.10826247037528 | 4.10155448504764 | 4.07310370958567 | 3.97079841162962 |
| 15-Feb | 4.09957813967345 | 4.091466166466999 | 4.10112472440283 | 4.013904009938159 | 4.03538476323024 | 4.093592240886419 |
| 15-Mar | 4.60461098053963 | 4.50515201363201 | 4.614320219869879 | 4.47984417755746 | 4.700854540072579 | 4.44521067043475 |
| 15-Apr | 4.78024541781951 | 4.932936239269059 | 5.045470653005449 | 4.477154832425659 | 5.017821507178689 | 4.37891983495912 |
| 15-May | 4.065124046210959 | 4.31171172366284 | 4.11475362914303 | 4.17968330351378 | 4.429084987666589 | 4.43080495316511 |
| 15-Jun | 4.648407297865988 | 4.45182887434299 | 4.45769529214166 | 4.342898331279589 | 4.693395371317049 | 4.313625867697019 |
| 15-Jul | 4.76322429604294 | 4.94697421394985 | 4.7142720214592 | 4.78968722719255 | 4.72186762893009 | 4.813583207771899 |
| 15-Aug | 4.810493259763999 | 4.77594988313631 | 4.79163119797672 | 4.721896274970549 | 4.823402230643749 | 4.605955076637058 |
| 15-Sep | 5.30153351442998 | 4.09570342642302 | 4.75547508802423 | 3.90165785457739 | 4.668170071471329 | 4.0859221869508 |
| 15-Oct | 4.38810326532567 | 4.17917409113894 | 4.612308531776438 | 4.30552834628137 | 4.82396188397315 | 4.725401851586069 |
| 15-Nov | 4.554895890587849 | 4.317034206873558 | 4.65242990167422 | 4.162780887797008 | 4.672801823698249 | 4.315754869255628 |
| 15-Dec | 4.15510369485692 | 4.307813472579379 | 4.48656974554264 | 4.10005669999184 | 4.73620544079852 | 4.10028400766432 |
| 16-Jan | 3.90913780978281 | 3.840845492108969 | 3.83436174603079 | 3.615452886954079 | 3.89438364485526 | 4.004710269475938 |
| 16-Feb | 4.058581341575199 | 4.047301337032409 | 4.083131407776249 | 4.172974520798139 | 4.34368834717331 | 4.155236273355448 |
| 16-Mar | 4.3862313211416 | 4.311776819755379 | 4.443887679476769 | 4.24486569842747 | 4.608857612029769 | 4.139954625877059 |
| 16-Apr | 4.826376877473019 | 4.70575562598656 | 4.89177019195273 | 4.36923745923498 | 4.87488001513687 | 4.504791367340219 |
| 16-May | 4.72838249194643 | 4.774838788475489 | 4.763876698369049 | 4.61969971008537 | 4.44583978262595 | 4.44365650851924 |
| 16-Jun | 4.73724696184547 | 4.914314344625229 | 4.623562977789208 | 5.08353640806216 | 4.57012893800111 | 5.05086233644067 |
| 16-Jul | 4.73645954362272 | 4.77705177957587 | 4.693334313763589 | 4.63774937865986 | 4.672785304598479 | 4.62879647439289 |
| 16-Aug | 4.8392125346839 | 4.823433318651219 | 4.58532267243221 | 4.81840727216463 | 4.67877618035858 | 4.6024413019916 |
| 16-Sep | 4.99362318627384 | 4.40906328542807 | 4.95070659201537 | 3.89433478946646 | 4.67794502000867 | 3.7099639600606 |
| 16-Oct | 4.527399302295459 | 4.57196852976092 | 4.694435452410198 | 4.44088635488211 | 4.795830277304389 | 4.55340999967547 |
| 16-Nov | 4.76667146225534 | 4.848232656199759 | 4.736511849170229 | 4.727134542295417 | 4.690008574696779 | 4.797535771677598 |
| 16-Dec | 4.45190970466234 | 4.5614755883836 | 4.41070889689886 | 4.423502772087419 | 4.646180019033929 | 4.525477236065948 |
| 17-Jan | 4.305113502100389 | 4.3004025318733 | 4.33934652337257 | 4.26318170438828 | 4.40356192878228 | 4.43152317242415 |
| 17-Feb | 4.61346463169476 | 4.63959795358987 | 4.630326374205039 | 4.63160774392071 | 4.732110939718408 | 4.69670387418918 |
| 17-Mar | 4.510015040078288 | 4.63767091149161 | 4.537589917880909 | 4.58199070610075 | 4.611087143395689 | 4.51148212249621 |
| 17-Apr | 4.790234302230559 | 4.66185177376052 | 4.7473607967731 | 4.58428003596048 | 4.826566838460748 | 4.49478301552095 |
| 17-May | 4.854105972484908 | 4.81766165965292 | 4.58375036571375 | 4.59992018495992 | 4.73848946583204 | 4.623431232239369 |
| 17-Jun | 4.666954351397678 | 4.78413019110797 | 4.621355510194839 | 4.58996809977608 | 4.768177427290209 | 4.61227315021129 |
| 17-Jul | 4.87691753488868 | 4.97360671717665 | 4.9192320939842 | 4.37162066976279 | 4.91360631543668 | 4.46979822654936 |
| 17-Aug | 4.883018206701559 | 4.217486693926479 | 4.37958173107225 | 4.06844990147919 | 4.6347366859861 | 4.009211260437939 |
| 17-Sep | 5.13946579357017 | 4.724865868943349 | 5.100813678479319 | 4.80152529351693 | 4.97861329562899 | 4.918913511446109 |
| 17-Oct | 4.52154895444205 | 4.39392373449801 | 4.8012990019422 | 4.65219301502462 | 4.83667909790638 | 4.75827269273855 |
| 17-Nov | 4.63716345160137 | 4.77644383042608 | 4.733984822526339 | 4.684946456496558 | 4.705486240719439 | 4.65672829929477 |
| 17-Dec | 4.47758548339976 | 4.388562859149259 | 4.45160018416252 | 4.43268167699126 | 4.569011465288999 | 4.495314778265349 |
| 18-Jan | 4.13022847713812 | 4.0783292652473 | 4.117586562092238 | 4.137127256357448 | 4.303595202333419 | 4.209184251048509 |
| 18-Feb | 4.057516573118987 | 4.01529464484859 | 4.08296305512294 | 4.14198195419987 | 4.28375111909008 | 4.26930510073963 |
| 18-Mar | 4.44078571617143 | 5.37635561103718 | 4.18650229088823 | 4.585575562866859 | 4.58240515412017 | 4.628978767033539 |
| 18-Apr | 4.71854219024863 | 4.514238550204809 | 4.86697545474933 | 4.60950349510011 | 4.98806229033471 | 4.70236800169977 |
| 18-May | 4.737130680488009 | 4.58349167507625 | 4.70136186434763 | 4.61991073473134 | 4.867111202062149 | 4.666928851753379 |
| 18-Jun | 4.67427270136257 | 4.661910090013259 | 4.764385778099508 | 4.691694728006989 | 4.685582870384298 | 4.80470514508168 |
| 18-Jul | 4.744575440542509 | 4.93931857587712 | 4.77846780680956 | 4.888854800737269 | 4.83138312296419 | 4.99474263157159 |
| 18-Aug | 5.053185804977779 | 5.10640330757154 | 5.00143346991221 | 5.02023800899703 | 5.01506959319357 | 5.12678929688417 |
| 18-Sep | 5.167450764259879 | 4.88681376277667 | 5.12277614095133 | 4.93515016618183 | 5.099016278760019 | 5.050214764299939 |
| 18-Oct | 5.04334841023905 | 4.850359649852249 | 4.94207620358192 | 4.93504200945624 | 4.995561662825899 | 4.82944770016418 |
| 18-Nov | 4.64049202346077 | 4.615146921151509 | 4.71685935526884 | 4.52323401475142 | 4.87025774584898 | 4.75245438902417 |
| 18-Dec | 4.66837041082264 | 4.567975542771398 | 4.57324114263113 | 4.5946913077838 | 4.789897416798 | 4.777931813889209 |
| 19-Jan | 4.242142616839589 | 4.347963833440419 | 4.22031611753437 | 4.247514994265889 | 4.23559183387938 | 4.255521267264859 |
| 19-Feb | 4.46812839031913 | 4.44536746649339 | 4.46946986693439 | 4.50072130410516 | 4.48879836507578 | 4.46953168435001 |
| 19-Mar | 4.7317989639176 | 4.6876684582828 | 4.50583034370461 | 4.60039628269486 | 4.67072240289026 | 4.442125360265689 |
| 19-Apr | 4.84068772213508 | 4.71618229170446 | 4.84919458597099 | 4.54119998001246 | 4.893906066896938 | 4.420544661148139 |
| 19-May | 4.80962489122222 | 5.02300148694388 | 4.41562781263872 | 4.550788249598739 | 4.46927163719703 | 4.625413737108048 |
| 19-Jun | 4.5866696763724 | 4.50710294268426 | 4.657489486617299 | 4.75292012268476 | 4.5658940500348 | 4.85007200322963 |
| 19-Jul | 4.426456318206769 | 4.64971140663216 | 4.26194193567252 | 4.822070399084939 | 4.229327466916399 | 4.934574224535999 |
| 19-Aug | 4.894350972684039 | 4.93183087390148 | 4.92681201057458 | 4.93831643654105 | 4.81106895301077 | 4.89629290887308 |
| 19-Sep | 4.79825743645028 | 4.553807579095408 | 4.99965339837266 | 4.46129965068909 | 4.99538345547698 | 4.50905018741204 |
| 19-Oct | 4.73572299764412 | 4.66516966384238 | 4.75942703850858 | 4.899156581769589 | 4.825436438271839 | 4.875007224905989 |
| 19-Nov | 4.90508278178604 | 4.962385470739569 | 4.893049630425279 | 5.18288798629882 | 4.94264218883062 | 5.227355863518948 |
| 19-Dec | 4.78360972530338 | 4.65230165594866 | 4.55369676154646 | 4.784189029755639 | 4.57230437198258 | 4.69264335008931 |
### Chart: Chl-a
| Category | KSt1-1m | KSt1-8m | KSt2-1m | KSt2-10m | KSt3-1m | KSt3-10m |
|---|---|---|---|---|---|---|
| 2015-Jan | 0.564 | 0.709 | 0.753 | 0.648 | 0.375 | 0.8 |
| 2015-Feb | 0.673 | 0.613 | 0.373 | 0.716 | 0.541 | 0.576 |
| 2015-Mar | 2.858999999999999 | 20.318 | 1.927999999999999 | 5.172 | 3.752 | 1.029 |
| 2015-Apr | 5.26 | 13.875 | 3.271 | 8.783000000000001 | 3.245 | 8.963000000000003 |
| 2015-May | 11.752 | 4.417 | 7.419 | 2.048 | 6.458 | 13.086 |
| 2015-Jun | 1.641 | 2.389 | 1.713 | 2.555 | 2.058 | 2.576 |
| 2015-Jul | 8.110999999999999 | 1.625 | 4.847999999999999 | 1.39 | 3.653 | 1.132 |
| 2015-Aug | 4.4 | 3.164 | 4.758 | 0.963 | 5.311999999999999 | 1.672 |
| 2015-Sep | 7.961 | 6.793 | 3.063 | 1.937999999999999 | 2.078 | 1.514 |
| 2015-Oct | 5.312999999999999 | 1.835 | 8.886000000000003 | 1.933 | 7.828999999999999 | 3.124 |
| 2015-Nov | 9.387 | 2.416999999999999 | 7.953 | 2.254 | 3.461 | 1.925999999999999 |
| 2015-Dec | 2.185 | 3.277 | 1.461 | 2.179 | 1.504 | 1.452 |
| 2016-Jan | 0.46 | 1.16 | 0.62 | 1.23 | 0.69 | 0.8 |
| 2016-Feb | 0.93 | 1.36 | 0.57 | 1.82 | 0.82 | 1.66 |
| 2016-Mar | 2.94 | 18.63 | 2.47 | 14.35 | 2.5 | 1.98 |
| 2016-Apr | 2.27 | 5.26 | 1.49 | 3.96 | 1.56 | 2.4 |
| 2016-May | 4.39 | 4.149999999999999 | 2.73 | 5.819999999999999 | 3.26 | 7.57 |
| 2016-Jun | 6.74 | 2.76 | 4.53 | 1.62 | 2.319999999999999 | 1.81 |
| 2016-Jul | 6.38 | 4.31 | 6.37 | 2.46 | 5.149999999999999 | 2.15 |
| 2016-Aug | 11.51 | 3.44 | 6.819999999999999 | 1.65 | 3.26 | 1.81 |
| 2016-Sep | 8.38 | 6.0 | 15.24 | 2.68 | 7.56 | 2.12 |
| 2016-Oct | 10.68 | 7.56 | 7.26 | 3.39 | 9.5 | 4.6 |
| 2016-Nov | 1.37 | 3.64 | 3.37 | 3.51 | 4.17 | 4.45 |
| 2016-Dec | 1.39 | 2.0 | 0.92 | 1.73 | 1.48 | 1.48 |
| 2017-Jan | 4.22 | 4.21 | 5.72 | 6.85 | 5.53 | 4.75 |
| 2017-Feb | 1.78 | 1.93 | 1.07 | 1.34 | 1.77 | 1.65 |
| 2017-Mar | 7.75 | 10.78 | 3.31 | 22.23 | 4.18 | 5.189999999999999 |
| 2017-Apr | 1.76 | 2.31 | 1.76 | 3.69 | 2.06 | 4.619999999999999 |
| 2017-May | 4.21 | 2.67 | 3.33 | 4.33 | 4.84 | 6.859999999999999 |
| 2017-Jun | 5.1 | 2.27 | 4.05 | 1.48 | 3.43 | 2.2 |
| 2017-Jul | 6.01 | 3.18 | 5.619999999999999 | 1.52 | 8.9 | 2.16 |
| 2017-Aug | 33.97 | 4.75 | 8.93 | 4.18 | 7.94 | 2.46 |
| 2017-Sep | 14.93 | 8.11 | 13.84 | 5.51 | 8.59 | 3.33 |
| 2017-Oct | 8.61 | 2.53 | 12.04 | 2.76 | 4.96 | 3.61 |
| 2017-Nov | 3.81 | 3.82 | 4.18 | 3.5 | 3.65 | 2.72 |
| 2017-Dec | 0.99 | 2.1 | 1.05 | 1.66 | 1.29 | 1.58 |
| 2018-Jan | 0.99 | 2.1 | 0.76 | 2.8 | 1.29 | 1.58 |
| 2018-Feb | 5.3 | 10.15 | 1.22 | 6.13 | 2.64 | 5.43 |
| 2018-Mar | 0.37 | 4.23 | 1.0 | 8.95 | 1.36 | 17.0 |
| 2018-Apr | 1.94 | 3.42 | 1.28 | 14.71 | 1.38 | 10.23 |
| 2018-May | 2.35 | 8.32 | 1.33 | 4.319999999999999 | 1.87 | 4.22 |
| 2018-Jun | 7.88 | 5.52 | 5.3 | 1.54 | 4.52 | 2.22 |
| 2018-Jul | 10.71 | 2.44 | 3.91 | 2.46 | 2.97 | 1.7 |
| 2018-Aug | 8.08 | 3.69 | 5.02 | 2.18 | 4.27 | 3.59 |
| 2018-Sep | 18.8 | 1.64 | 9.540000000000001 | 3.87 | 8.83 | 2.84 |
| 2018-Oct | 8.639999999999999 | 1.96 | 4.41 | 3.04 | 2.68 | 1.39 |
| 2018-Nov | 2.59 | 2.0 | 1.64 | 1.89 | 1.39 | 2.17 |
| 2018-Dec | 1.88 | 1.65 | 1.47 | 1.28 | 1.18 | 1.11 |
| 2019-Jan | 1.467222756752635 | 1.230238018146694 | 1.201212254792419 | 1.270856388206488 | 1.546335660529226 | 1.412887943644328 |
| 2019-Feb | 6.660976040367743 | 17.65089993697061 | 11.30717776680427 | 11.6579647361224 | 11.27425830348783 | 11.56504689611634 |
| 2019-Mar | 2.201583418933889 | 20.92209461767931 | 1.556556622929893 | 33.45795013903066 | 3.668844605810588 | 1.389923963185686 |
| 2019-Apr | 3.861848233480324 | 21.86538768010277 | 2.932006134562504 | 6.596066292134937 | 4.47152200369154 | 9.305462013530788 |
| 2019-May | 3.374 | 39.438 | 3.767 | 4.792 | 2.805 | 3.86 |
| 2019-Jun | 16.472 | 3.222 | 8.530000000000001 | 1.832 | 4.855999999999999 | 2.074 |
| 2019-Jul | 5.177564799999999 | 7.8960311 | 6.571598 | 1.768719 | 8.459290200000003 | 1.86526508 |
| 2019-Aug | 6.4568223 | 1.8177885 | 4.367497999999999 | 1.410233 | 3.2991643 | 1.70858791 |
| 2019-Sep | 4.8894311 | 8.0873534 | 7.2008287 | 2.612147499999999 | 7.597853299999999 | 1.89362715 |
| 2019-Oct | 4.900935199999999 | 26.026072 | 4.108788499999999 | 4.247589999999999 | 5.660250899999999 | 1.59350253 |
| 2019-Nov | 7.338 | 26.864 | 16.982 | 1.569 | 17.482 | 2.212 |
| 2019-Dec | 3.616412399999999 | 4.4064353 | 1.7391181 | 2.911873899999999 | 2.2962711 | 2.77851471 |Chl-a
